# Health-related quality of life in patients undergoing laparoscopic versus open hemihepatectomy: a secondary analysis of the ORANGE II PLUS randomised controlled, phase 3, superiority trial

**DOI:** 10.1016/j.lanepe.2025.101311

**Published:** 2025-05-19

**Authors:** Bram Olij, Robert S. Fichtinger, Luca A. Aldrighetti, Mohammad Abu Hilal, Roberto I. Troisi, Robert P. Sutcliffe, Marc G. Besselink, Somaiah Aroori, Krishna V. Menon, Bjørn Edwin, Mathieu D’Hondt, Valerio Lucidi, Tom F. Ulmer, Rafael Díaz-Nieto, Zahir Soonawalla, Steve White, Gregory Sergeant, Francesca Ratti, Christoph Kuemmerli, Vincenzo Scuderi, Frederik Berrevoet, Aude Vanlander, Ravi Marudanayagam, Pieter J. Tanis, Maxime J.L. Dewulf, Zina B. Eminton, Ulf P. Neumann, Lloyd Brandts, Siân A. Pugh, Åsmund A. Fretland, Merel L. Kimman, John N. Primrose, Ronald M. van Dam, ORANGE II PLUS Collaborativead, Ronald M. Van Dam, Luca A. Aldrighetti, Mohammed Abu Hilal, Roberto I. Troisi, Robert P. Sutcliffe, Marc G. Besselink, Somaiah Aroori, Krishna V. Menon, Bjørn Edwin, Mathieu D’Hondt, Valerio Lucidi, Tom F. Ulmer, Rafael Diaz-Nieto, Zahir Soonawalla, Steve White, Gregory Sergeant, Robert S. Fichtinger, Bram Olij, Francesca Ratti, Christoph Kuemmerli, Vincenzo Scuderi, Frederik Berrevoet, Aude Vanlander, Ravi Marudanayagam, Pieter J. Tanis, Maxime J.L. Dewulf, Zina B. Eminton, Ulf P. Neumann, Lloyd Brandts, Siân A. Pugh, Åsmund A. Fretland, Merel L. Kimman, John N. Primrose, Remon Korenblik, Michelle Lintforth, Burak Gorçek, Penelope Rogers, Viviane Van Laethem, Betsy Van Loo, Kathleen Segers, Celine Demeyere, Ane Zamalloa, Cornelis Dejong, Davit Aghayan, Katherine Gordon-Quayle, Tracy Ward, Jess Boxal, Beth Wedge

**Affiliations:** a Department of Surgery, Maastricht University Medical Centre+, Maastricht, the Netherlands; b GROW – Research Institute for Oncology and Reproduction, Maastricht University, Maastricht, the Netherlands; c Hepatobiliary Surgery Division, IRCCS San Raffaele Hospital, Milan, Italy; d Department of Surgery, School of Medicine, The University of Jordan, Amman 11942, Jordan; e University Surgery and Perioperative and Critical Care Theme, NIHR Southampton Biomedical Research Centre, University Hospital Southampton / University of Southampton, Southampton, UK; f Department of Surgery, Poliambulanza Hospital, Brescia, Italy; g Division of HPB, Minimally Invasive and Robotic Surgery, Federico II University, Naples, Italy; h HPB and Liver Transplant Unit, University Hospitals Birmingham NHS Trust, Birmingham, United Kingdom; i Department of Surgery, Amsterdam UMC, location University of Amsterdam, Amsterdam, the Netherlands; j Cancer Centre Amsterdam, the Netherlands; k Department of Surgery, Plymouth Hospitals NHS Trust, Plymouth, United Kingdom; l Department of Liver Transplant and HPB Surgery, Institute of Liver Studies, King's College Hospital, NHS Foundation Trust, London, United Kingdom; m Intervention Centre and Department of Hepatic, Pancreatic and Biliary Surgery, Oslo University Hospital and Institute of Medicine, University of Oslo, Oslo, Norway; n Department of Digestive and Hepatobiliary/Pancreatic Surgery, AZ Groeninge, Kortrijk, Belgium; o Department of Digestive Surgery, Unit of Hepatobiliary Surgery and Transplantation, Hôpitaux Universitaires de Bruxelles, ULB-Université Libre de Bruxelles, Brussels, Belgium; p Department of Surgery and Transplantation, University Hospital RWTH Aachen, Aachen, Germany; q Department of Surgery, University Hospital Essen, Essen, Germany; r Department of Hepato-Biliary Surgery, Aintree University Hospital NHS Foundation Trust, Liverpool, United Kingdom; s Department of Surgery, Oxford University Hospitals NHS Foundation Trust, Oxford, United Kingdom; t Department of Surgery, Newcastle Upon Tyne Hospitals NHS Foundation Trust, Newcastle upon Tyne, United Kingdom; u Department of Digestive and Hepatobiliary/Pancreatic Surgery, Jessa Hospital, Hasselt, Belgium; v Faculty of Medicine and Health Sciences, UHasselt, Hasselt, Belgium; w Clarunis University Digestive Health Care Centre Basel, University Hospital Basel, Basel, Switzerland; x Department for General and HPB Surgery and Liver Transplantation, Ghent University Hospital, Ghent, Belgium; y Department of Surgery, Free University Hospital, AZ Jette Hospital, Brussels, Belgium; z Southampton Clinical Trials Unit, University of Southampton, Southampton, United Kingdom; aa Department of Clinical Epidemiology and Medical Technology Assessment, Maastricht University Medical Centre+, Maastricht, the Netherlands; ab Department of Oncology, Addenbrooke’s Hospital, Cambridge, United Kingdom

**Keywords:** Quality of life, Laparoscopic hepatectomy, RCT

## Abstract

**Background:**

Health-related quality of life (HRQoL) has become a critical factor in determining the benefits of new surgical approaches on patients. The ORANGE II PLUS randomised trial compared laparoscopic (LH) and open (OH) hemihepatectomy in an international multicentre randomised controlled setting, with HRQoL as a secondary outcome. The aim of this study was to perform an in-depth analysis of the HRQoL outcomes.

**Methods:**

Between October 2013 and January 2019, 352 patients scheduled for hemihepatectomy, were randomly assigned to either LH or OH in a 1:1-ratio in 16 European centres. HRQoL was assessed using the EORTC-QLQ-C30 and QLQ-LMC21 modules, at baseline, hospital discharge, and at 10-days, 3-, 6-, and 12-months after discharge. Differences in functioning- and five selected symptom scales were compared between LH and OH over the cumulative periods from discharge to 3 months as well as to 12 months using a multivariable adjusted linear mixed regression model. The study was registered at ClinicalTrials.gov (NCT01441856).

**Findings:**

The modified intention-to-treat analysis included 332 patients (166 LH and 166 OH), with 40% female in LH and 42% female in OH. 1546 questionnaires (81% of maximum) were obtained. Cumulatively over the period from discharge to 3 months postoperatively, patients in the laparoscopic group reported better physical (difference 4.20 points; 95% CI 1.07–7.34) and social functioning (5.95 points; 95% CI 1.65–10.26), and lower pain (−6.41 points, 95% CI −10.01 to −2.82) and appetite loss (−7.29 points, 95% CI −11.59 to −2.99), compared to the OH group. Similar clinically relevant, but slightly attenuated, differences were reported over the cumulative period from discharge to 12 months after surgery. The largest difference was observed at 10 days after surgery.

**Interpretation:**

In this international randomised trial evaluating HRQoL, LH demonstrated better physical and social functioning, and less pain and appetite loss, compared to OH. These findings support the preferential use of the laparoscopic approach for hemihepatectomy in experienced centres.

**Funding:**

Maastricht University Medical Centre+, University Hospital RWTH Aachen, Cancer Research UK 12/048, 10.13039/501100012161European Association of Endoscopic Surgery, participating centres.

**Research in context**

**Evidence before this study**

Over the last decades, enhanced recovery after surgery programmes reduced postoperative complication, shortened recovery time and improved health-related quality of life. An important component of these programmes is a minimally invasive surgical strategy that aims to lower the physical impact of liver resection. Data on the effect of minimally invasive liver surgery on patients’ health-related quality of life are scarce. However, in the OSLO-COMET randomised trial patients who received minimally invasive surgery for minor liver resections reported better postoperative health-related quality of life as compared to the open surgical approach. In the recently published ORANGE II PLUS trial patients who underwent laparoscopic major liver surgery reported a better global health score than patients who received open surgery, yet in-depth analysis of health-related quality of life is missing. We searched PubMed and the Cochrane Library in 2011 and updated the search on September 13th, 2024, with the search terms: (“hemihepatectomy” OR “major liver surgery”) AND (“laparoscopy” OR “minimally invasive liver surgery”) AND (“randomised trial” OR “meta-analysis” OR “systematic review”) AND (“HRQoL” OR “Health-related quality of life” OR “quality of life”) with no language restrictions. The published comparative literature for open and laparoscopic major liver surgery consists of case studies and prospective observational studies. The most recent data indicates that laparoscopic hemihepatectomy is associated with better overall Health-related quality of life, and better physical and social functioning. While many centres currently apply the laparoscopic approach for major liver surgery to further enhance postoperative recovery, worldwide adoption of the technique should be based on a higher level of evidence, as well as patient-reported data, preferably one or more randomised controlled trials.

**Added value of this study**

To the best of our knowledge, this is the first randomised controlled trial to compare patient-reported health-related quality of life in laparoscopic and open hemihepatectomy. This trial shows that cumulatively over the period from discharge to 3 months postoperatively there was a clinically relevant difference observed in physical functioning, difference 4.20 points (95% CI 1.07–7.34), cognitive functioning, difference 3.11 points (95% CI 0.17–6.40), and in social functioning with a difference of 5.95 points (95% CI 1.65–10.26), as well as clinically relevant overall differences for the domains ‘pain’ (−6.41 points, 95% CI −10.01 to −2.82), ‘appetite loss’ (−7.29 points, 95% CI −11.59 to −2.99), and ‘nutritional problems’ (−4.37 points, 95% CI −7.96 to −0.78) all in favour of the laparoscopic approach, with no clinically relevant advantages found for the open approach.

**Implications of all the available evidence**

The findings of this trial demonstrate that, in addition to the previously shown shortened time to functional recovery, earlier time to hospital discharge and earlier start of adjuvant chemotherapy, laparoscopic hemihepatectomy is also superior in terms of postoperative physical-, cognitive-, and social functioning as well as less bodily pain, appetite loss and nutritional problems up to 3 months postoperatively. Patients in need for hemihepatectomy and eligible to the laparoscopic approach benefit most if operated laparoscopically in experienced centres that have an enhanced recovery after surgery programme in place.

## Introduction

Surgical resection remains the primary treatment for malignant, pre-malignant and symptomatic benign liver lesions covering a significant surface of the liver tissue.^1^ Continuous advancements in peri-operative care and enhanced recovery after surgery (ERAS) programmes have enabled more complex liver resections, expanding eligibility for surgery and improving short- and long-term outcomes.^2^^,^^3^ In cancer patients, part of this improvement relates to the increased use of peri-operative systemic therapy, often involving multiple anti-cancer drugs to achieve synergetic effects. However, both systemic therapy and surgery impact the patients’ health-related quality of life (HRQoL), and as patients frequently need repeated treatments, over time these can have serious cumulative effects. While minimally invasive liver resection reduces the cumulative physical impact of cancer treatment on patients, surgery itself has an inherent negative effect on short-term postoperative HRQoL. In that aspect, major liver surgery is associated with more severe and frequent side effects than minor liver surgery.^4^, ^5^, ^6^

In minor liver resection, the laparoscopic approach has been proven to reduce perioperative complications and the negative impact of surgery on HRQoL, establishing laparoscopy as the current standard for several indications.^5^, ^6^, ^7^, ^8^, ^9^ In major liver resection, laparoscopy may also reduce side-effects and impact on HRQoL, which has not been investigated in a randomised controlled trial yet.

The ORANGE II PLUS trial compared laparoscopic and open hemihepatectomy for benign and malignant indications.^10^ Its primary outcome, time to functional recovery, was significantly reduced in the laparoscopic group. In addition, multiple secondary outcomes were also found to be advantageous to laparoscopy, most importantly a shorter hospital stay, earlier initiation of adjuvant chemotherapy, a smaller decrease in the global health status (as measured by EORTC-QLQ-C30) and better body image and cosmesis compared to open hemihepatectomy group. The initial publication did not provide an in-depth analysis of the separate domains of the HRQoL questionnaires.

We here present a detailed analysis of all relevant domains of HRQoL, including symptoms, reported by patients in the ORANGE II PLUS trial who underwent either laparoscopic or open hemihepatectomy, in order to provide insight into the specific advantages or disadvantages of LH on health-related postoperative performance. As the largest influence on HRQoL is to be expected in the first months after surgery, the primary goal of this study was to assess HRQoL differences over the cumulative period of discharge to 3 months after surgery. Secondarily, the 12-month overall impact on HRQoL was evaluated. Moreover, this study also assessed sex (male/female) differences in body image and cosmesis, and HRQoL differences across various European countries.

## Methods

### Study design and participants

The ORANGE II PLUS trial was a phase 3 randomised controlled trial with a superiority design, conducted in 16 centres specialised in hepatobiliary surgical oncology across six European countries, designed to provide evidence on the merits of laparoscopic versus open hemihepatectomy, with time to functional recovery as the primary outcome.^10^ Eligible patients were adults with an indication for left or right hemihepatectomy, as decided at the local multidisciplinary tumour board meeting, and were eligible to participate if suitable for both a laparoscopic as well as an open approach. Patients had to have a body mass index between 18 and 35 kg/m2 and an American Society of Anaesthesiologists physical status of less than IV. Patients were also required to understand the nature of the study and its obligations and be able to provide written informed consent. To increase recruitment, protocol amendments were submitted and approved to extend inclusion criteria with the following additions: One additional ablation, metastasectomy, or wedge resection on the contralateral side of the liver was permitted, and patients ≥18 years old were eligible. Patients were excluded if they could not fulfil the inclusion criteria, were pregnant or breastfeeding, had previously undergone any form of hepatectomy or had hepatic lesions too close to vascular or biliary structures to be safely operated on laparoscopically. Previous open abdominal surgery or chemotherapy were not considered contraindications for inclusion.

### Ethics approval

Ethical approval of the study protocol was obtained from Maastricht University Medical Centre (METC NL36215.068.11). The study was designed by the authors and is registered at ClinicalTrials.gov (NCT01441856). All patients were given a detailed description of the study including contact information of the researcher at least 1 week prior to inclusion. Written informed consent was obtained from all participating patients. Patients received no financial compensation. The study received no commercial funding. Anonymity and confidentiality were guaranteed for the patients regarding the obtained data. The trial was conducted in accordance with the Declaration of Helsinki and with Good Clinical Practice as defined by the International Conference of Harmonization.

### Randomisation and masking

After written informed consent was obtained, patients were randomly assigned in a 1:1 ratio to either open or laparoscopic hemihepatectomy. Patients were allocated with online randomisation software (ALEA®) using a minimization scheme with hemihepatectomy side and treatment centre to balance treatment arms. For further information regarding randomisation see Supplementary Table S1.

Even though blinding was applied up to four days in the study regarding the main outcome, it was not applicable to the currently secondary analysis since unblinding occurred before the first follow-up questionnaire at discharge and therefore patients were aware of the treatment received upon reporting HRQoL outcomes.

### Interventions

All participating centres were experienced in open and laparoscopic major liver surgery. At the start of their trial accrual, six centres had performed over 40 laparoscopic hemihepatectomies and ten centres had performed between 10 and 40 hemihepatectomies. All centres had a standardised postoperative recovery programme in place. Prior to participation, the trial protocol was approved by each local medical and research ethical committee. The surgical techniques for laparoscopic and open left or right hemihepatectomy were not standardised, so participating surgeons could use their preferred methods for intra-abdominal access, perform liver parenchymal transection, maintain vascular control, and closure of the surgical wound.

Patients completed questionnaires at regular outpatient clinic visits on paper the day prior to surgery, at hospital discharge and at 10 days, 3 months, 6 months, and 1 year after surgery. Subsequently, designated research personnel entered the paper-based data into the online data capturing software (OpenClinica, community version 3.14). In case the patient did not attend the outpatient clinic, the designated research personnel called the patients and entered the data directly into the database while verbally guiding the patient through the questions. In compliance with good clinical practice guidelines, all gathered data were pseudo-anonymised and stored in a secured database for a maximum period of 15 years.

### Outcomes

The European Organization for Research and Treatment of Cancer (EORTC) quality of life core (QLQ-C30) questionnaire and the quality of life liver module (QLQ-LMC21) questionnaire were used to collect HRQoL data.^11^, ^12^, ^13^ The EORTC HRQoL questionnaires have regularly been used in randomised controlled trials involving patients with liver cancer or metastases.^5^^,^^14^, ^15^, ^16^, ^17^, ^18^ Validated translations of the questionnaires were obtained for all 6 included languages in the trial from the EORTC.

The EORTC QLQ-C30 (version 3.0) includes 30 items, which are transformed into a global health status, physical-, role-, emotional-, cognitive-, and social functioning, and 8 symptom scales (fatigue, pain, nausea or vomiting, dyspnoea, insomnia, appetite loss, constipation, and diarrhoea) according to the questionnaires’ standardised scoring procedure. All EORTC QLQ-C30 scale scores range from 0 to 100. Higher scores for a function scale represent a higher level of functioning, whereas higher scores for a symptom scale represent a higher occurrence of symptoms.^11^, ^12^, ^13^ Results on the global health status (GHS) have been published previously.^9^ Data on this outcome will be presented here again for consistency.

The EORTC QLQ-LMC21 (version 1.0) is composed of 13 items translated into 13 symptom scales, each likewise converted to a score ranging from 0 to 100. A higher score for a symptom scale represents a higher occurrence of symptoms.^11^, ^12^, ^13^^,^^19^

The Body Image Questionnaire (BIQ) was used to assess body image and cosmesis and consisted of eight items. Five items are used to evaluate body image and are converted into a score ranging from 5 to 20. Three items are used to evaluate cosmesis after surgery, consisting of two 7-point Likert scales and a 1–10 score, and are converted to a total score ranging from 3 to 24. A high score for body image represents a low satisfaction with one’s appearance, while a high score for the cosmesis represents a high satisfaction with the aesthetics of the scars.^20^^,^^21^

Specific scales were selected for multivariable analysis that were hypothesised to be most distinctive for the assessment of short- and long-term health after open and laparoscopic hemihepatectomy; physical functioning, cognitive functioning, role functioning, social functioning, emotional functioning, pain, fatigue, appetite loss, nutritional problems, peripheral neuropathy, and for further exploration body image and cosmesis.^6^^,^^11^, ^12^, ^13^^,^^17^^,^^22^^,^^23^ The mean results for all other scales in each arm are presented through linear graphs at each timepoint in the Supplementary Figures.

### Sample size

A drop-out rate of 10% and a loss in degrees of freedom for estimating covariate effects (hemihepatectomy side and centre) was anticipated, leading to a total sample size of 250 patients to demonstrate a 2-day reduction in time to functional recovery, i.e., the primary outcome of the trial, with a two-sided 4% level of significance and a power of 80%, assuming a standard deviation (SD) of time to functional recovery of 5 days within both groups. Based on the interim analysis the sample size was extended to 350 patients. Only the primary outcome was assessed during interim analysis and did not influence HRQoL outcomes or analysis. A post hoc sample size analysis was conducted based on global health status to determine to what degree the data is feasible and can be assessed accordingly. Generally, a difference of 10 points on the 100-point QLQ-C30 and QLQ-LMC21 scale between the two groups was considered to be of strong clinical relevance.^24^ The expected standard deviation of this scale is set at 20 points. With 352 patients (177 laparoscopic, and 175 open hemihepatectomy), a two-sided α set at 0.05, a power of 80%, a repeated measures design with 5 follow up time points, and an assumed within-subject correlation of 0.5, the current study should be able to observe a minimally detectable effect size of 2.66. The sample size of the ORANGE II PLUS trial is therefore considered appropriate for this specific analysis.

### Statistical analysis

All procedures followed in this study are in accordance with the 2020 SISAQOL recommendations, the 2013 ISOQOL recommendations, the 2011 CONSORT extension, 2013 CONSORT-PRO extension, and 2017 CONSORT-NPT extension.^25^, ^26^, ^27^, ^28^ Adherence rates were defined as the proportion of valid questionnaires received as compared to the number expected. Expected questionnaires were defined as all questionnaires that could reasonably be obtained, c.q. from all patients that were not deceased or lost-to-follow-up at a given timepoint. Questionnaires of deceased and lost-to-follow-up patients were obtained up until the moment of death or loss-to-follow-up. The adherence rates are presented for each follow-up moment, for the treatment groups separately, using absolute numbers and relative percentage, as recommended by the EORTC and the SISAQOL consortium.^12^^,^^28^ To evaluate adherence between the two study arms at every follow-up moment a Pearson Chi-square test was used. The trial protocol and statistical analysis plan can be found in the Appendix.

In addition to the modified intention-to-treat drop-outs, patients that only completed the baseline questionnaire, but none of the follow-up questionnaires, could not be included in the analysis. A linear mixed regression model was used to compare the scores of each HRQoL domain between treatment arms over a cumulative period of discharge to 3 months after surgery and over the cumulative period of discharge to 12 months after surgery. Mean changes in QoL scores were analysed using a generalised linear mixed model with restricted maximum likelihood (REML) estimation. The model included fixed effects for treatment, participating centre, hemihepatectomy side, age, sex, time point and benign or malignant tumour type, and item-specific baseline scores, and an intercept. An unstructured covariance structure was assumed to model the within-patient errors across repeated measures, allowing variances and covariances between time points to vary freely without assuming independence or constant variance. Parameter estimation was performed using the Newton–Raphson algorithm. Degrees of freedom for the fixed effects were calculated using the residual method. Since this study is a secondary analysis, only effect sizes and 95% confidence intervals were reported. Consequently, there was no allowance for multiplicity, and no correction for multiple testing needed to be applied. All unadjusted, c.q. univariate, outcomes are presented in the Supplementary Appendix. Differences in scores at every follow-up moment between treatment arms were additionally visualised in graphs with individual item means and corresponding confidence intervals at each follow-up moment. Clinical relevance criteria are used to weigh outcomes. Each individual scale of the EORTC-QLQ-C30 is subjected to different clinical relevance criteria. No thresholds for clinical relevance exist for the EORTC-QLQ-LMC21 questionnaire, therefore, we defined a difference of 4 points between arms as clinically relevant. For more detail on clinical relevance criteria see Supplementary Document 1.

As a sensitivity analysis, multiple imputation was applied for all missing data and the linear mixed model was repeated with the 5 pooled imputed data sets as shown in Supplementary Document 1.^28^

A subgroup analysis was performed including only patients that received surgery for cancer, since recurrence of disease and adjuvant chemotherapy could influence these patients as opposed to patient with benign indications. Secondly, the importance of aesthetics and the negative impact of scar can be different between sexes.^21^ Therefore, we performed a subgroup analysis of body image outcomes between males and females in the separate treatment arms. Lastly, to further explore international differences in HRQoL, we performed a subgroup analysis comparing the global health status per country excluding 2 countries due to too small inclusion numbers (n = 6 and n = 13).

All analyses were done in accordance with the recent International Standards for the Analysis of Quality of Life and Patient Reported Data from Clinical trials using IBM SPSS Statistics software version 27.0 (SPSS, Chicago, Illinois, USA) and R statistical computing for Windows version 4.1.0. For data handling procedure we refer to Supplementary Document 1.

### Role of the funding source

All funding sources are non-commercial and had no input in the study design, the collection, analysis, or interpretation of data; nor in the writing of the report, or in the decision to submit the paper for publication.

## Results

### Patients

**Fig. 1:**
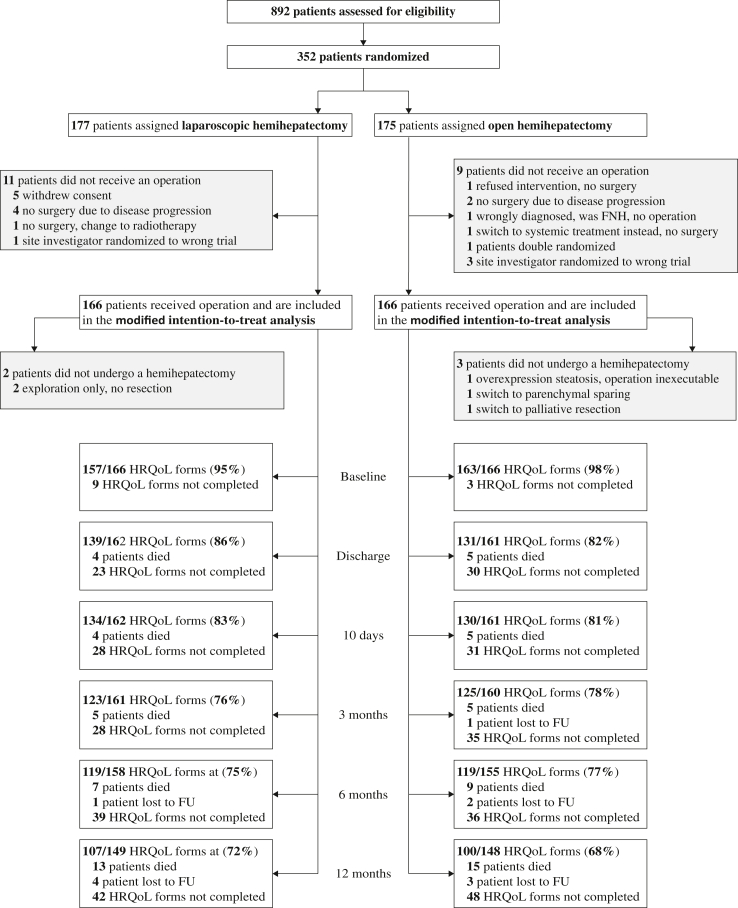
Consort flowchart showing HRQoL adherence in patients undergoing either laparoscopic or open hemihepatectomy in the mITT and PP population.

Between October 2013 and January 2019, a total of 352 patients were allocated to laparoscopic (n = 177) or open hemihepatectomy (n = 175). The average age was 62 years and 41% were female. In total, 85% of patients had cancer, of whom 59% had colorectal cancer liver metastases. No significant differences were seen in any of the demographic or clinical characteristics between the two arms of the trial (see Table 1). Twenty patients dropped out before surgery and were excluded from the modified intention-to-treat analysis (Fig. 1). Hence, the HRQoL analysis was conducted in 332 patients, 166 in the LH group and 166 in the OH group. The additional per-protocol analysis excluded five patients who underwent surgery, but not hemihepatectomy. However, for ethical reasons, these five patients did not complete HRQoL questionnaires following surgery and are therefore not included in the analysis. As a result, the mITT and per-protocol groups consist of the same patients for the purposes of this analysis.

**Table 1:** Baseline demographics of patients undergoing laparoscopic or open hemihepatectomy in the mITT population.

Characteristic	Laparoscopic hemihepatectomy (n = 166)	Open hemihepatectomy (n = 166)
Sex		
Male	99 (60)	96 (58)
Female	67 (40)	70 (42)
Age, years	62 ± 14	63 ± 13
BMI^a^	26 (23–29)	25.0 (22–28)
ASA classification		
I: healthy	13 (8)	19 (11)
II: mild systemic disease	93 (56)	91 (55)
III: severe systemic disease	52 (31)	52 (31)
ECOG performance status score		
0: asymptomatic, normal activity	121 (73)	123 (74)
1: symptomatic, normal activity	36 (22)	40 (24)
2: symptomatic, <50% bedridden	4 (2)	1 (1)
3: symptomatic, >50% bedridden	1 (1)	0
4: 100% bedridden		
Charlson comorbidity index	6 ± 3	6 ± 3
Previous abdominal surgery	87 (52)	92 (55)
Preoperative portal vein embolisation	16 (10)	9 (5)
Preoperative chemotherapy	53/136 (39)	61/145 (42)
Radiological diagnosis		
Benign	30 (18)	20 (12)
Haemangioma	6 (4)	6 (4)
Adenoma	5 (3)	0
Follicular nodular hyperplasia	0	2 (1)
Other benign	15 (9)	12 (7)
Malignant	136 (82)	145 (87)
Colorectal metastasis	89 (54)	79 (48)
Hepatocellular carcinoma	22 (13)	25 (15)
Cholangiocarcinoma	17 (10)	30 (18)
Other malignant	11 (7)	12 (7)
Hemihepatectomy side		
Left	61 (37)	58 (35)
Right	105 (63)	108 (65)
Additional contralateral surgery		
Wedge resection	18 (10)	18 (10)
Ablation	6 (3)	3 (2)
Ablation and wedge resection	2 (1)	2 (1)
Country^b^		
Netherlands	20 (12)	20 (12)
Germany	4 (2)	3 (2)
Italy	44 (27)	41 (25)
Belgium	36 (22)	36 (22)
United Kingdom	56 (34)	59 (36)
Norway	6 (4)	7 (4)

Data are n (%), median (IQR) or mean ± SD.

LH, laparoscopic hemihepatectomy; OH, open hemihepatectomy; ASA, American Society of Anaesthesiologists; BMI, body mass index; ECOG, Eastern Cooperative Oncology Group.

a The body mass index is the weight in kilograms divided by the square of the height in meters.

b For centre specific see Supplementary Table S9.

### Baseline scores and HRQoL adherence rates

No large differences were observed at baseline for any of the HRQoL outcomes (Table 2). Collectively, the 332 patients completed >80% of the surveys between baseline and 12 month follow-up, i.e., 1546 questionnaires were available. Twenty-eight patients (8%) died within the first year after surgery and 7 patients (2%) were lost to follow up, both were equally divided between arms. For the maximum expected collectable questionnaires per timepoint see Table 3. For the actual adherence rates see Fig. 1. No significant differences in adherence were observed between the two groups at any time point. For percentage completed questionnaires per timepoint see Fig. 1.

**Table 2:** Health-related quality of life outcomes at baseline of patients undergoing laparoscopic or open hemihepatectomy in the mITT population.

HRQOL outcome	LH (n = 157)	OH (n = 163)
**QLQ-C30—global health status**		
Global health status	75 (67–83)	75 (67–83)
**QLQ-C30–functional scales**		
Physical functioning	93 (87–100)	100 (80–100)
Role functioning	100 (67–100)	100 (83–100)
Emotional functioning	83 (67–100)	83 (67–100)
Cognitive functioning	100 (83–100)	100 (83–100)
Social functioning	100 (67–100)	100 (67–100)
**QLQ-C30–symptom scales**		
Fatigue^a^	11 (0–33)	11.1 (0.0 to 33.3)
Nausea and vomiting	0	0
Pain^a^	0 (0–17)	0 (0–17)
Dyspnoea	0	0
Insomnia	0 (0–33)	0 (0–33)
Appetite loss	0	0
Constipation	0	0
Diarrhoea	0	0
Financial problems	0	0
**QLQ-LMC21–symptom scales**		
Nutritional problems	0 (0–17)	0 (0–4)
Fatigue^a^	11 (0–33)	11 (0–33)
Pain^a^	0 (0–22)	0 (0–22)
Emotional problems	25 (8–42)	25 (8–50)
Weight loss	0	0
Taste	0	0
Dry mouth	0 (0–33)	0 (0–33)
Sore mouth	0	0
Peripheral neuropathy	0 (0–33)	0 (0–33)
Jaundice	0	0
Contact with friends	0	0
Talking about feelings	0	0 (0–33)
Sex life	0 (0–33)	0

Data are median (IQR).

LH, laparoscopic hemihepatectomy; OH, open hemihepatectomy.

a The domains fatigue and pain occur in both the EORTC-QLQ-C30 and the EORTC QLQ-LMC21. Median baseline outcomes with multiple imputation can be found in Supplementary Table S8.

**Table 3:** Maximum expected collectable questionnaires at different timepoints for health-related quality of life of patients undergoing laparoscopic or open hemihepatectomy in the mITT population.

Timepoint	Death^b^		Lost to follow-up		Expected questionnaires	
	LH (n = 166)	OH (n = 166)	LH (n = 166)	OH (n = 166)	LH^a^	OH^a^
Baseline	0	0	0	0	166	166
Discharge	4	5	0	0	162	161
10-day FU	4	5	0	0	162	161
3-month FU	5	5	0	1	161	160
6-month FU	7	9	1	2	158	155
12-month FU	13	15	4	3	149	148

Data are n.

a Please note that the numbers presented in these columns do not presented the actual collected questionnaires, but rather the maximum possible number of questionnaires to be collected at that specific timepoint.

b An overview of 90-day mortality is provided in Supplementary Table S7.

### EORTC-QLQ-C30 selected outcomes

**Fig. 2:**
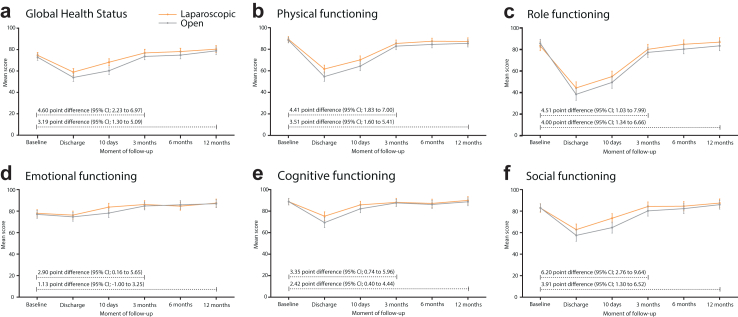
EORTC-QLQ-C30 global health status and functioning scales over the period of baseline to 12 months after either laparoscopic or open hemihepatectomy in the mITT population. Legend: Visualisation of mean health-related quality of life EORTC-QLQ-C30 global health status and functioning scores at each follow up moment after either laparoscopic or open surgery. a: Global health status, b: Physical functioning, c: Role functioning, d: Emotional functioning, e: Cognitive functioning, f: Social functioning.

Cumulatively, over the first postoperative period from discharge to 3 months, a clinically relevant difference was observed in physical functioning (difference 4.20 points; 95% CI 1.07–7.34), in cognitive functioning (difference 3.11 points; 95% CI 0.17–6.40), and in social functioning with (5.95 points; 95% CI 1.65–10.26), all in favour of the laparoscopic approach. Cumulatively, over the period of discharge to 12 months after surgery, clinically relevant differences were observed for physical functioning (3.83 points; 95% CI 1.14–6.54) and social functioning (4.23 points; 95% CI 0.36–8.10). However, between 3 and 12 months, the HRQoL difference attenuated, as visualised in Fig. 2. Differences in role functioning (4.66 points; 95% CI 0.64–8.68) were not deemed clinically relevant (Table 4).

**Table 4:** Cumulative outcome differences of Health-related quality of life over the period of discharge to 3 months and to 12 months after either laparoscopic or open hemihepatectomy including multiple imputation of missing values model in the mITT population.

HRQoL outcome	Adjusted difference over 3 months^a^ (mean [95% CI])	Adjusted difference over 12 months^a^ (mean [95% CI])	Adjusted difference over 12 months, multiple imputation (mean [95% CI])	Clinical relevance range^b^
**QLQ-C30—global health status**				
LH	4.63 (1.50–7.75)	3.23 (0.48–5.98)	2.49 (−0.06 to 5.03)	−5≤ or ≥5
OH	Reference	Reference	Reference	
**QLQ-C30–functional scales**				
Physical functioning				
LH	4.20 (1.07–7.34)	3.83 (1.14–6.54)	2.59 (0.02–5.21)	−5≤ or ≥2
OH	Reference	Reference	Reference	
Role functioning				
LH	4.66 (0.64–8.68)	4.41 (1.07–7.75)	3.61 (0.45–6.77)	−7≤ or ≥6
OH	Reference	Reference	Reference	
Emotional functioning				
LH	2.33 (−1.30 to 5.96)	0.98 (−2.39 to 4.35)	0.51 (−2.51 to 3.52)	−3≤ or ≥6
OH	Reference	Reference	Reference	
Cognitive functioning				
LH	3.11 (0.17–6.40)	2.60 (0.38–5.57)	2.33 (−0.39 to 5.05)	−1≤ or ≥3
OH	Reference	Reference	Reference	
Social functioning				
LH	5.95 (1.65–10.26)	4.23 (0.36–8.10)	2.54 (−0.78 to 5.85)	−6≤ or ≥3
OH	Reference	Reference	Reference	
**QLQ-C30–symptom scales**				
Fatigue				
LH	−4.54 (−8.31 to −0.76)	−2.90 (−6.32 to 0.52)	−2.55 (−5.97 to 0.86)	−5≤ or ≥4
OH	Reference	Reference	Reference	
Pain				
LH	−6.41 (−10.01 to −2.82)	−5.08 (−8.21 to −1.95)	−3.90 (−6.93 to −0.87)	−3≤ or ≥5
OH	Reference	Reference	Reference	
Appetite loss				
LH	−7.29 (−11.59 to −2.99)	−4.36 (−7.89 to −0.82)	−2.82 (−5.77 to −0.12)	−2≤ or ≥7
OH	Reference	Reference	Reference	
**QLQ-LMC21–symptom scales**				
Nutritional problems				
LH	−4.37 (−7.96 to −0.78)	−3.11 (−6.09 to −0.14)	−1.85 (−4.41 to 0.71)	Na
OH	Reference	Reference	Reference	
Peripheral neuropathy				
LH	1.70 (−1.94 to 5.35)	1.32 (−2.10 to 4.73)	−0.40 (−3.81 to 3.02)	Na
OH	Reference	Reference	Reference	
**Body image & Cosmesis**				
Body image^c^				
LH	−1.06 (−1.63 to −0.49)	−0.85 (−1.32 to −0.32)	Na	Na
OH	Reference	Reference		
Cosmesis^d^				
LH	2.29 (1.49–3.08)	2.23 (1.47–2.98)	Na	Na
OH	Reference	Reference		

a Results adjusted for sex, age, hemihepatectomy side, benign/malignant tumour type, treatment centre, and baseline differences. In all analyses, the open group is used as reference group.

b Clinical relevance based on Cocks et al. (Euro. J. Cancer, 2012).

c On a range of 5–20.

d On a scale of 3–24.

**Fig. 3:**
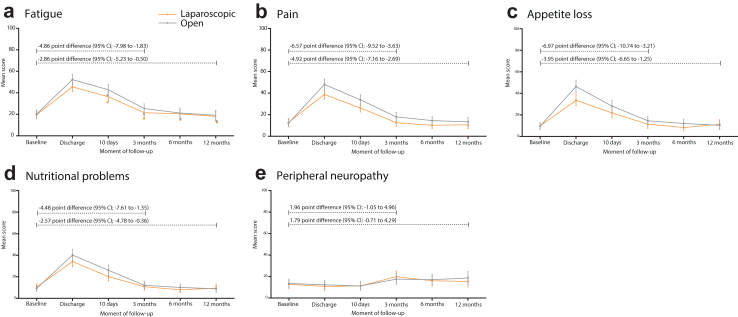
EORTC-QLQ-C30 symptom scales over the period of baseline to 12 months after either laparoscopic or open hemihepatectomy in the mITT population. Legend: Visualisation of mean health-related quality of life EORTC-QLQ-C30 and -QLQ-LMC21 selected symptom scores at each follow up moment after either laparoscopic or open surgery. a: Fatigue, b: Pain, c: Appetite loss, d: Nutritional problems, e: Peripheral neuropathy.

**Supplementary Fig. S1:**
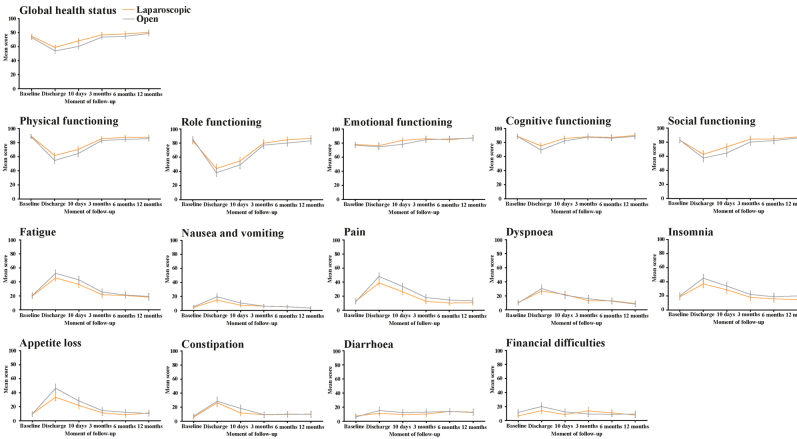


Cumulatively, over the period from discharge to 3 months after surgery, a clinically relevant overall difference could be seen for the domains ‘pain’ (−6.41 points, 95% CI −10.01 to −2.82), and ‘appetite loss’ (−7.29 points, 95% CI −11.59 to −2.99), both in favour of the laparoscopic approach (Table 4). Similar clinically relevant, but slightly attenuated, differences were reported over the cumulative period from discharge to 12 months after surgery. The difference in the domain ‘fatigue’ was not clinically relevant (−4.54 points, 95% CI −8.31 to 0.76) (Table 4, Fig. 3). The mean differences over time for not-selected symptom scales are visualised in Supplementary Fig. S1.

As previously presented, patients assigned to laparoscopic surgery reported better results cumulatively over discharge to 12 months in the global health status domain compared to those assigned to open hemihepatectomy (3.23 points, 95% CI 0.48–5.98) (Table 4, Fig. 2).^10^ However, this difference is too small to be considered clinically relevant. The difference between treatment arms was largest in the first weeks after surgery with a clinically relevant difference (≥5 points) at 10 day follow-up (Supplementary Table S2).

### EORTC-QLQ-LMC21 selected outcomes

**Supplementary Fig. S2:**
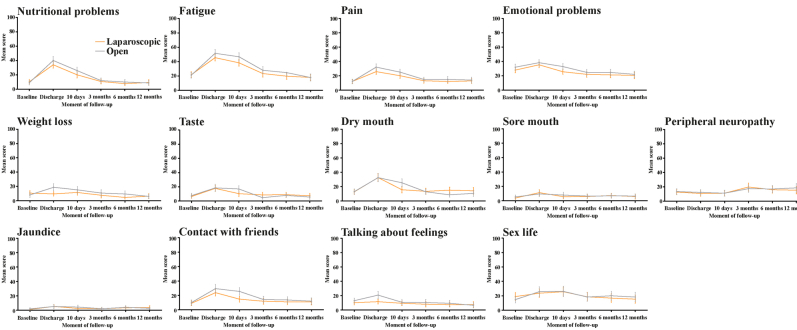


Evaluation of the selected scales of the EORTC QLQ-LMC21 revealed an overall clinically relevant difference (≥4 points) in nutritional problems of −4.37 points (95% CI −7.96 to −0.78), favouring the laparoscopic approach. The difference was also apparent cumulatively over the period of discharge to 12 months after surgery, however it was no longer considered clinically relevant (−3.11 point; 95% CI −6.09 to −0.14). The difference in peripheral neuropathy was not clinically relevantly cumulatively over the period of either discharge to 3 or to 12 months after surgery (Table 4, Fig. 3). The mean differences over time for not-selected symptom scales are visualised in Supplementary Fig. S2.

### Sensitivity analysis

Multiple imputation of all missing data was performed. We observed that after imputation, 12 month differences were slightly attenuated, but no major differences were identified in the sensitivity analysis compared to the main analysis (Supplementary Table S3). Social functioning, however, was no longer clinically relevant. All other previous clinically relevant outcomes remained similar.

### Subgroup analyses

The subgroup analysis of sex (male/female) differences in body image and cosmesis showed that women reported significantly larger differences in both body image scores and cosmesis scores compared to men (Supplementary Table S3). When excluding patients operated for a benign indications, no differences in clinical relevance of outcomes were observed (Supplementary Table S4). Furthermore, we found no geographical differences regarding overall HRQoL outcomes (Supplementary Table S5).

## Discussion

We present a detailed analysis of HRQoL outcomes up to 12 months after surgery from the randomised, multicentre ORANGE II PLUS trial. Over the period of discharge to 3 months postoperatively, patients assigned to laparoscopic hemihepatectomy reported clinically relevant improvements in physical, cognitive and social functioning compared to those assigned to open hemihepatectomy. Over the period of discharge to 12 months after surgery, clinically relevant, but slightly attenuated, differences of physical functioning and social functioning were also reported by the patients. Previous findings showed a shorter time to functional recovery with laparoscopic hemihepatectomy.^10^ This aligns with the current findings of patients reporting less bodily pain, appetite loss and nutritional problems after laparoscopic hemihepatectomy emphasizing the reduced physical impact of the laparoscopic approach. Subsequently, the accelerated recovery following laparoscopy likely enhances postoperative fitness and confidence, thereby reducing impediments in their social functioning.

All scales were repeatedly measured over 12 months after resection. Recovery typically improves gradually, with the largest gains in the first weeks. In the current study this is also the case, as visualised in Figs. 2 and 3. Previous reports suggest that HRQoL outcomes return to baseline after 6 months and remain stable up to 12 months.^8^^,^^23^ Our study confirms this, with 75% of outcomes reverting to baseline at 6 months. Thus, the improvement in HRQoL following laparoscopic hemihepatectomy is most pronounced in the short-term, without long-term negative repercussions. As is common for reports on HRQoL, higher numbers of missing data were seen at each consecutive follow up moment, with most missing cases at 12 months after surgery. However, the distribution of missing data remained similar between arms throughout the study’s follow-up, as expected in a randomised trial, as shown in Fig. 1. Therefore, no intergroup bias is introduced by the increased number of missing questionnaires. The reason for the increase in missing cases is unknown, but is likely to be related to patients either failing to attend the outpatient appointment or being unwilling to complete the questionnaire. The diminishing differences at 6 and 12 months after surgery become more apparent when missing data was imputed which resulted in slightly attenuated overall differences.

Enhanced recovery after surgery (ERAS) programmes have significantly improved recovery after both open and laparoscopic procedures.^29^ Laparoscopy has been consistently shown to reduce postoperative length of stay and complication rates, also for major hepatectomy. It has therefore been integrated in the ERAS guidelines, advocated to be pursued in trained teams when clinically appropriate for both benign and malignant disease.^30^ In the current trial, the ERAS-components were equally applied to the open an laparoscopic groups and this resulted in very short recovery times and complications in either group. Nonetheless, compared to open hemihepatectomy, patients who underwent laparoscopic hemihepatectomy were able to recover even faster and also with clinically relevant advantage in several HRQoL outcomes. This highlights the pivotal role of laparoscopy within the ERAS guidelines.

Patient satisfaction with postoperative bodily appearance and scars significantly favoured laparoscopic hemihepatectomy, emphasising the positive impact of smaller scars. Sex-specific subgroup analysis revealed more substantial differences in body image and cosmesis scores among women than men, suggesting that a larger scar may have a more negative effect on women’s quality of life.

To our knowledge, no other trials have compared HRQoL outcomes between open and laparoscopic major liver surgery in a prospective, randomised setting. Several studies have reported on HRQoL after liver resection, most of which are summarised in a recent systematic review and meta-analysis.^6^ This review predominantly included retrospective and observational studies of both minor and major liver surgery and showed similar results to the current trial. However, these studies often compare HRQoL between benign and malignant indications, with no exclusive analysis of major resections.^18^^,^^31^

The OSLO-COMET randomised controlled trial comparing laparoscopic and open minor liver surgery found significantly less bodily pain and significantly better vitality, role physical and social functioning one month after laparoscopic surgery compared to the open approach. Most outcomes had returned to baseline levels after four months.^8^ These outcomes are in line with our current trial, with the addition of less nutritional problems, which can be the case because a different measuring tool for evaluating HRQoL was used.

In our study, although the number of benign cases was equally divided between treatment arms, the low number precludes adjustments for differences in surgical indication. The systematic review underscores the limited availability of HRQoL data for laparoscopic liver resections, which hinders valid comparisons with open resections, highlighting the importance of our findings.^6^

In 2004, Korolija et al. evaluated HRQoL in laparoscopic versus open surgery across various surgical fields, forming the evidence-based HRQoL guidelines of the European Association for Endoscopic Surgery.^32^ Although liver resections were not specifically included, improved postoperative HRQoL outcomes were found for all surgical indications after laparoscopy.^32^ Later, Rees et al. (2012 and 2014) reported decreases in global health and functional health up to 3 months after open liver surgery, with recovery by 6 months, and stability until 12 months.^23^ Our study observed a similar trend in functional health, but global health returned to baseline levels in both arms at 3 months, likely due to improved perioperative care and enhanced recovery programs.^5^^,^^29^ Giuliani et al. (2014) compared HRQoL between open and laparoscopic liver resections for benign lesions, finding better physical functioning and less bodily pain 6 months after laparoscopic surgery, consistent with our results.^33^

The international multicentre collaboration in this randomised trial yielded a high sample size providing abundant HRQoL data with global relevance. While cultural variations in quality of life perceptions are a concern, the use of EORTC-validated questionnaires and a minimisation scheme that stratified for hemihepatectomy side and centre ensured international comparability.^34^ Additionally, the implementation of ERAS programmes at all participating centres further attenuated cultural bias in clinical care, as is confirmed by the subgroup analysis per country. An inevitable limitation of this study was the inability to blind patients for the applied surgical approach when questionnaires were administered. Furthermore, while the guidelines used to determine clinical relevance are well-supported, they are based on different types of diseases and may therefore underestimate the significance of the current findings, particularly in the context of liver disease. Additionally, as a secondary analysis, the current research was not primarily designed to address HRQoL, so results should be interpreted with caution. Future studies focusing specifically on HRQoL after major liver surgery are needed to provide definitive conclusions.

Ensuring quality of life during and after treatment is essential for patients surviving liver cancer. Notably, 31% of patients in the ORANGE II PLUS trial died or had disease recurrence within the first year post-surgery (Supplementary Table S6). For these patients, the benefits of laparoscopic surgery in minimizing surgery-related limitations are especially significant. Prioritising the surgical modality that provides the highest quality of life during this limited timeframe seems imperative.

### Conclusion

This in-depth secondary analysis of the ORANGE II PLUS randomised trial suggests that patient reported HRQoL was clinically superior in physical functioning, cognitive functioning, and social functioning, occurrence of bodily pain, appetite loss and nutritional problems over the period of discharge to 3 months after laparoscopic hemihepatectomy compared to open hemihepatectomy.

## Contributors

RSF, LAA, MAH, CHCD, GJPVB, JNP, and RVD prepared the first draft of the protocol. RSF, LAA, MAH, CHCD, GJPVB, JNP, and RVD conceptualised the study design. BO, RSF, LAA, MAH, SP, LB, ZE, GJPVB, JNP, and RVD wrote the statistical analysis plan. LB was the lead statistician with BO providing additional data management and statistical support, and all had access to all the data. BO, RSF, AAF, LAA, SP, ZE, LB, JNP, and RVD had access to and verified the underlying study data. BO, RSF, AAF, LAA, SP, LB, JNP, and RVD participated in data analysis and interpretation. All authors participated in patient enrolment, trial execution and management, and critically reviewed the report and approved the final version before submission. All authors had full access to all the data in the study and had final responsibility for the decision to submit for publication.

## Data sharing statement

Data collected for the study, including de-identified individual participant data and a data dictionary defining each field in the set, can be made available to others on reasonable request and after signing appropriate data sharing agreements after all following studies on this main paper by the research team have been concluded. Please send data access requests to r.van.dam@mumc.nl. Such requests must be approved by the respective ethics boards and appropriate data custodians.

## Declaration of interests

We declare no competing interests.

## Appendix A Supplementary data

ORANGE-II-PLUS final Statistical Analysis Plan

ORANGE-II-PLUS final study protocol

ORANGE II PLUS Collaborators list of Study Group

ORANGE II PLUS HRQoL Supplementary Material with caption
