# Supplementary material for: Health-related quality of life in patients undergoing laparoscopic versus open hemihepatectomy: a secondary analysis of the ORANGE II PLUS randomised controlled, phase 3, superiority trial
Source: Lancet Reg Health Eur. 2025 May 19;54:101311. doi: 10.1016/j.lanepe.2025.101311 (PMC12148447; doi:10.1016/j.lanepe.2025.101311)
Supplement: ORANGE-II-PLUS final Statistical Analysis Plan [file mmc1.pdf]

ORANGE II PLUS & ORANGE SEGMENTS

CLINICAL TRIAL STATISTICAL ANALYSIS PLAN

LIST OF ABBREVIATIONS

ALT	Alanine aminotransferase
ASA	American society of Anaesthesiologists
AST	Aspartate aminotransferase
BIQ	Body image questionnaire
CCI	Comprehensive complication index
CI	Confidence interval
FR	Time to functional recovery
HRQoL	Health-related Quality of Life
INR	International normalised ratio
IQR	Interquartile range
ITT	Intention-to-treat analysis
KEMTA	Clinical Epidemiology & Medical Technology Assessment
LOS	Length of hospital stay
PP	Per-protocol analysis
QALY	Quality adjusted life year
QoL	Quality of life
SAP	Statistical analysis plan
SD	Standard deviation
SMD	Standardised mean difference
TB	Total bilirubin
VAS	Visual analogue scale
WHO	World Health Organisation

TABLE OF CONTENTS

SECTION 1 INTRODUCTION 4
1.1 PURPOSE OF SAP 4
1.2 STUDY OVERVIEW 4
1.2.1 Titles 4
1.2.2 Planned Patient Population 4
1.2.3 Inclusion criteria 4
1.2.4 Exclusion criteria 5
1.2.5 Objectives / Aims from protocol 5
1.3 DEFINITION OF OUTCOMES 7
1.3.1 Primary outcome 7
1.3.2 Secondary outcomes 11
1.3.3 Definition of safety outcomes 39
1.4 SUMMARY OF PROTOCOL AMENDMENTS 39
SECTION 2 DESIGN ISSUES 41
2.1 DATA COLLECTION AND FOLLOW-UP 41
2.2 STUDY DESIGN 42
2.3 TREATMENT ALLOCATION/ RANDOMISATION 42
2.4 BLINDING 43
2.5 STUDY POWER AND SAMPLE SIZE 43
2.6 INTERIM ANALYSES AND STOPPING GUIDANCE 45
2.7 TIMING OF ANALYSES 45
2.8 PROTOCOL DEVIATIONS 46
SECTION 3 STATISTICAL ANALYSES 47
3.1 DEFINITION OF ANALYSIS POPULATIONS 47
3.1.1 Intention-to-treat analyses 47
3.1.2 Per-protocol analyses 47
3.2 ANALYSIS SOFTWARE 48
3.3 HYPOTHESIS TESTING 48
3.3.1 Primary outcome 48
3.3.2 Secondary outcomes 49
3.4 MULTIVARIABLE ANALYSES 49
3.4.1 Continuous outcome variables 49
3.4.2 Dichotomous or categorical outcome variables 49
3.4.3 Time-to-event outcome variables 49
3.4.4 Clustered data 49
3.5 COVARIATE ADJUSTMENT 50
3.5.1 Covariate adjustments for ORANGE II PLUS 50
3.5.2 Covariate adjustment for ORANGE SEGMENTS 50
3.6 INTERACTION 50
3.6.1 Interaction terms ORANGE II PLUS 50
3.6.2 Interaction terms ORANGE SEGMENTS 50
3.7 METHODS FOR HANDLING WITHDRAWALS, MISSING DATA AND OUTLIERS 50
3.7.1 Withdrawals 51
3.7.2 Missing data 51
3.8 CHECKING ASSUMPTIONS AND DATA TRANSFORMATIONS 51
3.8.1 Normality 51
3.8.2 Proportional hazards 51
3.9 HANDLING MULTI-CENTRE/CLUSTERED DATA 51
3.9.1 Adjustments for clustering 51
3.10 SUBGROUP ANALYSES 51
3.10.1 Interaction subgroup analyses 51
SECTION 4 DISPOSITION OF PATIENTS AND FOLLOW-UP INFORMATION 53

SECTION 5 DEMOGRAPHIC AND BASELINE CHARACTERISTICS 54
SECTION 6 PRIMARY OUTCOME ANALYSIS 57
SECTION 7 SECONDARY OUTCOME ANALYSES 58
7.1 HOSPITAL LENGTH OF STAY 58
7.2 INTRAOPERATIVE BLOOD LOSS 58
7.3 OPERATION TIME 58
7.4 CONVERSIONS 58
7.5 UNFAVOURABLE INTRAOPERATIVE INCIDENTS 58
7.6 POSTOPERATIVE MORBIDITY – ACCORDION 59
7.7 POSTOPERATIVE MORBIDITY – CLAVIEN-DINDO 59
7.8 POSTOPERATIVE MORBIDITY – COMPREHENSIVE COMPLICATION INDEX 59
7.9 POSTOPERATIVE MORBIDITY – COMPOSITE ENDPOINT 59
7.10 90-DAY MORTALITY 59
7.11 READMISSION 59
7.12 RESECTION MARGIN 60
7.13 TIME TO ADJUVANT CHEMOTHERAPY INITIATION 60
7.14 QUALITY OF LIFE – EORTC QLQ-C30 – GLOBAL HEALTH SCORE 60
7.15 QUALITY OF LIFE – EORTC QLQ-C30 – FUNCTIONAL SCORE 60
7.16 QUALITY OF LIFE – EORTC QLQ-LMC21 – SYMPTOMS SCORE 60
7.17 BODY IMAGE SCORE 60
7.18 COSMESIS SCORE 61
7.19 DELAY IN DISCHARGE AFTER FUNCTIONAL RECOVERY 61
7.20 12-MONTH INCIDENCE OF INCISIONAL HERNIA 61
7.21 DIRECT AND INDIRECT COSTS 61
7.22 OVERALL 5-YEAR SURVIVAL 61
SECTION 8 SUBGROUP ANALYSES 65
ADDITIONAL SUBGROUP ANALYSES 65
8.1 NORMAL VERSUS OVERWEIGHT VERSUS OBESITY BODY MASS INDEX 65
8.2 MALIGNANT LIVER TUMOURS 65
8.3 PREOPERATIVE PORTAL VENOUS EMBOLISATION VERSUS NO PREOPERATIVE VENOUS EMBOLISATION 66
8.4 PREOPERATIVE CHEMOTHERAPY VERSUS NO PREOPERATIVE CHEMOTHERAPY 66
8.5 PREVIOUS ABDOMINAL SURGERY VERSUS NO PREVIOUS ABDOMINAL SURGERY 66
8.6 WORLD HEALTH ORGANISATION PERFORMANCE STATUS 66
8.7 AMERICAN SOCIETY OF ANAESTHESIOLOGISTS PHYSICAL STATUS CLASSIFICATION SYSTEM 66
8.8 CONVERSIONS 66
8.9 UNFAVOURABLE INTRAOPERATIVE INCIDENTS 66
8.10 INTRAOPERATIVE BLOOD LOSS 66
8.11 INTRAOPERATIVE INOTROPIC MEDICATION 67
8.12 POSTOPERATIVE COMPLICATIONS 67
8.13 COMPOSITE ENDPOINT OF MORBIDITY AFTER LIVER SURGERY COMPLICATIONS 67
8.14 COMPREHENSIVE COMPLICATION INDEX 67
8.15 QUALITY OF LIVER PARENCHYMA 67
META-ANALYSES 68
SECTION 9 SAFETY OUTCOMES 69
9.1 SAFETY OUTCOMES AT INTERIM 69
9.2 SAFETY OUTCOMES AT END OF THE TRIAL 69
SECTION 10 REFERENCES 72

Introduction

1.1 Purpose of SAP

The goal of this statistical analysis plan (SAP) is to outline the statistical analyses for the ORANGE II PLUS and ORANGE SEGMENTS trials. This way an objective and transparent set of analyses is provided beforehand. No data analyses are performed before approval of this SAP by the principal investigators, coordinating investigators and trial statistician. The Data and Safety Monitoring Board approved the statistical analysis strategy for the ORANGE II PLUS primary endpoint on November 8, 2018. Any additional or adjusted analyses are reported including rationalisation. The SAP has been based on the trial protocol version 12, dated March 30, 2017.

Data and Safety Monitoring Board	
**Function**	**Name**
**Chair and surgical specialist**<br>Amsterdam University Medical Centers<br>Location Academic Medical Center	Prof. M.A. Boermeester
**Surgical specialist**<br>University Medical Center Utrecht	Prof. I.H.M Borel Rinkes
**Methodologist**<br>Maastricht University Medical Center+	Prof. M.H. Prins

NOTE - The ORANGE II PLUS trial was originally designed to compare open with laparoscopic hemihepatectomies. During the trial, the protocol was amended to include two additional arms comparing open with laparoscopic parenchymal preserving postero-superior liver segment resections. These two arms focus on an independent and different patient population that does not conflict with the former and has been abbreviated the ORANGE SEGMENTS trial. Therefore, while there is one protocol entitled ORANGE II PLUS, the document essentially provides an identical design for both trials. This SAP is in line with the trial protocol, providing the statistical analysis plans for both the ORANGE II PLUS and the ORANGE SEGMENTS. The ORANGE II PLUS and ORANGE SEGMENTS trials are to be published separately.

1.2 Study overview

1.2.1 Titles

1. The ORANGE II PLUS - trial: an international multicentre randomised controlled trial of open versus laparoscopic hemihepatectomies.
   Clinicaltrials.gov identifier: NCT01441856.

2. The ORANGE SEGMENTS - trial: an international multicentre randomised controlled trial of open versus laparoscopic parenchymal preserving postero-superior liver segment resections. Clinicaltrials.gov identifier: NCT03270917.

1.2.2 Planned Patient Population

All patients - 18 years and older - with anatomic benign and/or malignant lesions requiring a right or left hemihepatectomy (with or without the need for one additional hepatic wedge resection or metastasectomy), or patients in need of a parenchymal sparing resection of postero-superior liver segments (involving one or two of segments 4a, 6/7, 7, 8) that the multidisciplinary team feel is technically feasible and safe by either the laparoscopic or open approach.

1.2.3 Inclusion criteria

The trial eligibility criteria are stated below. Patients who were screened and excluded based on the entry criteria are registered in screening logs.

Inclusion criteria ORANGE II PLUS

- Patients requiring left or right hemihepatectomy, with or without the need for one additional hepatic wedge resection or metastasectomy for accepted indications.
- Men and non-pregnant, non-lactating women between age 18 years and older.
- BMI between 18-35 kg/m^2.
- Patients with ASA physical status I, II or III.
- Able to understand the nature of the study and what will be required.

Inclusion criteria ORANGE SEGMENTS

- Patients requiring a parenchymal sparing liver resection (including wedge resections and full segmentectomies) involving one or two of segments 4a, 7, 8 for accepted indications. A segment 6/7 resection would also be eligible.
- Men and non-pregnant, non-lactating women between age 18 years and older.
- BMI between 18-35 kg/m^2.
- Patients with ASA physical status I, II or III.
- Able to understand the nature of the study and what will be required.

1.2.4 Exclusion criteria

Exclusion criteria ORANGE II PLUS

- Patients requiring another resection than left or right hemihepatectomy, with or without the need for one additional hepatic wedge resection or metastasectomy.
- Inability to give written informed consent.
- Patients with hepatic lesion(s), that are located with insufficient margin from vascular or biliary structures to be operated laparoscopically.
- Patients with ASA physical status IV or V.
- Repeat hepatectomy.

Exclusion criteria ORANGE SEGMENTS

- Patients requiring another resection than l a parenchymal sparing resection involving one or two of segments 4a, 7, 8.
- Inability to give written informed consent.
- Patients requiring parenchymal sparing liver resection involving segment 1. This is due to the high level of technical difficulty.
- Patients with hepatic lesion(s), that are located with insufficient margin from vascular or biliary structures to be operated laparoscopically.
- Patients with ASA physical status IV or V.
- Repeat hepatectomy.

1.2.5 Objectives / Aims from protocol

The ORANGE II PLUS and ORANGE SEGMENTS trials are both powered as superiority trials; laparoscopic liver resection is hypothesised to perform better in terms of time to functional recovery compared with open liver resection. Test results are two-sided as they may be either better or worse for laparoscopic liver resection in comparison with open liver resection.

ORANGE II PLUS – primary objective

To compare laparoscopic liver resection with open liver resection, for accepted indications and within an enhanced recovery programme in terms of time to functional recovery in patients undergoing either left or right hemihepatectomy (with or without the need for one additional hepatic wedge resection or metastasectomy).

<u>Primary objective – null hypothesis (H_0)</u>

There is no difference in time to functional recovery between patients undergoing open or laparoscopic hemihepatectomy (with or without the need for one additional hepatic wedge resection or metastasectomy).

Primary objective - alternative hypothesis (H_1)
There is a difference in time to functional recovery between patients undergoing open or laparoscopic hemihepatectomy (with or without the need for one additional hepatic wedge resection or metastasectomy).

ORANGE II PLUS – secondary objectives
To compare laparoscopic liver resection with open liver resection, for accepted indications and within an enhanced recovery programme in terms of hospital length of stay, intraoperative blood loss, operation time, resection margin, time to adjuvant chemotherapy initiation, readmission percentage, (liver-specific) morbidity, quality of life, body image, reasons for delay in discharge after functional recovery, long-term incidence of incisional hernias, hospital and societal costs during one year and overall five-year survival in patients undergoing either left or right hemihepatectomy (with or without the need for one additional hepatic wedge resection or metastasectomy).

Secondary objectives – null hypotheses (H_0)
There is no difference between patients undergoing open or laparoscopic hemihepatectomy (with or without the need for one additional hepatic wedge resection or metastasectomy) in hospital length of stay, intraoperative blood loss, operation time, resection margin, time to adjuvant chemotherapy initiation, readmission percentage, (liver-specific) morbidity, quality of life, body image, reasons for delay in discharge after functional recovery, long-term incidence of incisional hernias, hospital and societal costs during one year and overall five-year survival.

Secondary objectives – alternative hypotheses (H_1)
There is a difference between patients undergoing open or laparoscopic hemihepatectomy (with or without the need for one additional hepatic wedge resection or metastasectomy) for hospital length of stay, intraoperative blood loss, operation time, resection margin, time to adjuvant chemotherapy initiation, readmission percentage, (liver-specific) morbidity, quality of life, body image, reasons for delay in discharge after functional recovery, long-term incidence of incisional hernias, hospital and societal costs during one year and overall five-year survival.

ORANGE SEGMENTS - primary objective
To compare laparoscopic liver resection with open liver resection, for accepted indications and within an enhanced recovery programme in terms of time to functional recovery in patients undergoing parenchymal preserving resection of postero-superior liver segments.

Primary objective - null hypothesis (H_0)
There is no difference in time to functional recovery between patients undergoing open or laparoscopic parenchymal preserving resection of postero-superior liver segments.

Primary objective – alternative hypothesis (H_1)
There is a difference in time to functional recovery between patients undergoing open or laparoscopic parenchymal preserving resection of postero-superior liver segments.

ORANGE SEGMENTS – secondary objectives
To compare laparoscopic liver resection with open resection, for accepted indications and within an enhanced recovery programme in terms of hospital length of stay, intraoperative blood loss, operation time, resection margin, time to adjuvant chemotherapy initiation, readmission percentage, (liver-specific) morbidity, quality of life, body image, reasons for delay in discharge after functional recovery, long-term incidence of incisional hernias, hospital and societal costs during one year and overall five-year survival in patients undergoing parenchymal preserving resection of postero-superior liver segments.

Secondary objectives – null hypotheses (H_0)
There is no difference between patients undergoing parenchymal preserving resection of postero-superior liver segments in hospital length of stay, intraoperative blood loss, operation time, resection margin, time to adjuvant chemotherapy initiation, readmission percentage, (liver-specific) morbidity, quality of life, body image, reasons for delay in discharge after functional recovery, long-term incidence of incisional hernias, hospital and societal costs during one year and overall five-year survival.

Secondary objectives – alternative hypotheses (H_1)

There is a difference between patients undergoing open or laparoscopic parenchymal preserving resection of postero-superior liver segments for hospital length of stay, intraoperative blood loss, operation time, resection margin, time to adjuvant chemotherapy initiation, readmission percentage, (liver-specific) morbidity, quality of life, body image, reasons for delay in discharge after functional recovery, long-term incidence of incisional hernias, hospital and societal costs during one year and overall five-year survival.

1.3 Definition of outcomes

1.3.1 Primary outcome

Time to functional recovery is defined as the amount of time in days between the end of surgery and the moment of functional recovery. If available, time to functional recovery is measured in half days (recovery AM or PM) to increase precision. Functional recovery is defined as the moment a patient is deemed fit enough to be discharged from the hospital. Functional recovery is established if a patient meets a set of five criteria. These criteria are measured daily, from end of surgery until hospital discharge. A patient is considered functionally recovered if he or she:

**Functional recovery criteria**	
**1**	Has adequate pain control with oral analgesics only.
**2**	Is independently mobile; as objectified with a mobility score ≥8 or at the preoperative level.
**3**	Tolerates solid food.
**4**	Has a normal or decreasing serum TB (total bilirubin), ALT (alanine aminotransferase), AST (aspartate aminotransferase) and $0.9 \leq$ INR ≤ 1.4.
**5**	Is independent of intravenous fluid administration.

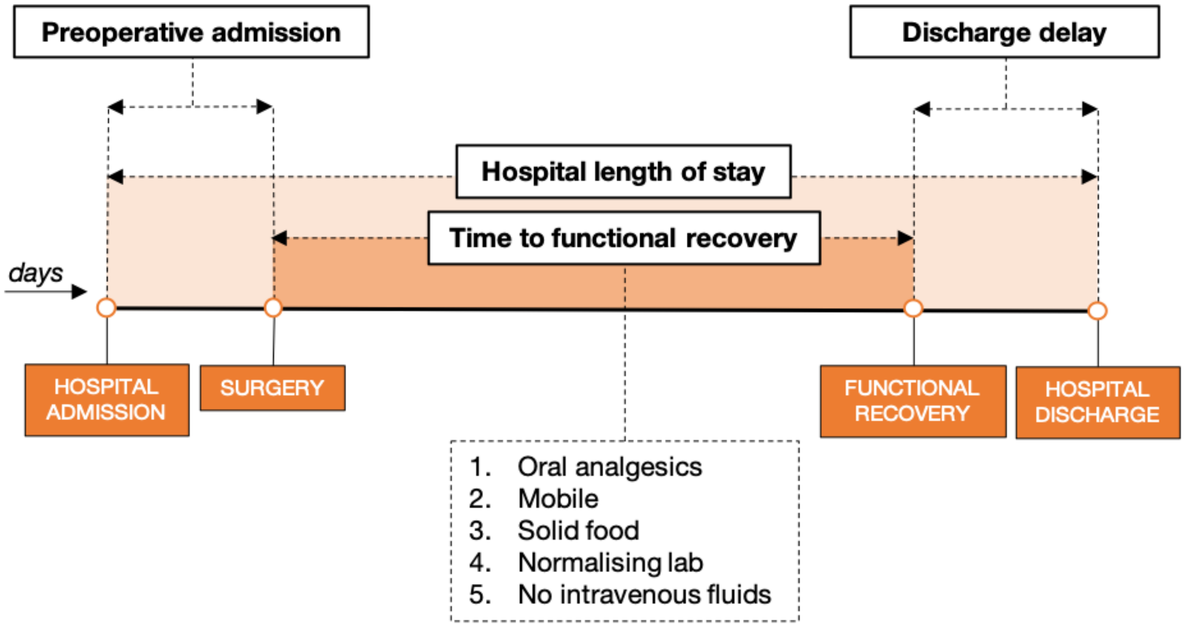

Time to functional recovery is expected to be a more reliable clinical outcome compared to hospital length of stay, as it is not affected by non-clinical matters such as administrative issues, problems in homecare support or logistic troubles. It is medically justified to discharge patients from the hospital if the functional recovery criteria have been met and if the patient is willing to go home.[1,2]

Calculating time to functional recovery
Time to functional recovery (days) $=$ Date (AM/PM)$_{\text{functional recovery}}$ $-$ Date (AM/PM)$_{\text{surgery closing}}$<br><br>Provided that <u>all</u> functional recovery criteria have been fulfilled:<br><br>Date (AM/PM)$_{\text{functional recovery}}$ $=$ Date (AM/PM)$_{\text{functional recovery criterium 1\&2\&3\&4\&5}}$<br><br>If one of the functional recovery criteria could <u>not</u> be fulfilled:<br><br>Date (AM/PM)$_{\text{functional recovery}}$ $=$ Date (AM/PM)$_{\text{hospital discharge}}$

1. Adequate pain control with oral analgesics

The postoperative moment any administration of pain medication other than oral administration is stopped.

Calculating time to adequate pain control with oral analgesics
Time to (oral)pain control (days) $=$ Date (AM/PM)$_{\text{oral pain control}}$ $-$ Date (AM/PM)$_{\text{surgery closing}}$

2. Independent mobility

The postoperative moment the patient has established a score of 8 or higher (or is at the preoperative level).

Mobility score

The mobility score has been adapted from the Groningen Activity Restriction Scale and globally assesses patient mobility before and after surgery by scoring 10 basic functional actions.[3]

**Mobility score**		
Can you fully independently:		YES = 1 point , NO = 0 points
1.	Feed yourself?	YES/NO
2.	Get in and out of bed?	YES/NO
3.	Stand up from sitting in a chair?	YES/NO
4.	Get on and off the toilet?	YES/NO
5.	Wash yourself?	YES/NO
6.	Dress yourself completely?	YES/NO
7.	Prepare breakfast or lunch (light meals)?	YES/NO
8.	Get around in the house/ward (if necessary, with a crane)?	YES/NO
9.	Walk over 100 meters (not only inside home)	YES/NO
10.	Go up and down the stairs?	YES/NO
TOTAL POINTS: ________		

Calculating mobility score

$$\text{Total mobility score} = \text{Sum Mobility Score Questions}_{1-10}$$

Calculating time to independent mobility

Provided that the preoperative mobility score was ≥8:

$$\text{Time to independent mobility (days)} = \text{Date (AM/PM)}_{\text{independent mobility} \geq 8} - \text{Date (AM/PM)}_{\text{surgery closing}}$$

Provided that the preoperative mobility score was <8:

$$\text{Time to independent mobility (days)} = \text{Date (AM/PM)}_{\text{preoperative mobility score}} - \text{Date (AM/PM)}_{\text{surgery closing}}$$

Solid food tolerance

The postoperative moment the patient tolerates (no reactive vomiting) any form of solid food.

Calculating time to solid food tolerance

$$\text{Time to solid food tolerance (days)} = \text{Date (AM/PM)}_{\text{solid food tolerance}} - \text{Date (AM/PM)}_{\text{surgery closing}}$$

Normalising laboratory values

The postoperative moment the patient shows normal or decreasing serum levels of total bilirubin (TB), alanine aminotransferase (ALT), aspartate aminotransferase (AST) and international normalised ratio (INR). If laboratory values have not been supplied in the requested unities, unities are converted using the appropriate conversion calculations (either manually with the molecular weights or using an online converter such as http://unitslab.com).

Normal serum concentrations	
**Total bilirubin**	<21 μmol/L
**Alanine aminotransferase**	<35 U/L (women) or <45 U/L (men)
**Aspartate aminotransferase**	<30 U/L (women) or <35 U/L (men)
**INR**	0.9 ≤ INR ≤ 1.4

Calculating time to normalised or decreasing serum concentrations
For each laboratory parameter (TB, ALT, AST or INR), the first moment in days for either time to normalisation or time to decrease of that specific laboratory parameter is used.<br><br>**For normalised values:**<br><br>$\text{Time to normal TB (days)} = \text{Date (AM/PM)}_{\text{TB<21μmol/L}}$ (women or wen)<br>$\text{Time to normal ALT (days)} = \text{Date (AM/PM)}_{\text{ALT<35 U/L}}$ (women) or $\text{Date (AM/PM)}_{\text{ALT<45 U/L}}$ (men)<br>$\text{Time to normal AST (days)} = \text{Date (AM/PM)}_{\text{AST<30 U/L}}$ (women) or $\text{Date (AM/PM)}_{\text{AST<35 U/L}}$ (men)<br>$\text{Time to normal INR (days)} = \text{Date (AM/PM)}_{0.9 \le \text{INR} \le 1.4}$ (women or wen)<br><br>**For decreasing laboratory values:***<br><br>$\text{Time to descrease TB (days)} = \text{Date (AM/PM)}_{\text{TB POD}_x - \text{TB POD}_{x-1} < 0}$<br>$\text{Time to descrease ALT (days)} = \text{Date (AM/PM)}_{\text{ALT POD}_x - \text{ALT POD}_{x-1} < 0}$<br>$\text{Time to descrease AST (days)} = \text{Date (AM/PM)}_{\text{AST POD}_x - \text{AST POD}_{x-1} < 0}$<br>$\text{Time to descrease INR (days)} = \text{Date (AM/PM)}_{\text{ALT POD}_x - \text{ALT POD}_{x-1} < 0}$
* POD_{x-1} meaning the previous postoperative day that specific laboratory parameter was determined. This may be 1 day before, 2 days before or more.

Intravenous fluid infusion stop

The postoperative moment extra intravenous fluid administration is stopped. Infusion to maintain intravenous line patency is allowed.

Calculating time to fluid infusion stop
$\text{Time to fluid infusion stop (days)} = \text{Date (AM/PM)}_{\text{fluid infusion stop}} - \text{Date (AM/PM)}_{\text{surgery closing}}$

1.3.2 Secondary outcomes

Secondary endpoints include hospital length of stay, intraoperative blood loss, operation time, intraoperative incidents, conversions, (liver specific) morbidity, 90-day mortality, readmission percentage, resection margin, quality of life, body image, reasons for delay in discharge after functional recovery, long-term incidence of incisional hernias, hospital and societal costs during one year, time to adjuvant chemotherapy initiation and overall five-year survival.

Hospital length of stay

Hospital length of stay is defined as the amount of time in days between hospital admission and hospital discharge.

Calculating hospital length of stay
$\text{Hospital lenght of stay (days)} = \text{Date (AM/PM)}_{\text{hospital discharge}} - \text{Date (AM/PM)}_{\text{hospital admission}}$

In general, patients are admitted the day before surgery. This may however differ per hospital, as sometimes patients are referred from far away.

Calculating preoperative admission time
$\text{Preoperative admission time (days)} = \text{Date (AM/PM)}_{\text{surgery incision}} - \text{Date (AM/PM)}_{\text{hospital admission}}$

Patients may also be discharged quite some time after functional recovery. The reasons for delay in discharge after functional recovery are also registered.

Calculating discharge delay time
$\text{Discharge delay time (days)} = \text{Date (AM/PM)}_{\text{hospital discharge}} - \text{Date (AM/PM)}_{\text{functional recovery}}$

Intraoperative blood loss

Intraoperative blood loss is defined as the amount of blood loss in millilitres (mL) during surgery. Intraoperative blood loss is measured by the anaesthesiologist by summing the amount of blood that has been sucked into the suction jar(s) (and not used for re-entry) and the amount of blood that has been absorbed by surgical compresses (based on weight differences of the compresses before and after use). If the compresses could not be weighed, an estimate suffices.

Calculating intraoperative blood loss
$\text{Intraoperative blood loss (mL)} = \text{Blood loss}_{\text{suction jar}} + \text{Blood loss}_{\text{surgical compresses}}$

Operation time
Operation time is the amount of time in minutes between time of incision and time of wound(s) closure (stitching). Extension of operation time due to hardware malfunctioning has to be registered (Satava 1) and may be subtracted from the total duration. Any other delay in operation time, such as for ultrasound examination or liver mobilisation, is not subtracted, as these are essential to both open and laparoscopic liver surgery.

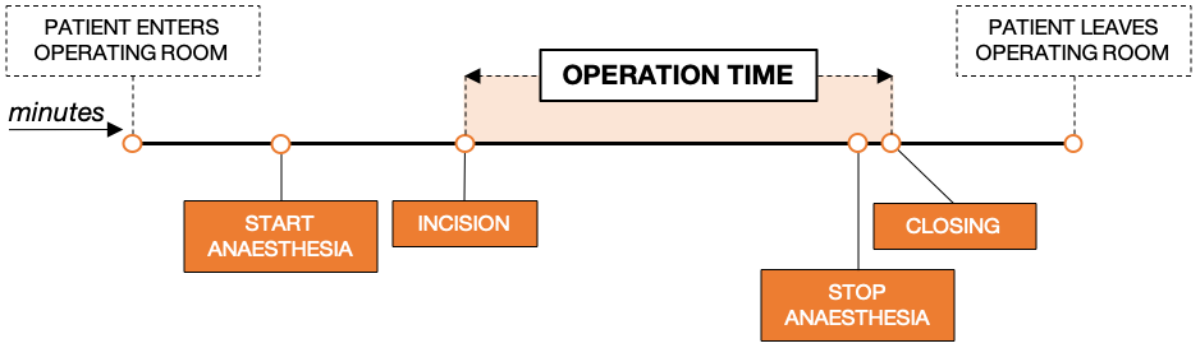

**Calculating operation time**
Operation time (minutes) $=$ $\text{Time}_{\text{patient closing}} - \text{Time}_{\text{incision}}$

Conversion
Conversions are defined as those operations that start laparoscopically and are changed during surgery to either hand-assisted laparoscopic surgery or open surgery (laparotomy). Conversions are provided in numbers and as percentages of the total number of patients. Reasons for conversion are registered categorically and descriptively.

**Conversion categories**	
**Not applicable** (open resection or no conversion)	
**Urgent reason (s)**	Bleeding<br>Unintentional damage to surrounding structures<br>Patient hemodynamic instability<br>Other urgent reason(s)
**Non-urgent reason(s)**	Adhesions<br>Inability to identify anatomical structures<br>Limited visibility<br>Tumour too large<br>Lack of progression<br>Poor quality liver parenchyma<br>Continuous bleeding<br>Uncertainty of oncologic margins<br>Equipment problems<br>Other non-urgent reason(s)
**Unknown reason**	

Unfavourable intraoperative incidents

Unfavourable intraoperative incidents are defined as any unintended incidents during surgery that may have caused harm to the patient. Unfavourable intraoperative incidents are graded with the Satava approach to surgical error evaluation, adapted for liver surgery.[4,5] Unfavourable incident grades are provided categorically in numbers and as percentage of the total number of patients.

The Satava approach to surgical error evaluation (adapted for liver surgery) categories	
**Grade**	**Definition of intraoperative unfavourable incidents**
**Grade I**	Incidents managed without change of operative approach and without further consequences for the patient. It includes perforations of adherent or adjacent organs and minor change of intraoperative tactics and cases with blood loss over normal range (corresponding to blood loss over 1000 mL in case of liver resection).
**Grade II**	Incidents with consequences for patient. It includes cases requiring limited resection of intra-operatively injured organs or cases with blood loss which is appreciably over normal range (corresponding to blood loss over 2000 mL in case of liver resection). In case of laparoscopic approach, it also includes cases requiring conversion to open approach. Conversion to hand-assisted laparoscopy is not regarded a grade II incident.
**Grade III**	Incident leading to significant consequences for patient.

Postoperative morbidity

<u>Adverse events</u>

Morbidity is defined as a state of illness or lack of health that includes physical, mental, or emotional disability. Postoperative morbidity can occur without postoperative complication. Postoperative complication/adverse event is defined as any deviation from the normal postoperative course. (Postoperative) complications of the underlying disease that are not related to the surgery are not adverse events (e.g. metastasis).

Until 90 days after surgery, all adverse events are classified in accordance with the Accordion Severity Grading System of Surgical Complications and the Clavien-Dindo classification.[6-8] Adverse event duration and outcome are also registered. In addition to the Accordion and Clavien-Dindo classifications, adverse event impact is also calculated per patient using the Comprehensive Complication Index, which also accounts for adverse event multiplicity.[9] The Comprehensive Complication Index (CCI) ranges from 0 (no burden from complication) to 100 (death) and can be calculated online (https://www.assessurgery.com) using the Clavien-Dindo score or scores. Based on the CCI and the age of the patient, also a cost prediction after liver surgery can be generated.

Adverse events may occur simultaneously or in sequence. Often, one adverse event may lead to another more severe (e.g. a surgical site infection leading to sepsis). For multiple adverse events occurring in the same patient, all events are registered. Adverse events are provided in numbers per grade and as percentages of the total number of patients.

The Accordion severity grading system of surgical complications categories	
**Grade**	**Definition of postoperative complications**
**Grade I**	Mild complication.<br>Requires only minor invasive procedures that can be done at the bedside such as insertion of intravenous lines, urinary catheters, and nasogastric tubes, and drainage of wound infections. Physiotherapy and the following drugs are allowed: antiemetics, antipyretics, analgesics, diuretics and electrolytes.
**Grade II**	Moderate complication.<br>Requires pharmacologic treatment with drugs other than such allowed for minor complications, for instance antibiotics. Blood transfusions and total parenteral nutrition are also included.
**Grade III**	Severe complication.<br>Invasive procedure without general anaesthesia.<br>Requires management by an endoscopic, interventional procedure or re-operation without general anaesthesia.
**Grade IV**	Severe complication.<br>Operation under general anaesthesia.<br>Requires management by an operation under general anaesthesia.
**Grade V**	Severe complication.<br>Organ system failure.
**Grade VI**	Death.<br>Postoperative death.

The Clavien-Dindo classification system of surgical complications categories	
**Grade**	**Definition of postoperative complications**
Grade I	Any deviation from the normal postoperative course without the need for pharmacological treatment or surgical, endoscopic, and radiological interventions.<br><br>Allowed therapeutic regimens are: drugs as antiemetics, antipyretics, analgesics, diuretics, electrolytes, and physiotherapy. This grade also includes wound infections opened at the bedside.
Grade II	Requiring pharmacological treatment with drugs other than such allowed for grade I complications Blood transfusions and total parenteral nutrition are also included.
Grade III<br><br>Grade IIIA<br>Grade IIIB	Requiring surgical, endoscopic or radiological intervention.<br><br>Intervention not under general anaesthesia.<br>Intervention under general anaesthesia.
Grade IV<br><br>Grade IVA<br>Grade IVB	Life-threatening complication (including CNS complications) requiring IC/ICU management.<br><br>Single organ dysfunction (including dialysis).<br>Multiorgan dysfunction.
Grade V	Death of a patient.
Suffix 'D'	If the patient suffers from a complication at the time of discharge, the suffix "d" (for "disability") is added to the respective grade of complication. This label indicates the need for a follow-up to fully evaluate the complication.

The comprehensive complication index categories			
**Grade**	**Definition of postoperative complications**	**Weight**	**Single value**
**Grade I**	Any deviation from the normal postoperative course without the need for pharmacological treatment or surgical, endoscopic, and radiological interventions.<br><br>Allowed therapeutic regimens are antiemetics, antipyretics, analgesics, diuretics, electrolytes, and physiotherapy. This grade also includes wound infections opened at the bedside.	300	8.7
**Grade II**	Requiring pharmacological treatment with drugs other than such allowed for grade I complications Blood transfusions and total parenteral nutrition are also included.	1750	20.9
**Grade III**<br><br>**Grade IIIA**<br>**Grade IIIB**	Requiring surgical, endoscopic or radiological intervention.<br><br>Intervention not under general anaesthesia.<br>Intervention under general anaesthesia.	-<br><br>2750<br>4550	-<br><br>26.2<br>33.7
**Grade IV**<br><br>**Grade IVA**<br>**Grade IVB**	Life-threatening complication (including CNS complications) requiring IC/ICU management.<br><br>Single organ dysfunction (including dialysis).<br>Multiorgan dysfunction.	-<br><br>7200<br>8550	-<br><br>42.4<br>46.2
**Grade V**	Death of a patient.	-	-

Calculating the comprehensive complication index

The exact source formula is nowhere provided, however the basic formula used online is like this. (Be aware that according to this formula, patients with a weight sum >40.000 could theoretically have a score >100):

$$\text{Comprehensive compication index} = \frac{\sqrt{(\text{Weight}_1 + \text{Weight}_2 + \text{Weight}_n)}}{2}$$

Scores >100 (theoretically possible, practically highly unlikely) are adjusted to 100.<br>Clavien-Dindo grade V always results in a comprehensive complication index of 100.

To facilitate scoring postoperative complications, a list with the most common postoperative complications after liver surgery is provided as a guideline. Obviously, this list may not cover all postoperative complications after liver surgery, so researchers may need to classify a postoperative complication as 'other' and elaborate.

**Most common postoperative complications after liver surgery categories**
<u>Pleural effusion</u><br>Any fluid in the pleural cavity proven by radiological imaging.
<u>Ascites</u><br>Intra-abdominal clear fluid evidenced by leakage of more than 500 mL/day via the abdominal wound or, if present, the abdominal drain from postoperative day 3 onwards.
<u>Wound infection</u><br><br>*Superficial incisional surgical-site infection.*<br>Infection occurs within 30 days after operation, and involves only skin or subcutaneous tissue of the incision and at least one of the following:<br>1. Purulent drainage, with or without laboratory confirmation, from the superficial incision.<br>2. Organisms isolated from an aseptically obtained culture of fluid or tissue from the superficial incision.<br>3. At least one of the following signs or symptoms of infection: pain or tenderness, localized swelling, redness or heat, and superficial incision is deliberately opened by surgeon, unless incision is culture-negative.<br>4. Diagnosis of superficial incisional surgical-site infection (SSI) by the surgeon or attending physician.<br><br>*Deep incisional surgical-site infection.*<br>Infection occurs within 30 days after the operation, appears to be related to the operation, and involves deep soft tissues of the incision and at least one of the following:<br>1. Purulent drainage from the deep incision but not from the organ/space component of the surgical site.<br>2. A deep incision spontaneously dehisces or is deliberately opened by a surgeon when the patient has at least one of the following signs or symptoms: fever (over 38ºC), localized pain or tenderness, unless site is culture-negative.<br>3. An abscess or other evidence of infection involving the deep incision is found on direct examination, during reoperation, or by histopathological or radiological examination.<br>4. Diagnosis of a deep incisional SSI by a surgeon or attending physician
<u>Intra-abdominal abscess</u><br>Any quantity of purulent fluid leaking via the abdominal drain; walled-off collection of pus in the abdominal cavity at the time of radiological imaging, reoperation or percutaneous drainage; fluid in drain or intra-abdominal collection should be culture-positive.
<u>Bile leakage</u><br>Any quantity of bile leaking via the abdominal wound or drains at least 48 hours after surgery; intra-abdominal collection of bile at the time of radiological imaging, reoperation or percutaneous drainage; cholangiography evidence of contrast leakage. Fluid in drain or intra-abdominal collection should have a bilirubin content at least three times higher than the upper normal serum level in patients with normal serum bilirubin levels after surgery, or a 50 per cent higher serum bilirubin level than that in patients with raised serum bilirubin levels after surgery.
<u>Sepsis</u>

Defined according to the Centers for Disease Control and Prevention. Sepsis is the clinical syndrome defined by the presence of both infection and a systemic inflammatory response.

Infection
Pathological process caused by invasion of normally sterile tissue or fluid or body cavity by pathogenic or potentially pathogenic microorganisms.

Systemic inflammatory response syndrome
Body temperature higher than 38°C or lower than 36°C; heart rate higher than 90 beats/minute; hyperventilation evidenced by respiratory rate higher than 20 per minute or arterial partial pressure of carbon dioxide lower than 32 mmHg; white blood cell count higher than 12.000 cells/μL or lower than 4.000 cells/μL.

Postoperative liver failure
Postoperative failure of one or more of the hepatic excretory, detoxifying and synthetic functions, including serum bilirubin level over 50 μmol/L (2.9 mg/dL), prothrombin index less than 50 per cent (international normalized ratio greater than 1.7) and/or hepatic encephalopathy (HE) grade 3 or 4 from day 3 after surgery onwards. Other obvious causes of the observed deterioration should be excluded (such as biliary obstruction). Moderate postoperative liver failure (PLF): decreased prothrombin time (PT), increased bilirubin and no HE present. Severe PLF: decreased PT, increased bilirubin and HE grade 3 or 4 present.

Pneumonia
Defined according to the Centers for Disease Control and Prevention. Pneumonia must meet one of the following criteria.

1. Rales or dullness to percussion on physical examination on chest AND any of the following:

   - ☐ New onset of purulent sputum or change in character of sputum.
   - ☐ Organism isolated from blood culture.
   - ☐ Isolation of pathogen from specimen obtained by transtracheal aspirate, bronchial brushing, or biopsy.

2. Chest radiographic examination shows new or progressive infiltrate, consolidation, cavitation, or pleural effusion AND any of the following:

   - ☐ New onset of purulent sputum or change in character of sputum.
   - ☐ Organism isolated from blood culture.
   - ☐ Isolation of pathogen from specimen obtained by transtracheal aspirate, bronchial brushing, or biopsy.
   - ☐ Isolation of virus or detection of viral antigen in respiratory secretions.
   - ☐ Diagnostic single antibody titre (IgM) or fourfold increase in paired serum samples (IgG) for pathogen.
   - ☐ Histopathological evidence of pneumonia.

Intra-abdominal haemorrhage
Any quantity of blood leaking via the abdominal drain either with or without a drop in haemoglobin concentrations; intra-abdominal active bleeding during radiological imaging or reoperation. Other sources of haemorrhage should be excluded.

Intra-abdominal haematoma
Collection of blood in the proximity of the liver that can be diagnosed either radiologically or during reoperation.

Acute renal failure<br>Increase in serum creatinine of 0.5 mg/dL (44.2 µmol/L) if baseline is less than 2.5 mg/dL (221 µmol/L, or an increase in serum creatinine by more than 20 per cent if the baseline level is more than 2.5 mg/dL (221 µmol/L.
<u>Blood transfusion</u><br>Administration of any quantity of packed cells during or within 48 hours after surgery.
<u>Operative mortality</u><br>Death of a patient during or after the initial surgical procedure that is related to the surgical procedure.
<u>Other postoperative complication</u>

<u>Serious adverse events</u>
Adverse events are regarded serious if these:

**Serious adverse event classification categories**	
**1**	Lead to an admission longer than (>) 10 days.
**2**	Lead to a readmission within 30 days after surgery.
**3**	Are life-threatening (e.g. requiring intensive care admittance).
**4**	Lead to permanent or serious invalidity.
**5**	Lead to death within 90 days after surgery.

Multiple serious adverse events may occur in one patient (e.g. readmission leading to intensive care admission. All serious adverse events are registered. Serious adverse events are provided in numbers per category and as percentages of the total number of patients.

Composite endpoint of morbidity after liver surgery
The composite endpoint of morbidity after liver surgery was suggested by van den Broek et al. and is composed of a combination of procedure-specific complications.[10] The composite endpoint is considered a single, dichotomous outcome. One may thus not differentiate for multiplicity of morbidity after liver surgery using this endpoint. All included complications are specific to liver surgery and have substantial clinical relevance, reflecting Clavien-Dindo grade 3-5 complications.[8888888888888888888888888888888888888888888888888888] Composite liver morbidity is provided in numbers and as percentages of the total number of patients.

**Composite endpoint of morbidity after liver surgery complications**
One or more of the following complications:<br><br>☐ Operative mortality<br>☐ Intra-abdominal haemorrhage

☐	Ascites
☐	Bile leakage
☐	Intra-abdominal abscess
☐	Postoperative liver failure

90-day mortality
90-Day mortality is defined as the death of a patient within 90 days after surgery. 90-Day mortality numbers are extracted from the adverse and serious adverse events data (Accordion grade VI, Clavien-Dindo grade V). 90-Day mortality numbers are categorised into surgery-related and disease-related deaths. 90-Day mortality is provided both descriptively and in numbers and as percentages of the total number of patients.

Readmission
Readmission is defined as the admission of a patient to any hospital after hospital discharge within 30 days of liver surgery. Readmission data can be extracted from the serious adverse event registrations. Readmission is categorised into surgery-related and non surgery-related readmission. Readmission duration is also registered. Readmission is provided in numbers and as percentages of the total number of patients.

Resection margin
The resection margin, also the surgical margin, is defined as the smallest distance between the resection plane and tumorous tissue of the resected liver specimen. The resection margin is measured in millimetres and is categorised based on its size.

**Resection margin categories**	
**R0**	No residual tumour. A resection margin size >1 mm.
**R1**	Microscopic residual tumour. A resection margin size ≤1 mm.
**R2**	Macroscopic residual tumour. Tumour tissue invasion of the resection plane visible with the naked eye.
**RX**	Presence of residual cannot be assessed.

Resection margin is provided per grade in numbers and as percentages of the total number of patients.

Quality of life
Health-related Quality of Life (HRQoL) is defined as how well a person functions in their life and his or her perceived well-being in the physical, mental, and social domains of health. HRQoL is measured using three different types of questionnaires: the EuroQol EQ-5D-3L, the EORTC QLQ-C30 and the EORTC QLQ-LMC21.[11-15]

<u>EuroQol EQ-5D-3L</u>
The EuroQol EQ-5D-3L is a standardised instrument to evaluate a person's health state. It contains five questions/'dimensions' (mobility, self-care, usual activities, pain/discomfort and anxiety/depression. Each question may be answered on three levels (1 – no problems, 2 – some/moderate problems, 3 – extreme problems). In addition to these 5 questions, the EQ-5D-3L also contains a visual analogue scale (VAS) score (0-100).

From the 5 questions a 5-digit summary score for health state is derived. So, according to this system, full health is indicated by 11111 and the poorest health state by 33333. This way, a total of 243 possible health states can be defined (3 × 3 × 3 × 3 × 3). For the EQ-5D-3L to be used in evaluating the health-related quality of life (HRQoL) benefits associated with the type of surgery, either as single index measures for clinical purposes or as estimations

of Quality Adjusted Life Years (QALYs), it is necessary to derive a single 'index value' for each of these health states described by the 5 -digit number. The index value lies between 1 (= full health) and 0 (=dead). However, the index value of the particular health state may vary between countries, regions and even populations (based on age, gender, social-economic status, etc.). That is the reason why the index value of each health state is attached to a particular set of 'weights'. These weights are commonly referred to as 'value sets' (previously also referred to as 'tariffs'), commonly derived using Time Trade-Off (TTO) techniques.

First, a European-specific value set is used to derive the single index value per patient, which thus reflects how good or bad a health state is according to the general European population. In addition to the European value set, additional calculations are conducted per country with country specific value sets (Belgium for the Belgian centres, Germany for the German centre, Italy for the Italian centres, Netherlands for the Dutch centres, Denmark and Sweden for the Norwegian centre and United Kingdom for the centres from the United Kingdom).[12,16-21]

EQ-5D-3L health index values and VAS-scores are provided in numbers. Besides between- and within-patient (baseline versus follow-up), the health index values and VAS-scores also allow for comparisons with populations norms.[22] In addition to the health index values, the EQ-5D-3L data are also reported on the dimensional level as numbers and as percentages of the total number of patients.

EQ-5D-3L value sets (Europe wide and country-specific)

General formula

Index value = $1 - (c_{MO2} \times MO2) - (c_{MO3} \times MO3) - (c_{SC2} \times SC2) - (c_{SC3} \times SC3) - (c_{UA2} \times UA2) - (c_{UA3} \times UA3) - (c_{PD2} \times PD2) - (c_{PD3} \times PD3) - (c_{AD2} \times AD2) - (c_{AD3} \times AD3) - (c_{N2} \times N2) - (c_{A3} \times N3) - (c_{OTHER} \times OTHER)$

Variable		Level 1 – No problems	Level 2 – Some or moderate problems	Level 3 – Extreme problems
Mobility = 2	MO2	0	1	0
Mobility = 3	MO3	0	0	1
Self-care = 2	SC2	0	1	0
Self-care = 3	SC3	0	0	1
Usual activities = 2	UA2	0	1	0
Usual activities = 3	UA3	0	0	1
Pain/discomfort = 2	PD2	0	1	0
Pain/discomfort = 3	PD3	0	0	1
Anxiety/depression = 2	AD2	0	1	0
Anxiety/depression = 3	AD3	0	0	1
≥1 dimension = 2 or 3	N2	0	1	0
≥1 dimension = 3	N3	0	0	1
>1 dimension = 2 or 3	D1	0	0 or 1	0 or 1

Country	N	Method	Year	C_{MO2}	C_{MO3}	C_{SC2}	C_{SC3}	C_{UA2}	C_{UA3}	C_{PD2}	C_{PD3}	C_{AD2}	C_{AD3}	C_{N2}	C_{N3}	C_{OTHER}
**Europe**	8709	VAS	2003	0.066	0.183	0.117	0.156	0.026	0.086	0.093	0.164	0.089	0.129	0.128	0.229	-
**Belgium**	548	VAS	2009	0.074	0.148	0.083	0.166	0.031	0.062	0.084	0.168	0.103	0.206	0.152	0.256	-
**Denmark**	1332	TTO	2009	0.053	0.411	0.063	0.192	0.048	0.144	0.062	0.396	0.068	0.367	0.114	-	-
**Germany**	339	TTO	2005	0.099	0.327	0.087	0.174	-	-	0.112	0.315	-	0.065	0.001	0.323	-
**Italy**	439	TTO	2013	0.076	0.518	0.100	0.289	0.085	0.198	0.098	0.334	0.095	0.213	-	-	D1: -0.043
**Netherlands**	309	TTO	2006	0.036	0.161	0.082	0.152	0.032	0.057	0.086	0.329	0.124	0.325	0.071	0.234	-
**Sweden**	45477	TTO	2014	0.066	0.150	0.048	-0.044	0.099	0.113	0.035	0.076	0.055	0.195	0.031	0.061	-
**United Kingdom**	3395	TTO	1997	0.069	0.314	0.104	0.214	0.036	0.094	0.123	0.386	0.071	0.236	0.081	0.269	-

In light of quality-adjusted life years (QALYs) calculations, health index values are synonymous for utilities. QALYs can thus be directly calculated from the utilities with the following formula:

Deriving QALY from EQ-5D-3L
QALY = Health index × Δtime (years)<br><br>$\Delta\text{time (years)} = \frac{\text{date } X_{1/2/3/4/5} - \text{date } X_{-1/1/2/3/4}}{365{,}25}$<br><br>The difference in time between 2 measurements must be calculated in years. This can be achieved by calculating the amount of days between the measurements (follow-up moments) and dividing it by the amount of days in a year, which is 365,25. The total quality of life over a specific period can be determined by summing the quality of life of all individual periods, which is basically determining the area under the curve. The total area under the curve can be calculated by adding al the QALYs per time period.

EORTC QLQ-C30

The EORTC QLQ-C30 is an integrated system for assessing the health- related quality of life of cancer patients participating in international clinical trials. The QLQ-C30 is composed of both multi-item scales and single-item measures. These include five functional scales, three symptom scales, a global health status scale, and six single items. Each of the multi-item scales includes a different set of items - no item occurs in more than one scale. All of the scales and single-item measures range in score from 0 to 100. A high scale score represents a higher response level. Thus, a high score for a functional scale represents a high / healthy level of functioning, a high score for the global health status represents a high QoL, but a high score for a symptom scale / item represents a high level of symptomatology / problems. The principle for scoring these scales (either directly or with provided matrices) is the same in all cases:

1. Estimate the average of the items that contribute to the scale; this is the *raw score*.
2. Use a linear transformation to standardise the raw score, so that scores range from 0 to 100; a higher score represents a higher ('better') level of functioning, or a higher ('worse') level of symptoms.

Calculating the EORTC QLQ-C30 score
In practical terms, if questions $Q_1, Q_2, \dots Q_n$ are included in a scale, the procedure is as follows:<br><br>$\text{Raw score} = \frac{Q_1 + Q_1 + \ldots + Q_n}{n}$<br><br>To obtain the score *S*, a linear transformation to 0-100 is applied:<br><br>Functional scale: $S_{\text{functional}} = \left\{1 - \frac{(\text{Raw score} - 1)}{\text{range}}\right\} \times 100$<br><br>Symptom scale: $S_{\text{symptoms}} = \left\{\frac{(\text{Raw score} - 1)}{\text{range}}\right\} \times 100$<br><br>Global health scale: $S_{\text{global health}} = \left\{\frac{(\text{Raw score} - 1)}{\text{range}}\right\} \times 100$<br><br>$\text{Range} = \text{Raw score}_{\text{maximum}} - \text{Raw score}_{\text{minimum}}$<br><br>The range in most items is the same, as these are scored 1 to 4, giving a range = 3. The exceptions are the items contributing to the global health status, which are 7-point questions with a range = 6.

The EORTC provides a validated manual (Quality of Life Unit, EORTC Data Center, Avenue E. Mounier 83 - B11, 1200 Brussels, Belgium) with standardised SPSS syntax, which includes missing data imputation, to calculate the scales. The data in this trial will be calculated and analysed in accordance with this manual.[23]

Scoring the EORTC QLQ-C30

	Scale	Number of items	Item range	Item numbers
**Global health status / quality of life scale**				
**Global health status**	QL2	2	6	29, 30
**Functional scales**				
**Physical functioning**	PF2	5	3	1, 2, 3, 4, 5
**Role functioning**	RF2	2	3	6, 7
**Emotional functioning**	EF	4	3	21, 22, 23, 24
**Cognitive functioning**	CF	2	3	20, 25
**Social functioning**	SF	2	3	26, 27
**Symptom scales**				
**Fatigue**	FA	3	3	10, 12, 18
**Nausea and vomiting**	NV	2	3	14, 15
**Pain**	PA	2	3	9, 19
**Dyspnoea**	DY	1	3	8
**Insomnia**	SL	1	3	11
**Appetite loss**	AP	1	3	13
**Constipation**	CO	1	3	16
**Diarrhoea**	DI	1	3	17
**Financial difficulties**	FI	1	3	28

Calculating the EORTC QLQ-C30 scales

The following calculations are used in the EORTC QLQ-C30 manual.

Global health status / quality of life scale

$$\text{Global health status} = \left\{ \frac{\left(\left(\frac{Q_{29} + Q_{30}}{2}\right) - 1\right)}{(7-1)} \right\} \times 100$$

Functional scales

$$\text{Physical functioning} = \left\{ 1 - \frac{\left(\left(\frac{Q_1 + Q_2 + Q_3 + Q_4 + Q_5}{5}\right) - 1\right)}{(4-1)} \right\} \times 100$$

$$\text{Role functioning} = \left\{ 1 - \frac{\left(\left(\frac{Q_6 + Q_7}{2}\right) - 1\right)}{(4-1)} \right\} \times 100$$

$$\text{Emotional functioning} = \left\{ 1 - \frac{\left(\left(\frac{Q_{21} + Q_{22} + Q_{23} + Q_{24}}{4}\right) - 1\right)}{(4-1)} \right\} \times 100$$

$$\text{Cognitive functioning} = \left\{1 - \frac{\left(\left(\frac{Q_{20} + Q_{25}}{2}\right) - 1\right)}{(4-1)}\right\} \times 100$$

$$\text{Social functioning} = \left\{1 - \frac{\left(\left(\frac{Q_{26} + Q_{27}}{2}\right) - 1\right)}{(4-1)}\right\} \times 100$$

Symptom scales

$$\text{Fatigue} = \left\{\frac{\left(\left(\frac{Q_{10} + Q_{12} + Q_{18}}{3}\right) - 1\right)}{(4-1)}\right\} \times 100$$

$$\text{Nausea and vomiting} = \left\{\frac{\left(\left(\frac{Q_{14} + Q_{15}}{2}\right) - 1\right)}{(4-1)}\right\} \times 100$$

$$\text{Pain} = \left\{\frac{\left(\left(\frac{Q_{9} + Q_{19}}{2}\right) - 1\right)}{(4-1)}\right\} \times 100$$

$$\text{Dyspnoea} = \left\{\frac{\left(\left(\frac{Q_{8}}{1}\right) - 1\right)}{(4-1)}\right\} \times 100$$

$$\text{Insomnia} = \left\{\frac{\left(\left(\frac{Q_{11}}{1}\right) - 1\right)}{(4-1)}\right\} \times 100$$

$$\text{Appetite loss} = \left\{\frac{\left(\left(\frac{Q_{13}}{1}\right) - 1\right)}{(4-1)}\right\} \times 100$$

$$\text{Constipation} = \left\{\frac{\left(\left(\frac{Q_{16}}{1}\right) - 1\right)}{(4-1)}\right\} \times 100$$

$$\text{Diarrhoea} = \left\{\frac{\left(\left(\frac{Q_{17}}{1}\right) - 1\right)}{(4-1)}\right\} \times 100$$

$$\text{Financial difficulties} = \left\{ \frac{\left(\left(\frac{Q_{28}}{1} \right) - 1 \right)}{(4 - 1)} \right\} \times 100$$

Presenting the EORTC QLQ-C30 scores

The calculated EORTC QLQ-C30 scores are provided in numbers ranging from 0-100. Between-treatment arms and individual-patient (baseline versus follow-up) analyses are performed. In addition, the QLQ-C30 scores may also be compared with available QLQ-C30 reference values:

1. EORTC reference set.[24]
2. Normative population.[25]
3. Patients with cancer.[26]

EORTC normative QLQ-C30 reference values																
Variable		Normative population														
EORTC		All	Male							Female						
			All	18-29	30-39	40-49	50-59	60-69	≥70	All	18-29	30-39	40-49	50-59	60-69	≥70
Global health status	QL	75.0	75.5	82.9	83.3	80.0	74.7	71.6	62.3	74.5	84.3	82.6	77.7	74.4	69.0	60.2
Physical functioning	PF	92.2	92.9	97.8	98.5	96.3	93.6	92.3	79.7	91.5	97.0	97.3	95.0	92.9	89.2	78.0
Role functioning	RF	90.4	90.9	95.6	97.1	93.2	91.1	90.7	78.3	89.9	97.3	94.0	92.6	91.9	87.5	76.2
Emotional functioning	EF	83.5	83.9	84.4	89.0	83.7	80.9	85.6	81.0	83.2	86.0	85.6	81.9	82.2	83.5	80.2
Cognitive functioning	CF	93.5	93.7	96.3	98.2	94.6	94.3	94.2	85.1	93.4	96.9	96.0	93.5	94.5	93.0	86.3
Social functioning	SF	93.4	93.6	96.3	97.5	94.0	93.5	94.1	86.8	93.3	97.1	94.5	94.1	93.6	93.2	87.2
Fatigue	FA	15.5	14.5	10.8	6.9	10.6	14.5	14.5	28.7	16.4	10.1	12.1	14.4	15.6	18.3	27.9
Nausea and vomiting	NV	2.2	1.9	2.1	0.4	0.7	2.0	2.4	3.3	2.4	2.0	2.7	1.8	1.6	2.6	3.9
Pain	PA	16.7	16.4	7.8	6.3	13.1	16.8	20.6	32.3	17.0	7.2	9.0	13.2	16.0	24.2	31.6
Dyspnoea	DY	7.5	7.8	2.3	1.3	3.2	8.1	8.5	22.3	7.2	2.1	3.4	5.0	6.4	9.3	16.7
Insomnia	SL	12.4	11.6	6.6	6.5	9.8	13.2	11.9	20.4	13.0	5.6	7.7	10.6	15.8	15.7	21.3
Appetite loss	AP	3.8	3.2	2.1	1.0	2.4	4.3	2.0	7.1	4.2	3.4	3.4	4.4	3.2	4.0	7.0
Constipation	CO	2.2	1.3	0.7	0.6	0.9	1.6	0.7	2.9	3.1	1.4	1.6	2.9	2.9	4.0	5.6
Diarrhoea	DI	2.5	2.1	1.0	0.6	1.7	2.6	2.8	3.2	2.9	3.5	3.4	4.0	1.5	2.3	3.0
Financial difficulties	FI	4.8	4.8	2.6	1.9	5.1	5.5	3.5	10.1	4.8	2.6	2.0	5.6	4.5	4.8	9.3

EORTC cancer, colorectal cancer and liver cancer QLQ-C30 reference values																				
Variable		Cancer									Colorectal cancer									Liver cancer
EORTC		All	Male	Female	<50	50-59	60-69	≥70	Stage III-IV	Meta	All	Male	Female	<50	50-59	60-69	≥70	Stage III-IV	Meta	All
Global health status	QL	61.3	62.9	59.3	61.4	61.2	61.8	60.6	61.5	56.3	60.7	62.1	58.8	59.8	61.6	61.7	58.7	60.7	60.7	55.9
Physical functioning	PF	76.7	78.5	74.4	80.2	78.0	76.3	72.1	71.2	75.8	79.2	83.0	75.2	80.2	78.1	79.3	-	-	73.3	74.1
Role functioning	RF	70.5	73.4	67.1	68.6	69.4	72.6	70.7	70.6	60.7	70.4	72.3	68.4	68.1	68.7	72.3	70.8	80.0	64.8	65.2
Emotional functioning	EF	71.4	73.9	67.8	69.2	69.0	71.8	76.1	71.5	68.7	68.9	70.8	65.7	65.6	68.4	69.5	70.7	67.1	70.7	69.8
Cognitive functioning	CF	82.6	83.7	80.9	82.9	83.2	83.1	81.0	83.2	80.5	85.2	86.7	83.7	85.0	85.8	85.8	83.5	86.5	83.4	79.0
Social functioning	SF	75.0	76.3	72.9	72.1	83.3	76.4	78.2	75.1	70.5	76.0	76.7	75.8	68.9	73.7	77.6	80.9	76.7	74.3	69.0
Fatigue	FA	34.6	32.4	37.7	33.9	34.4	34.1	35.7	34.7	41.8	34.7	30.9	39.1	37.3	33.5	33.4	36.4	30.7	38.9	41.2
Nausea and vomiting	NV	9.1	7.7	11.1	9.4	8.9	8.7	9.1	7.8	13.1	7.3	5.4	9.6	9.5	7.3	6.9	6.3	4.7	9.8	12.4
Pain	PA	27.0	25.4	29.3	27.2	28.2	26.4	25.9	29.2	33.7	24.0	22.3	25.8	30.2	24.8	22.4	21.3	22.3	25.2	29.6
Dyspnoea	DY	21.0	21.1	20.3	17.1	20.4	22.1	23.1	21.7	23.4	17.4	16.1	18.5	20.7	16.2	17.1	17.1	15.8	18.6	20.8
Insomnia	SL	28.9	26.7	31.8	30.2	30.1	28.6	26.4	28.5	33.6	30.5	27.9	34.5	33.8	30.6	29.8	29.4	29.8	30.5	32.2
Appetite loss	AP	21.1	19.2	23.8	19.7	20.8	21.0	22.4	20.8	28.2	19.1	16.4	23.2	21.9	17.5	18.3	20.3	14.3	22.6	32.3
Constipation	CO	17.5	16.2	19.9	15.3	15.1	17.7	21.7	17.0	23.2	15.8	14.3	18.8	17.7	12.3	15.8	18.6	16.2	16.3	20.4
Diarrhoea	DI	9.0	8.7	9.3	9.0	8.9	9.2	8.9	8.3	10.7	16.6	15.8	16.7	18.5	16.1	15.6	17.7	23.8	15.4	11.1
Financial difficulties	FI	16.3	15.6	17.5	23.6	20.1	13.8	8.5	15.4	16.2	13.6	14.4	12.2	23.8	16.2	11.8	6.7	10.8	13.8	21.9

European and country-specific normative QLQ-C30 reference values																						
Variable		Normative population																				
Europe		All	Male							Female							Country					
			All	18-29	30-39	40-49	50-59	60-69	≥70	All	18-29	30-39	40-49	50-59	60-69	≥70	DE 2001	DE 2014	NL 2011	NO 1998	SE 2000	SE 2012
Global health status	QL	75.7	77.3	82.3	79.5	77.0	76.0	74.8	70.6	74.1	78.3	77.9	75.1	74.5	72.3	66.6	71.5	75.9	77.9	75.1	76.4	75.5
Physical functioning	PF	91.0	93.2	97.8	97.3	94.8	93.0	89.9	81.0	88.9	96.0	95.0	92.8	89.8	85.0	74.4	90.5	92.7	91.3	88.9	90.3	88.9
Role functioning	RF	88.1	89.8	94.4	93.2	90.3	88.5	87.3	80.5	86.5	92.6	90.2	89.0	86.7	84.0	76.6	88.5	90.8	89.7	82.8	86.8	87.9
Emotional functioning	EF	83.2	85.4	87.6	84.8	83.1	84.2	86.1	86.6	81.0	81.2	80.6	79.2	82.0	82.5	81.6	79.2	83.7	89.3	82.6	80.7	83.2
Cognitive functioning	CF	90.5	91.2	94.4	93.3	91.6	91.3	89.0	84.1	89.8	93.1	91.9	90.5	90.0	89.7	84.4	91.5	93.8	93.0	86.2	88.3	87.7
Social functioning	SF	91.5	92.6	96.6	93.9	92.7	91.2	90.1	87.8	90.5	93.9	91.0	91.7	89.5	90.6	86.7	91.5	93.6	94.7	85.3	91.9	90.4
Fatigue	FA	19.5	16.8	13.5	14.9	16.1	17.0	17.4	24.4	22.2	19.4	20.4	20.3	20.5	22.0	29.8	16.6	15.0	17.0	29.0	21.2	19.1
Nausea and vomiting	NV	3.1	2.3	1.9	2.6	2.3	2.3	2.1	2.5	3.8	4.6	3.7	3.4	2.8	3.0	5.1	2.7	2.1	3.1	4.1	3.5	2.8
Pain	PA	16.5	13.9	7.2	9.7	13.6	16.7	17.9	22.7	19.0	11.9	13.7	17.1	19.7	24.9	28.5	14.6	15.8	13.9	20.9	18.1	19.0
Dyspnoea	DY	11.1	10.8	8.3	6.5	9.5	10.5	13.0	20.5	11.3	8.5	7.4	9.4	9.4	12.8	20.4	7.8	6.9	6.6	14.4	18.2	14.9
Insomnia	SL	15.7	12.6	8.3	9.6	13.5	14.2	15.4	17.2	18.7	11.7	13.4	17.1	19.9	23.8	27.9	15.5	11.7	13.5	20.8	17.1	18.7
Appetite loss	AP	4.8	3.8	4.0	3.5	3.5	3.4	3.3	5.1	5.8	6.6	5.3	4.6	3.8	4.7	8.9	5.2	3.6	3.3	7.7	4.7	3.6
Constipation	CO	5.2	3.2	1.7	1.7	2.3	3.3	4.0	8.2	7.0	5.2	5.5	5.5	6.9	8.3	10.9	3.4	2.1	4.8	11.1	4.8	5.5
Diarrhoea	DI	4.9	4.7	3.7	3.8	5.9	5.7	4.3	4.9	5.1	5.0	5.3	5.3	4.8	4.7	5.2	2.9	2.5	3.8	9.5	5.4	5.8
Financial difficulties	FI	5.7	5.0	2.4	4.6	5.4	6.5	6.6	5.5	6.3	3.3	5.4	6.7	7.4	6.3	8.5	5.6	4.7	2.9	9.3	6.7	5.1

European cancer QLQ-C30 reference values													
Variable		Cancer											
Europe		All	Age		Gender		Neoadjuvant		Metastases		WHO status		Colorectal
			≤60	>60	Male	Female	No	Yes	No	Yes	Good	Bad	
Global health status	QL	63	65	61	65	61	64	58	67	58	64	44	60
Physical functioning	PF	81	84	76	82	78	82	75	86	74	83	50	77
Role functioning	RF	69	69	68	70	67	70	62	74	63	71	39	66
Emotional functioning	EF	71	71	71	73	68	72	68	73	67	72	62	68
Cognitive functioning	CF	86	87	84	87	84	87	79	89	85	87	72	87
Social functioning	SF	77	77	77	78	75	78	70	81	73	78	56	75
Fatigue	FA	31	29	35	29	34	30	38	25	39	29	57	37
Nausea and vomiting	NV	6	5	7	5	7	6	7	4	9	5	15	7
Pain	PA	24	23	27	24	25	23	29	21	33	23	46	25
Dyspnoea	DY	15	13	18	15	14	15	17	13	21	15	24	17
Insomnia	SL	27	26	28	25	29	26	32	23	34	26	39	31
Appetite loss	AP	16	13	21	15	18	16	18	11	26	14	38	23
Constipation	CO	13	10	18	12	14	12	18	9	19	12	29	16
Diarrhoea	DI	6	6	7	7	6	6	7	5	9	6	8	12
Financial difficulties	FI	15	17	9	15	14	14	15	14	13	14	21	13

EORTC QLQ-LMC21

The EORTC QLQ-LMC21 is meant for use among patients with colorectal liver metastases varying in disease stage and treatment modality (i.e. surgery, chemotherapy, radiotherapy, etc.). The QLQ-LMC21 is composed of a symptom scale containing 13 items, each ranging in score from 0-100.

The principle for scoring the scale (either directly or via provided matrices) is the same in all cases:

1. Estimate the average of the items that contribute to the scale; this is the *raw score*.
2. Use a linear transformation to standardise the raw score, so that scores range from 0 to 100; a higher score represents a higher ('better') level of functioning, or a higher ('worse') level of symptoms.

**Calculating the EORTC QLQ-LMC21 score**
In practical terms, if questions Q_1, Q_2, … Q_n are included in a scale, the procedure is as follows:<br><br>$\text{Raw score} = \frac{Q_1 + Q_1 + \ldots + Q_n}{n}$<br><br>To obtain the score *S*, a linear transformation to 0-100 is applied:<br><br>Symptom scale: $S_{symptoms} = \left\{\frac{(\text{Raw score} - 1)}{\text{range}}\right\} \times 100$<br><br>$\text{Range} = \text{Raw score}_{maximum} - \text{Raw score}_{minimum}$<br><br>The range is the same in all items, as these are scored 1 to 4, giving a range = 3.<br><br>The EORTC provides a validated manual (Quality of Life Unit, EORTC Data Center, Avenue E. Mounier 83 - B11, 1200 Brussels, Belgium) with standardised SPSS syntax, which includes missing data imputation, to calculate the scales. The data in this trial will be calculated and analysed in accordance with this manual.[23]

**Scoring the QLQ-LMC21**				
	**Scale**	**Number of items**	**Item range**	**Item numbers**
**Symptom scale**				
**Eating**	LMNutri	2	3	31, 32
**Activity / vigour**	LMCactVig	3	3	37, 43, 44
**Pain**	LMCPa	3	3	39, 40, 42
**Emotional problems**	LMCEp	4	3	47, 48, 49 ,50
**Weight loss**	LLMCWL	1	3	33
**Taste**	LMCTA	1	3	34
**Dry mouth**	LMCDM	1	3	35
**Sore mouth / tongue**	LMCSM	1	3	36
**Peripheral neuropathy**	LMCPN	1	3	38
**Jaundice**	LMCJ	1	3	41
**Contact with friends**	LMCFr	1	3	45
**Talking about feelings**	LMCFeelings	1	3	46
**Sex life**	LMCSx	1	3	51

Calculating the EORTC QLQ-LMC21 scales
**Symptom scale**

$$\text{Eating} = \left\{ \frac{\left(\left(\frac{Q_{31} + Q_{32}}{2} \right) - 1 \right)}{(4-1)} \right\} \times 100$$

$$\text{Activity/vigour} = \left\{ \frac{\left(\left(\frac{Q_{37} + Q_{43} + Q_{44}}{3} \right) - 1 \right)}{(4-1)} \right\} \times 100$$

$$\text{Pain} = \left\{ \frac{\left(\left(\frac{Q_{39} + Q_{40} + Q_{42}}{3} \right) - 1 \right)}{(4-1)} \right\} \times 100$$

$$\text{Emotional problems} = \left\{ \frac{\left(\left(\frac{Q_{47} + Q_{48} + Q_{49} + Q_{50}}{4} \right) - 1 \right)}{(4-1)} \right\} \times 100$$

$$\text{Weight loss} = \left\{ \frac{\left(\left(\frac{Q_{33}}{1} \right) - 1 \right)}{(4-1)} \right\} \times 100$$

$$\text{Taste} = \left\{ \frac{\left(\left(\frac{Q_{34}}{1} \right) - 1 \right)}{(4-1)} \right\} \times 100$$

$$\text{Dry mouth} = \left\{ \frac{\left(\left(\frac{Q_{35}}{1} \right) - 1 \right)}{(4-1)} \right\} \times 100$$

$$\text{Sore mouth / tongue} = \left\{ \frac{\left(\left(\frac{Q_{36}}{1} \right) - 1 \right)}{(4-1)} \right\} \times 100$$

$$\text{Peripheral neuropathy} = \left\{ \frac{\left(\left(\frac{Q_{38}}{1} \right) - 1 \right)}{(4-1)} \right\} \times 100$$

$$\text{Jaundice} = \left\{\frac{\left(\left(\frac{Q_{41}}{1}\right) - 1\right)}{(4-1)}\right\} \times 100$$

$$\text{Contact with friends} = \left\{\frac{\left(\left(\frac{Q_{45}}{1}\right) - 1\right)}{(4-1)}\right\} \times 100$$

$$\text{Talking about feelings} = \left\{\frac{\left(\left(\frac{Q_{46}}{1}\right) - 1\right)}{(4-1)}\right\} \times 100$$

$$\text{Sex life} = \left\{\frac{\left(\left(\frac{Q_{51}}{1}\right) - 1\right)}{(4-1)}\right\} \times 100$$

Presenting the EORTC QLQ-LMC21 scores

The EORTC QLQ-LMC21 summary score is provided in numbers ranging from 0-100. In addition to the summary score, all EORTC QLQ-LMC21 scale scores are also provided. Between-treatment arms analyses are performed. In addition, the QLQ-LMC21 scores may be compared with several QLQ-LMC21 ‘reference values’.[14,27-32]

Hepatectomy QLQ-C30/LMC21 reference values											
Variable		Hepatectomy		Hepatectomy	Hepatectomy	Hepatectomy		Hepatectomy		After hepatectomy	
		Before	After	Before	After	Before	After	Before	After	Benign	Malignant
Global health status	QL	76	69	76.3	62	80	73	78	86	71.7	65.4
Physical functioning	PF	88	81	88.0	75	89	79	93	89	70.8	66.6
Role functioning	RF	82	70	80.7	63	84	71	75	92	67.6	68.2
Emotional functioning	EF	79	80	79.8	70	73	75	82	94	78.3	62.7
Cognitive functioning	CF	87	85	87.2	78	84	80	82	94	80.3	61.6
Social functioning	SF	79	75	78.5	66	75	69	91	92	76.8	63.5
Fatigue	FA	21	33	21.8	39	26	25	6	3	55.6	74.4
Nausea and vomiting	NV	3	8	3.4	8	5	38	6	3	63.9	70.1
Pain	PA	12	22	12.2	22	14	12	3	3	62.4	78.8
Eating	NU	8	12	7.6	12	10	16	0	0	70.8	66.6
Activity/vigour	AV	29	41	29.6	43	28	40	14	8	59.6	72.4
Pain	PA2	14	21	14.4	19	15	23	3	5	63.3	77.2
Emotional problems	EP	30	33	19.4	40	44	32	28	3	60.0	71.3
Weight loss	WL	6	7	11.6	13	14	11	3	5	63.0	70.6
Taste	TA	8	14	34.6	14	6	13	0	3	59.3	71.6
Dry mouth	DM	16	18	6.8	27	12	15	0	3	62.5	70.0
Sore mouth / tongue	SM	6	10	8.5	12	4	7	3	0	67.0	68.5
Peripheral neuropathy	PN	19	28	15.8	41	14	23	9	11	58.0	72.3
Jaundice	J	1	3	5.5	4	6	7	3	3	66.4	68.9
Contact with friends	FR	11	14	10.2	14	18	15	8	3	-	-
Talking about feelings	FE	11	11	31.1	12	13	13	8	0	-	-
Sex life	SX	34	42	30.6	46	41	40	29	30	-	-

Body image

The body image questionnaire (BIQ) consists of eight items evaluating body image and cosmesis after surgery. The body image scale measures patients' perception of and satisfaction with their own body and explores patients' attitudes toward their bodily appearance (items 1, 2, 3, 4, 5). The cosmetic scale assesses the degree of satisfaction of patients with respect to the physical appearance of the scar (items 6-8).[33,34] Body image results are provided in numbers ranging from 5-20 and cosmesis results are provided in numbers ranging from 3-24.

Scoring the body image questionnaire				
	Number of items	Minimum score	Maximum score	Item numbers
**Body image**	5	5	20	1, 2, 3, 4, 5
**Cosmesis**	3	3	24	6, 7, 8

Calculating the Body Image score
$\text{Body image} = \text{Sum Body Image Questions}_{1-8}$

Delay in discharge after functional recovery and reasons

Delay in discharge after functional recovery is the difference in time between hospital length of stay and time to functional recovery and can be measured by subtracting the time to functional recovery from the hospital length of stay. Delay in discharge after functional recovery is measured in days.

Calculating the discharge delay
$\text{Discharge delay after functional recovery (days)} = \text{Hospital lenghtof stay} - \text{Time to functional recovery}$

Reasons for delay in discharge after functional recovery are provided descriptively, in numbers and as percentages of the total number of patients. These are categorised accordingly:

Reasons for delay in discharge categories	
**1**	Awaiting test results
**2**	In-hospital administrative reason(s)
**3**	Post-hospital reason(s)(e.g. home care arrangements)
**4**	Patient feels not ready
**5**	Patient transport
**6**	Other reason(s)

12-Month incidence of incisional hernia
Incisional hernia, or cicatricial hernia, is defined as any abdominal wall gap with or without a bulge in the area of a postoperative scar perceptible or palpable by clinical examination or imaging. At 12 months after surgery (regular control), examination for incisional herniation is performed by clinical examination, computer tomography (CT) or additional ultrasound (US) examination. Incisional hernia incidence is provided in numbers and as percentage of the total number of patients.
12-Month hospital and societal costs
Costs for open and laparoscopic liver surgery are measured in Euro's (€). Conversion to the year most data were collected (index year) in amount of Euro's needs correction using the country's harmonised inflation index for the month the expenses were made. This is done using an inflation calculator which can also use other types of currencies (https://www.inflationtool.com).

Treatment costs can be roughly divided into direct and indirect costs. Direct costs consist of expenses for operating theatre, material, personnel, diagnostics and medication. Unit prices are based on prices from the participating hospital financial departments or, if unavailable, are derived from national guidelines for cost pricing. Also, readmission costs, postoperative outpatient clinic visits, general practitioner consultations and home care related costs are added to the total hospital expenses. Indirect costs consist of productivity loss for patients performing paid labour and is calculated using the human-capital approach, which counts any hour not worked as an hour lost. In addition, the friction cost approach is also applied, which assumes that after a friction period of 3 months, the work missed by the patient is replaced by someone else and therefore no longer counts as extra costs.

**Treatment costs categories**	
**Direct costs**	Diagnostics<br>Operating theatre<br>Material<br>Personnel<br>Medication
**Indirect costs**	Productivity loss
**Other costs**	Readmission<br>Outpatient clinic visits<br>General practitioner consultations<br>Home care<br>Other care

Time to adjuvant chemotherapy initiation
In patients with malignant disease and adjuvant systemic treatment as standard of care, time to adjuvant chemotherapy initiation is defined as the difference in time in days between start of adjuvant chemotherapy and surgery. The decision to administer adjuvant chemotherapy is made by the multidisciplinary boards based on local guidelines. Time to start of adjuvant chemotherapy is measured in days.

Calculating time to adjuvant chemotherapy initiation
$\text{Time to adjuvant chemotherapy (days)} = (\text{Date}_{\text{start adjuvant chemotherapy}}) - (\text{Date}_{\text{surgery}})$

Overall five-year survival
Overall five-year survival is defined as the number of patients who are alive five years after surgery and is measured in numbers and percentages of the total number of patients.

If available, reasons for death are provided categorically. These may be:

Reasons of death categories	
**1**	Related to liver surgery
**2**	Due to progressive/metastatic disease
**3**	Due to any other reason
**4**	Unknown reason

Calculating overall five-year survival
$\text{Overall survival}_{5\ years\ after\ surgery} = \left(\frac{\text{Patients alive}_{\text{5 years after surgery}}}{\text{Total patients}}\right) \times 100$

1.3.3 Definition of safety outcomes
All relevant safety outcomes have been extensively described as secondary outcome:

- Conversion
- Unfavourable intraoperative incident
- Adverse event
- Serious adverse event; including hospital admission over 10 days, readmission within 30 days, life-threatening event (Intensive Care admission), serious invalidity and 90-day mortality.
- Composite endpoint of morbidity after liver surgery
- Resection margin

1.4 Summary of protocol amendments
Since the ORANGE II PLUS trial protocol (version 7) was originally approved by the Maastricht University Medical Ethics Committee on 7 August 2013, there have been a number of protocol amendments. The most important amendments (thus excluding minor textual and formatting changes) are summarised. Since the

ORANGE SEGMENTS trial protocol (version 12) was approved by the Maastricht Medical Ethics Committee on 18 May 2017, there have been no additional protocol amendments.

Version 8 – 6 June 2013.

- Textual revision of the descriptive and inferential statistics (paragraphs 10.1, 10.2 and 10.3).
- Addition of the Data and Safety Monitoring Board as part of the monitoring and quality assurance of the trial (paragraph 12.2).

Version 9 – 8 July 2014.

- Addition of a risk analysis of the trial (paragraph 13).

Version 10 – 4 August 2014.

- Amendment of the inclusion criterium for age: removal of the 80-year upper age limit.

Version 11 – 29 January 2015.

- Amendment of the inclusion criterium for surgery indication: a maximum of one additional resection (wedge or metastasectomy), thus in addition to the hemihepatectomy, is also allowed.

Version 12 – 30 March 2017.

- Extension of the sample size from 250 to 350 patients for the ORANGE II PLUS trial.
- Extension of the anticipated number of participating centres from 5 to 15 for the ORANGE II PLUS trial.
- Addition of a second trial; the ORANGE SEGMENTS trial, which is to be completed and published separately from the ORANGE II PLUS hemihepatectomy trial. In essence, the addition of two extra arms for open and laparoscopic parenchymal preserving resections (including wedge resections and full segmentectomies) of the posterosuperior liver segments (4a, 6/7, 7, 8).

SECTION 2 DESIGN ISSUES

2.1 Data collection and follow-up

Preoperative values that are required for establishment and evaluation of the primary and secondary outcome are established before surgery (baseline measurements). Next, data is gathered during surgery, while the patient is admitted and when the patient is discharged. Follow-up moments for data collection are planned at 10 days, 3, 6, 12 months and 5 years after surgery. Before closure of either the OpenClinica or Castor database, any missing data is requested per site.

All data is gathered in accordance with the European General Data Protection Regulation (EU GDPR), using the patient's trial code (a pseudo-anonymisation which was automatically created by the ALEA randomisation software) to ensure patient confidentiality.

ORANGE II PLUS - OpenClinica

All patient data are prospectively collected by trial sites on paper case record forms (CRFs). Follow-up data is gathered directly at the outpatient clinic, sent by post or email or collected telephonically. CRFs are digitised into the community edition of OpenClinica® (Ikaza Research, Cambridge, Massachusets, USA). OpenClinica is an open source clinical trial software platform for electronic data capture (EDC) and is validated and stored in compliance with good clinical practice (GCP) guidelines. OpenClinica is provided by the Dutch Research IT (TraIT) project. The OpenClinica data is stored in a secured Oracle® database (Oracle Corporation, Redwood Shores, CA, United States of America) that is hosted by Vancis. Vancis possesses the Dutch NICTIZ certificate and meets the ISO 27001 standard for information security management. The network communication with the TraIT OpenClinica web-application is secured via the HTTPS protocol. They have a Trust Provider B.V. TLS RSA CA G1 certificate that guarantees that the TraIT OpenClinica user is communicating with the TraIT OpenClinica environment. The OpenClinica database is backed up every night and every other year a disaster recovery test is performed.

ORANGE SEGMENTS - Castor

All patient data are prospectively collected on paper case record forms or digitally. Follow-up data is entered digitally by the patient through an email link or is gathered directly at the outpatient clinic, sent by post, email or collected telephonically. ORANGE SEGMENTS CRFs are digitised into Castor EDC (Castor Electronic Data Capture, Ciwit B.V., Amsterdam, The Netherlands). Castor EDC is a commercial clinical trial software platform for electronic data capture and is validated and stored in compliance with good clinical practice guidelines. Castor EDC is fully ISO 27001, 27002 and 9001 certified.

2.2 Study design

The ORANGE II PLUS trial and ORANGE SEGMENTS trial are both international and multicentre superiority studies with a prospective, double blind, randomised controlled design. Each trial produces two treatment arms in a 1:1 ratio. For overview, both trials are summarised into a single trial protocol flowchart. Note that the sample size for the ORANGE II PLUS trial was extended at interim analysis.

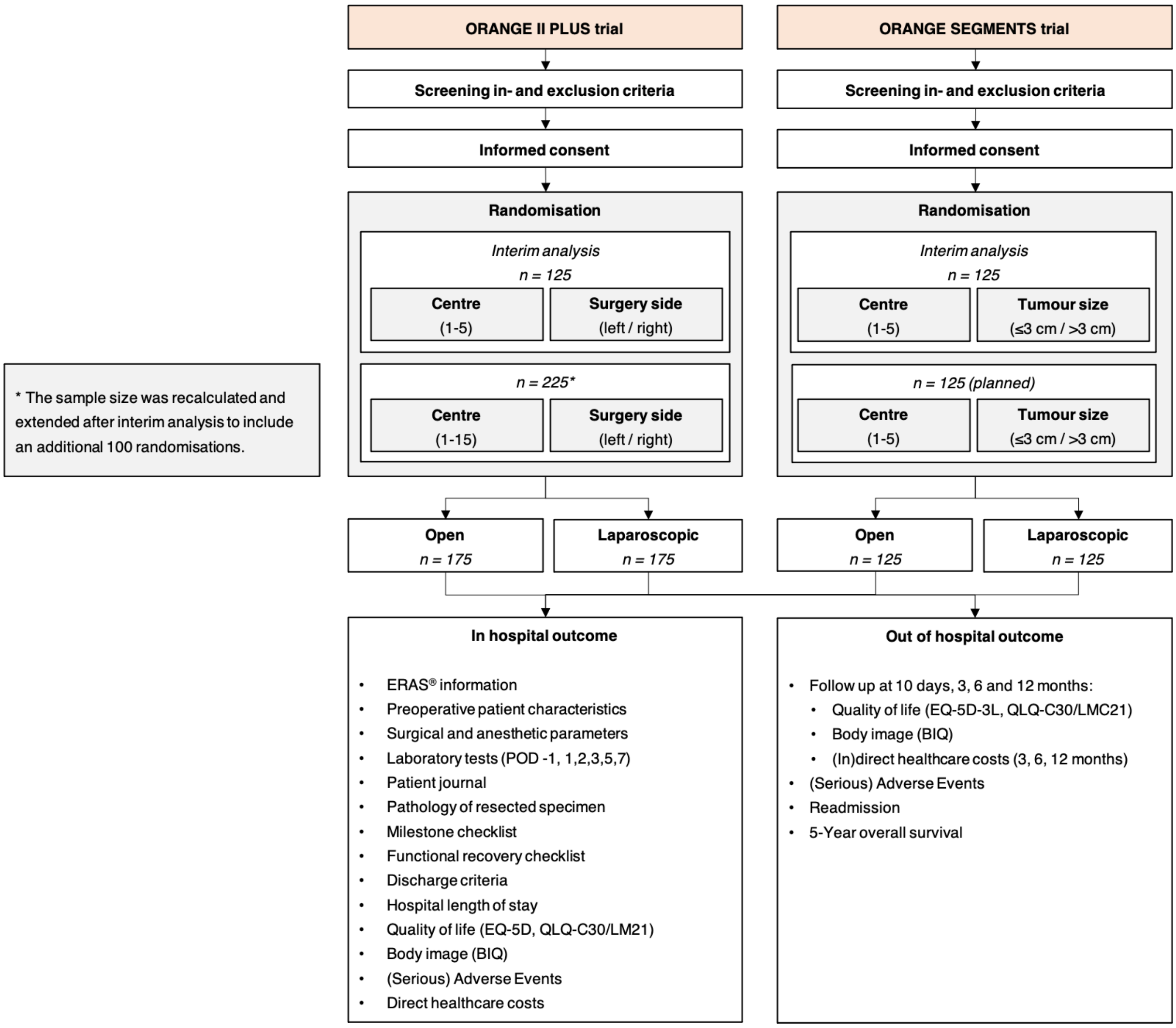

2.3 Treatment allocation/ randomisation

Treatment is allocated using internet-based randomisation software (ALEA®, online randomisation module, hosted by the Clinical Trial Center Maastricht). Minimisation is applied to remain balance among both arms for both hemihepatectomy sides (ORANGE II PLUS) or tumour sizes (ORANGE SEGMENTS) and within each treatment centre. Normal allocation chance is set to 50% (1:1). Only after an imbalance of 2 patients within a given treatment centre*side factor (ORANGE II PLUS) or treatment centre*tumour size factor (ORANGE SEGMENTS), the probability to be assigned to the arm with the least number of patients is set to 90%. After restoration of the balance (thus an imbalance of less than 2 patients within a given treatment centre*side factor or treatment centre*tumour size factor), the allocation chance is reset to 50%. For each patient, individual allocation chance (90:10 or 50:50 or 10:90) is recorded to be included as a covariate into the outcome analyses. The randomisation procedure is carried out online by the research nurse or principal investigator per centre.

2.4 Blinding

The patient, ward physicians and nurses are blinded to the type of intervention using a large abdominal dressing that covers the surgical incisions until postoperative day 4 (ORANGE II PLUS) or until functional recovery has been established (ORANGE SEGMENTS). Only if functional recovery has been achieved before postoperative day 4, blinding may be removed. In addition to physical blinding, the operating schedules and surgical reports are also adjusted by the principal investigators to prevent any preliminary unblinding before day 4 or before functional recovery.

The medical and nursing staff can be unblinded if this is necessitated by patients' condition, for instance because of severe wound leakage, infection or any other adverse event that requires immediate medical attention. All unblindings and reasons for unblinding are registered. Blinding adequacy is assessed by asking the patient the second day after surgery whether open or laparoscopic surgery has been performed. Theoretically, a 50% correct overall study score should be obtained if blinding was perfectly executed in all patients. Obviously, the operating surgeon(s), assisting residents, surgery assistants, anaesthesiologist(s) and other staff directly involved in the surgical procedure cannot be blinded.

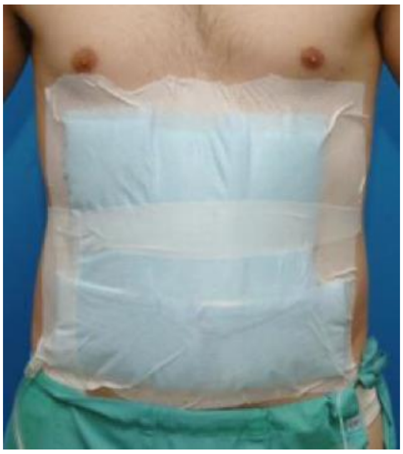

2.5 Study power and sample size

The estimated sample sizes for these parallel group superiority trials are based on the primary outcome variable. Unfortunately, data on time to functional recovery in liver surgery is scarce.. In absence of data on time to functional recovery, hospital length of stay (LOS) is used instead of time to functional recovery to calculate the required sample size. It has been consistently shown that time to functional recovery is approximately 2 days less than LOS for most patients and for both treatment arms. [1,33]

Laparoscopic surgery (for either hemihepatectomy or parenchymal preserving posterosuperior liver segment resection) is hypothesised to shorten time to functional recovery with 2 days in comparison to patients undergoing the open procedure. Assuming a standard deviation (SD) of 5.0 days for time to functional recovery, the implied effect size *d* becomes 0.40 (2/5). This effect size estimate is based on reports that showed that mean LOS and SD for laparoscopic hemihepatectomy were 8.5 and 5 days and mean LOS for open hemihepatectomy was 11.1 days and on reports that showed that the median LOS and range for laparoscopic parenchymal preserving posterosuperior liver segment resection were 4 days (1-11 days) and for open parenchymal preserving posterosuperior liver segment resection were 6 days (3-44 days). [34-37]

Taking into account a drop-out rate of 10% and a loss in degrees of freedom for estimating covariate effects (5 participating centres, hemihepatectomy side or tumour size), a total sample size of 250 patients (125 per arm) is planned to demonstrate a 2-day reduction in time to functional recovery with a two-sided 4% level of significance and a power of 80%. A two-sided 4% level of significance is used instead of 5% in view of the planned interim analysis halfway the trial with a two-sided 1% level of significance, thus preserving an overall type I error rate of 5%.[38]

Sample size calculations
The sample size calculation for a parallel group superiority trial is as follows.[39]<br><br>$$n = \frac{(r+1)\left(Z_{1-\alpha/2} + Z_{1-\beta}\right)^2 \sigma^2}{rd^2}$$<br><br>r is the allocation ratio<br>$Z_{1-\alpha/2}$ the type 1 error for a two-tailed test<br>$Z_{1-\beta}$ = the type 2 error<br>σ = the population variance<br>d = the effect size

Sample size calculations ORANGE II PLUS and ORANGE SEGMENTS trial
For an allocation ratio of 1:1, an alpha of 0.04, a power of 80%, a population standard deviation of 5 days and a population mean difference of 2 days, this means:<br><br>$$n = \frac{(r+1)\left(Z_{1-\alpha/2} + Z_{1-\beta}\right)^2 \sigma^2}{rd^2} = \frac{(1+1)(2.0537+0.84)^2 5^2}{1 \times 2^2} = \frac{2 \times 2.8937^2 \times 25}{4} = \frac{417.605}{4} \approx 105$$<br><br>This gives a sample size of 105 per treatment arm.<br><br>$n_{Total} = 105 \times 2 = 210$<br><br>• Corrections for loss in degrees of freedom (fixed centre effects, covariate effects):<br><br>Per treatment arm 4 patients for the loss due to centre effects (5 centres)<br>Per treatment arm 1 patient for the loss of a degree of freedom for the covariate surgery side.<br><br>This gives 220 patients in total. Then multiply with (100/90) to compensate power loss due to 10% dropout, giving total n = 244.<br><br>This number is rounded to **250 patients in total** and thus **125 patients per arm.**

Sample size extension ORANGE II PLUS trial
At interim analysis of the ORANGE II PLUS trial, more centres participated than was predicted and the effect size was lower than anticipated (0.34 instead of 0.40). The sample size was recalculated and the sample size was extended accordingly. This unplanned sample size extension will be corrected for using a combinatory analysis to prevent a type 1 error inflation. The sample size of the ORANGE SEGMENTS has <u>not</u> been extended.<br><br>For an allocation ratio of 1:1, an alpha of 0.04, a power of 80%, an effect size of 0.34, this means:<br><br>$$n = \frac{(r+1)\left(Z_{1-\alpha/2} + Z_{1-\beta}\right)^2 \sigma^2}{rd^2} = (1+1)(2.0537+0.84)^2 \times \left(\frac{1}{0{,}34}\right)^2 = 16.75 \times 8.65^2 \approx 145$$<br><br>This gives a sample size of 145 per treatment arm.<br><br>$n_{Total} = 145 \times 2 = 290$

- Corrections for loss in degrees of freedom due to extra centres (15 instead of 5 centres, so per treatment arm 10 extra patients:

$$n_{\text{Total}} = 290 + 20 = 310$$

- 10% drop-out

$$n_{\text{Total}} = 310 \times \left(\frac{100}{90}\right) = 345$$

The adapted sample size is rounded to **350 patients in total.**

2.6 Interim analyses and stopping guidance

Interim analysis of the primary outcome and mortality is performed once and is performed without disclosing treatment label after inclusion of 50% of the intention-to-treat sample (n = 125), applying two stopping rules:

Stopping for significance

The trial is stopped if interim analysis shows a significant difference between both trial arms with respect to the primary outcome, time to functional recovery, at a two-tailed alpha of 0.01. Otherwise, the trial is continued.

Stopping for safety

The trial is stopped if the mortality after hepatectomy (either hemihepatectomy or parenchymal preserving poster-superior liver segment resection) exceeds 5% in patients with normal liver function or exceeds 10% in cirrhotic patients.

2.7 Timing of analyses

All primary and secondary outcomes are to be analysed only after official approval of this statistical analysis plan by the principal investigators, coordinating investigators and statistician. Data analyses can only be performed after data digitisation (from paper to either OpenClinica or Castor database), data completeness evaluations, missing data retrieval and data cleaning have been completed. This is to be completed ultimately 2 years after surgery of the last randomised patient. Not all secondary outcomes can be analysed directly. These will be analysed in order of importance and relevance, depending on the availability of statistical support.

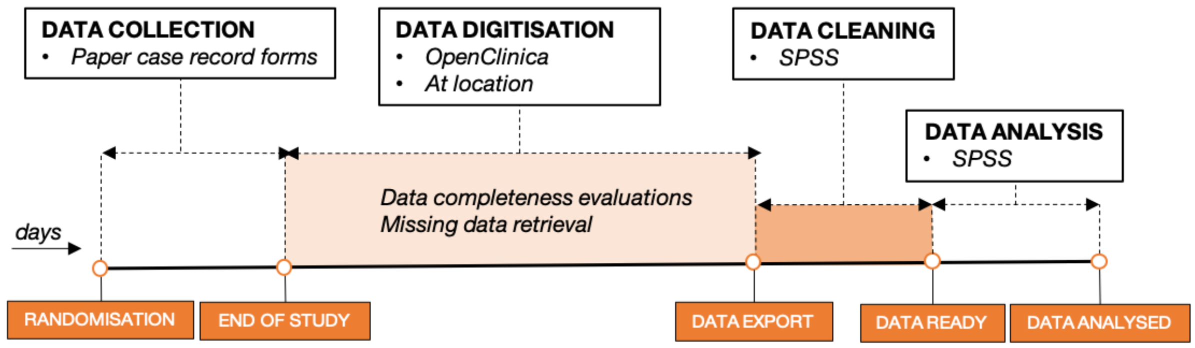

2.8 Protocol deviations

Protocol deviations are graded as major (with great impact on data quality or patient safety) or minor (lesser impact on data quality or patient safety). Major protocol deviations are also commonly known as protocol violations.

Major protocol deviations ('protocol violation')

Non-adherence

Non-adherence as a major protocol deviation may be for not complying to in- and exclusion criteria and not following the trial intervention (procedure and procedure allocation). Non-adherence to the trial intervention may be cancellation of surgery by the consultant between randomisation and surgery (e.g. because of extrahepatic metastases or hepatic tumours that were not visible on previous imaging) or intraoperatively (e.g. open-closed procedure for peritoneal metastases).

Other major protocol deviations

Any protocol deviation with major impact on patient safety or data quality that was not foreseen.

Minor protocol deviations

Unblinding

Uncovering of the treatment allocation to either the patient or the ward personnel before postoperative day 4 or functional recovery. Only if functional recovery has been achieved before postoperative day 4, blinding may be removed. Unblindings may be partial (for instance only the ward staff) or may be total (patient and ward staff). Unblindings may be accidental or may be necessitated (e.g. removal of the surgical dressing due to wound complications).

Other minor other protocol deviations.

Any protocol deviation with minor impact on patient safety or data quality that was not foreseen.

**Protocol deviation categories**	
**Major protocol deviations**	Non-adherence<br>Other major protocol deviation
**Minor protocol deviations**	Unblinding<br>Other minor protocol deviations

**Calculating protocol deviations**
$\% \text{ Major protocol deviations} = \left(\frac{\#\text{ major protocol deviations}}{\#\text{ total randomisations}}\right) \times 100$<br>$\% \text{ Minor protocol deviations} = \left(\frac{\#\text{ minor protocol deviations}}{\#\text{ total randomisations}}\right) \times 100$

SECTION 3 STATISTICAL ANALYSES

3.1 Definition of analysis populations

3.1.1 Intention-to-treat analyses

The intention-to-treat analyses include all randomised patients, according to the treatment they were allocated to. This also includes patients who did not actually receive the intended treatment, but who had undergone another type of resection. These analyses exclude patients who or for whom:

Intention-to-treat analysis exclusions	
**1**	Withdrew informed consent.*
**2**	No study data was available after randomisation.**

* All data that was collected until withdrawal of the informed consent by the patient will be used.
** The outcomes TFR and LOS cannot be measured at baseline by definition, and so, without post-randomisation data, the patient cannot be included into the analysis.

All patients who were excluded for the intention-to-treat analysis for these reasons will be shown in the CONSORT flowchart for both treatment arms.

3.1.2 Per-protocol analyses

The per-protocol analysis includes all randomised patients, according to the treatment they were allocated to and received. This analysis excludes patients who or for whom:

Per-protocol analysis exclusions	
**1**	Withdrew informed consent.*
**2**	The surgical procedure was cancelled pre- or intraoperatively.
**3**	The surgical procedure was altered pre- or intraoperatively.<br><br>(Conversion are not considered an alteration of surgical procedure.)
**4**	The data is missing when lost to follow-up.
**5**	Had major protocol violations.

* All data that was collected until withdrawal of the informed consent by the patient will be used.

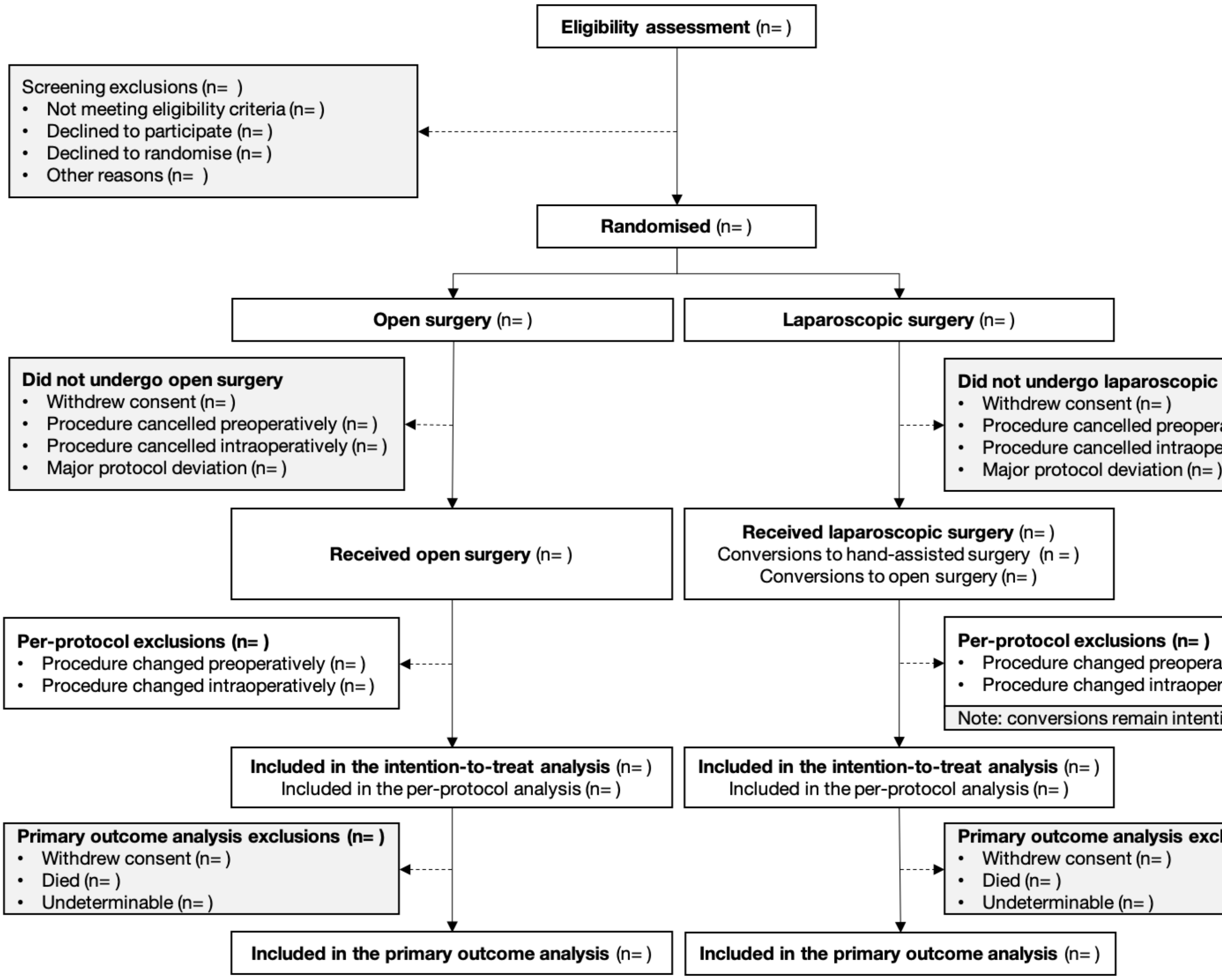

3.2 Analysis software

The statistical analyses are carried out using SPSS: IBM Corp. Released 2019. IBM SPSS Statistics for Windows, Version 27.0. Armonk, NY: IBM Corp.

3.3 Hypothesis testing

The ORANGE II PLUS and ORANGE SEGMENTS trials are both superiority trials. Laparoscopic liver surgery is hypothesised to do better in terms of time to functional recovery compared with open liver surgery. Furthermore, laparoscopy is also expected to do better in comparison to open surgery in terms of the secondary outcomes. Nevertheless, two-sided tests are performed for two reasons: First, to allow detection of a possible inferiority of laparoscopic liver surgery when compared with open liver surgery. Secondly, to maintain consistence between the significance testing procedure and the effect estimation with two-tailed confidence intervals.

3.3.1 Primary outcome

ORANGE II PLUS

For the primary outcome analysis, a two-stage combination procedure is used. The test statistic for the treatment effect at interim analysis (n=125) and the test statistic for the treatment effect based on the

remainder of the sample (n = 350-125 = 225) are combined into a single test statistic, using the combination rule described by Proschan.[40,41]. A two-sided 5% significance level is used.

Second, a naïve analysis of all 350 randomised patients is performed. A two-sided 4% significance level is used. The alpha of 0.04 compensates for the alpha of 0.01 used in the interim analysis, as originally planned.

For the ORANGE II PLUS trial, these two methods of analysis are used to crosscheck for the risk of type 1 error inflation arising from the unplanned sample size re-estimation and trial extension at interim analysis (see section 6 and 2.5).

ORANGE SEGMENTS
For the primary outcome analysis, a two-sided 4% significance level is used instead of a two-sided 5% significance level to control for the interim test (see section 2.6.1), which is performed at a two-sided 1% significance level.

3.3.2 Secondary outcomes
All secondary outcome analyses are performed at a two-sided 1% significance level .

3.4 Multivariable analyses

3.4.1 Continuous outcome variables
Fixed effect linear regression analysis is used for quantitative outcome comparison.

3.4.2 Dichotomous or categorical outcome variables
Fixed effect logistic regression analysis is used for binary outcome comparison and fixed effect multinomial logistic regression analyses for comparison of categorical outcome measures with more than two discrete outcomes.

3.4.3 Time-to-event outcome variables
Fixed effect Cox regression analysis is used to compare time-to-event outcomes. Reasons for and moments of censoring are:

**Reasons for and moments of censoring**		
	Reason for censoring	Moment of censoring
**1**	Event	Moment of event
**2**	Lost to follow-up	Last moment of follow-up
**3**	Study withdrawal	Moment of study withdrawal
**4**	End (of study) measurement	Last moment of measurement

3.4.4 Clustered data
Random (mixed) effect regression analyses (linear, logistic, and Cox) with centre as random effect (random intercept and random slope to allow for treatment by centre interaction) are done as additional analysis in view of the unplanned large number of centres.

3.5 Covariate adjustment

The main analyses (regression models) use the minimisation factors (centre and hemihepatectomy side for ORANGE II PLUS, centre and tumour size for ORANGE SEGMENTS) as covariates and also include age, sex, tumour type, and patient's individual allocation chance as covariates. In sensitivity analyses that aim to provide information on outcome robustness by repeating the analyses, only the minimisation factors as covariates will be used without any additional covariate adjustments.

3.5.1 Covariate adjustments for ORANGE II PLUS

1. Centre (dummy coding)
2. Hemihepactectomy side (left / right)
3. Age (years, continuous)
4. Sex (male, female)
5. Tumour type (benign/ malignant)
6. Patient's individual allocation chance (minimisation balance)

3.5.2 Covariate adjustment for ORANGE SEGMENTS

1. Centre (dummy coding)
2. Tumour size (≤3 cm / >3 cm)
3. Age (years, continuous)
4. Sex (male, female)
5. Tumour type (benign/malignant)
6. Patient's individual allocation chance (minimisation balance)

3.6 Interaction

Possible interactions are deleted before testing the treatment main effect. If significant interaction is found, subgroup analyses will be done:

- Per centre if there is treatment-centre interaction
- Per hemihepatectomy side if there is treatment- hemihepatectomy side interaction
- Per tumour size if there is a treatment-tumour size interaction (only for ORANGE SEGMENTS)
- Per sex if there is a treatment-sex interaction
- Per age category (young 18-59 years / middle-aged 60-74 years / old ≥75 years) if there is a treatment-age category interaction.
- Per tumour type if there is a treatment-tumour type interaction.

3.6.1 Interaction terms ORANGE II PLUS

1. Centre*treatment
2. Hemihepatectomy side*treatment
3. Age*treatment
4. Sex*treatment
5. Tumour type*treatment

3.6.2 Interaction terms ORANGE SEGMENTS

1. Centre*treatment
2. Tumour size*treatment
3. Age*treatment
4. Sex*treatment
5. Tumour type*treatment

3.7 Methods for handling withdrawals, missing data and outliers

3.7.1 Withdrawals

Patients may withdraw their consent to participate at any moment without providing any reason. Patient data that has been collected patient until the moment of withdrawal may be used, unless stated otherwise. Withdrawals are included in the CONSORT diagram.[42]

3.7.2 Missing data

In case of missing data, several methods will be considered to handle missing data depending on which variables are missing, the mechanism of incomplete data (i.e. Missing Completely At Random (MCAR), Missing At Random (MAR), and Missing Not At Random (MNAR)), and the missing rate. Complete Case Analyses can be considered if the percentage of missing values is low (e.g. <10%). Mean imputation can be used for covariate missingness.[43] For outcome missingness exceeding 10%, multiple imputation can be used.

3.8 Checking assumptions and data transformations

3.8.1 Normality

Normality is explored visually (frequency distributions, stem-and-leaf plots, P-P plots, Q-Q plots and kernel density plots) and is quantified with Shapiro-Wilk tests. Transformation (e.g. logarithmic transformation) may be needed in case of severely non-normally distributed quantitative outcome or in case of outliers.

3.8.2 Proportional hazards

Time-to-event distributions are graphically displayed using Kaplan-Meier plots. In addition, Cox-regression with a time-dependent covariate (T_COV) and Schoenfeld residuals tests are used to test whether the proportionality assumption holds.

3.9 Handling multi-centre/clustered data

3.9.1 Adjustments for clustering

Eleven extra centres entered in the course of the trial, which increased the number of participating centres to sixteen. This justifies a mixed effects regression analysis with centre as random effect on top of the planned fixed effects regression (using dummy coding for centre). However, since the sample size calculation for the primary outcome has always been based on fixed effects regression, the latter remains the primary analysis and the random effects regression analysis with random intercept and random slope (see section 3.4.4) serves as a sensitivity analysis to explore the consequences of treating the participating centres as a random sample of centres.

For the secondary outcomes, all analyses will also be performed using both fixed and random effects regression analyses with centre as a random effect.

3.10 Subgroup analyses

Two sets of subgroup analyses must be distinguished. Subgroup analyses based on covariates for which interaction with treatment will be tested as specified in section 3.6 and is limited to the primary outcome FR and the secondary outcome LOS.
Additional subgroup analyses may be performed on other covariates than those mentioned in 3.6 and/ or other outcomes than FR or LOS. For this second group of subgroup analyses see section 8.

3.10.1 Interaction subgroup analyses

Subgroup analyses will be performed to assess the outcome difference between the open and the laparoscopic treatment arms per subgroup based on the covariates specified in section 3.6. Unless significant treatment*covariate interaction is found in the analyses specified in section 3.6, these subgroup analyses have an exploratory status only. In view of the large number of tests performed, the report will state this number explicitly to caution against overinterpretations of what may be type I errors due to multiple

testing. In view of a potentially modest sample size per subgroup, confidence intervals will be provided on top of p-values to caution against overinterpretations of what may be type II errors.

For the subgroup analyses a minimum number of n = 30 patients (central limit theorem) would be required in each subgroup for the analyses to be performed, and even that will be too small for sufficient power unless the effect is very large. For the subgroup analyses, only the minimisation factors described in Section 2.3 will be used as covariates. Similar to the main analysis, time to functional recovery will be tested here with p=0.04 and LOS with p=0.01. For further detail please consult section 3.3.1.

SECTION 4 DISPOSITION OF PATIENTS AND FOLLOW-UP INFORMATION

Preoperative values of the primary and secondary outcomes are established before surgery. Next, data is gathered during surgery, while the patient is admitted and when the patient is discharged. Follow-up moments for data collection are planned at 10 days, 3, 6, 12 months and 5 years after surgery.

	STUDY PERIOD										
	Enrolment	Randomisation	Post-allocation								Close-out
**TIMEPOINT**	**Screening**	**>1 week post screening**	**Preoperative POD -1**	**Surgery (POD 0)**	**Admission**	**Discharge**	**Follow-up 10 days**	**Follow-up 3 months**	**Follow-up 6 months**	**Follow-up 12 months**	**Follow-up 5 years**
**ENROLLMENT**											
Eligibility screen	●										
Informed consent		●									
Randomisation		●									
**INTERVENTION**											
Arm 1				●							
Arm 2				●							
**ASSESSMENT**											
Functional recovery			●	—	●						
Hospital length of stay			●	—	—	●					
Intraoperative blood loss				●							
Operation time				●							
Time to adjuvant chemotherapy						●	—	—	—	●	
Readmission percentage						●	—	—	—	●	
(Liver specific) morbidity				●	—	—	—	—	—	●	
Quality of life			●			●	—	—	—	●	
Body image			●			●	—	—	—	●	
Reasons for delay of discharge						●					
Incisional herniations						●	—	—	—	●	
Hospital and societal costs				●	—	—	—	—	—	●	
Five-year survival											●

SECTION 5 DEMOGRAPHIC AND BASELINE CHARACTERISTICS

Baseline characteristics		
Characteristic	**Open surgery (n=)**	**Laparoscopic surgery (n=)**
Sex – no. (%)		
Male		
Female		
Missing		
Age – yr		
Missing		
American Society of Anesthesiologists classification – no. (%)		
I: healthy		
II: mild systemic disease		
III: severe systemic disease		
Missing		
Body-mass index – kg/m^2		
Missing		
World Health Organisation performance status – no. (%)		
0: asymptomatic, normal activity		
I: symptomatic, normal activity		
II: symptomatic, <50% bedridden		
III: symptomatic, >50% bedridden		
IV: 100% bedridden		
Missing		
Previous abdominal surgery – no. (%)		
Missing		
Coexisting conditions – no. (%)		
Angina		
Class I: no limitation of physical activity		
Class II: mild limitation of physical activity		
Class III: marked limitation of physical activity		
Class IV: severe limitation of physical activity		
Myocardial infarction		
Cerebrovascular accident		

Hypertension		
Diabetes		
Type 1		
Type 2		
Chronic obstructive pulmonary disease		
Asthma		
Bronchitis		
Jaundice		
Other coexisting condition		
Missing		
Liver cirrhosis – no. (%)		
Child-Pugh A		
Child-Pugh B		
Child-Pugh C		
Missing		
Charlson Comorbidity Index		
Missing		
Diagnosis – no. (%)		
Colorectal metastasis		
Hepatocellular carcinoma		
Cholangiocarcinoma		
Haemangioma		
Adenoma		
Follicular nodular hyperplasia		
Other diagnosis		
Missing		
Preoperative chemotherapy – no.		
Missing		
Preoperative portal vein embolisation		
Missing		
Preoperative mobility		
Missing		
Preoperative Quality of life		
EQ-5D-3L		

Missing		
QLQ-C30		
Missing		
LMC-21 score		
Missing		
Hemihepatectomy side – no. (%)*		
Left		
Right		
Tumour size – no. (%)**		
≤3 cm		
>3 cm		
Postero-superior liver segment – no. (%)**		
Segment 4A		
Segment 6/7		
Segment 7		
Segment 8		
Segments involved – no. (%)**		
One		
Two		
Additional metastasectomy required – no. (%)		
Missing		

SECTION 6 PRIMARY OUTCOME ANALYSIS

The primary outcome is **time to functional recovery** (time to functional recovery is defined in section 1.3.2). Analyses will be performed for the ITT-population (section 3.1.1) as well as the PP-population (section 3.1.2).

Prior to the model being fitted, normality of the outcome will be explored as described in section 3.8.1. If the normality assumption is valid, first, a minimally adjusted fixed effects linear regression model adjusted for centre and hemihepatectomy side (ORANGE II PLUS) or tumour size (ORANGE SEGMENTS) will be performed. The model results will be presented, including the sample size, the treatment effect estimate, its 96% confidence interval (CI), standard error, and p-value. Secondly, the fully adjusted model will be presented (section 3.5).

<u>ORANGE II PLUS</u>
The primary fixed effect regression (with dummy coding of centre) needs to be performed twice to crosscheck for the risk of type 1 error inflation arising from the unplanned sample size re-estimation and trial extension from 250 to 350 patients at interim analysis based on the interim estimate of the treatment difference.

First, a two-stage combination procedure is used. The test statistic for the treatment effect at interim analysis (n=125) and the test statistic for the treatment effect based on the remainder of the sample (n = 350-125 = 225) are combined into a single test statistic, using the combination rule described by Proschan.[40,41] Alpha is set to 0.05 two-sided.

Second, a naïve analysis of all 350 randomised patients is performed. Alpha is set to 0.04 two-sided. The alpha of 0.04 compensates for the alpha of 0.01 used in the interim analysis, as originally planned.

Results of both analyses will be presented, including the sample size, the treatment effect estimate, its confidence interval (CI, 96% for the planned analysis, 95% for the combination procedure), standard error, and p-value.

If the results of the combination analysis and naïve analysis agree with respect to the presence and direction of an outcome difference between both treatments, then there is strong evidence for such a difference, if only one of these two analyses shows a significant difference, then there is some evidence for a difference. Otherwise, there is no evidence for a difference.

<u>ORANGE SEGMENTS</u>
For ORANGE SEGMENTS, the sample size was not re-estimated and extended at interim analysis, so there is no risk for any type 1 error inflation.

<u>Sensitivity analyses</u>
For the primary outcome additional sensitivity analyses will be performed. The primary goal of the sensitivity analysis is to assess analysis robustness of the results. First, a sensitivity will be performed with excluding outliers. Values more than 1.5 interquartile ranges (IQRs) below the first quartile or above the third quartile are considered outliers. Secondly, in view of to the unplanned extension of the number of participating centres, a random (mixed) effect linear regression model will be performed. For the ORANGE II PLUS sensitivity analyses, the same two-stage procedure is required as for the fixed effect regression model described above. Sensitivity analyses will only be adjusted for the minimisation factors described in section 3.5.

SECTION 7 SECONDARY OUTCOME ANALYSES

7.1 Hospital length of stay

Hospital length of stay is measured in days (continuous) and defined as described in section 1.3.2. Length of hospital stay is the dependent variable. The model will contain the covariables described in section 3.5. The relationship will be assessed using a random effects linear regression model. The model results will be presented, including the sample size, the increment (ß) of the treatment effect, its 99% confidence interval (CI), standard error, and p-value. Prior to the model being fitted, normality assumption will be checked and handled as described in section 3.8.1. The analysis will be performed among the ITT and PP population.

<u>Sensitivity analyses</u>

For hospital length of stay additional sensitivity analyses will be performed. The primary goal of the sensitivity analysis is to assess analysis robustness of the results. First, a sensitivity will be performed with excluding outliers. Values more than 1.5 interquartile ranges (IQRs) below the first quartile or above the third quartile are considered outliers. To account for potential baseline imbalance due to the minimisation procedure (section 2.3), additional analyses will be performed with adjustment for allocation chance (90:10, 50:50, 10:90). Sensitivity analyses will only be adjusted for the minimisation factors described in section 3.5.

7.2 Intraoperative blood loss

Intraoperative blood loss is measured in millimetres (continuous) and defined as described in section 1.3.2. Intraoperative blood loss is the dependent variable. The model will contain the covariables described in section 3.5. The relationship will be assessed using a random effects linear regression model. The model results will be presented, including the sample size, the increment (ß) of the treatment effect, its 99% confidence interval (CI), standard error, and p-value. Prior to the model being fitted, normality assumption will be checked and handled as described in section 3.8.1. The analysis will be performed among the ITT and PP population.

7.3 Operation time

Operation time is measured in minutes (continuous) and defined as described in section 1.3.2. Operation time is the dependent variable. The model will contain the covariables described in section 3.5. The relationship will be assessed using a random effects linear regression model. The model results will be presented, including the sample size, the increment (ß) of the treatment effect, its 99% confidence interval (CI), standard error, and p-value. Prior to the model being fitted, normality assumption will be checked and handled as described in section 3.8.1. The analysis will be performed among the ITT and PP population.

7.4 Conversions

A conversion is measured dichotomously (yes/no) and categorically (none, urgent reason, non-urgent reason, unknown reason) and defined as described in section 1.3.2. Conversions is the dependent variable. The model will contain the covariables described in section 3.5. The relationship will be assessed using a random effects logistic regression model for the dichotomously outcome and using a random effects multinomial logistic regression model for the categorically outcome. The results will be presented, including the sample size, the Odds Ratio (exp(ß)) of the treatment effect, its 99% confidence interval (CI), standard error, and p-value. The analysis will be performed among the ITT and PP population.

7.5 Unfavourable intraoperative incidents

An unfavourable intraoperative incident is measured dichotomously (yes,no) and categorically (Satava grades; Grade I, II, and III) and defined as described in section 1.3.2. Unfavourable intraoperative incident is the dependent variable. The model will contain the covariables described in section 3.5. The relationship will be assessed using a random effects logistic regression model for the dichotomously outcome and using a random effects multinomial logistic regression model for the categorically outcome. The results will be

presented, including the sample size, the Odds Ratio (exp(ß)) of the treatment effect, its 99% confidence interval (CI), standard error, and p-value. The analysis will be performed among the ITT and PP population.

7.6 Postoperative morbidity – Accordion

The Accordion classification for postoperative morbidity is measured categorically (Grade I, II, III, IV, V, and VI) and defined as described in section 1.3.2. The Accordion classification for postoperative morbidity is the dependent variable. The model will contain the covariables described in section 3.5. The relationship will be assessed using a random effects multinomial logistic regression model. The results will be presented, including the sample size, the Odds Ratio (exp(ß)) of the treatment effect, its 99% confidence interval (CI), standard error, and p-value. The analysis will be performed among the ITT and PP population.

7.7 Postoperative morbidity – Clavien-Dindo

The Clavien-Dindo classification for postoperative morbidity is measured categorically (Grade I, II, III, IIIA, IIIB, IV, IVA, IVB, and V) and defined as described in section 1.3.2. The Clavien-Dindo classification for postoperative morbidity is the dependent variable. The model will contain the covariables described in section 3.5. The relationship will be assessed using a random effects multinomial logistic regression model. The results will be presented, including the sample size, the Odds Ratio (exp(ß)) of the treatment effect, its 99% confidence interval (CI), standard error, and p-value. The analysis will be performed among the ITT and PP population.

7.8 Postoperative morbidity – Comprehensive Complication Index

The Comprehensive Complication Index for postoperative morbidity is regarded a continuous variable (although it is limited between 0-100) and defined as described in section 1.3.2. The Comprehensive Complication Index for postoperative morbidity is the dependent variable. The model will contain the covariables described in section 3.5. The relationship will be assessed using a random effects linear regression model. The model results will be presented, including the sample size, the increment (ß) of the treatment effect, its 99% confidence interval (CI), standard error, and p-value. Prior to the model being fitted, normality assumption will be checked and handled as described in section 3.8.1. The analysis will be performed among the ITT and PP population.

7.9 Postoperative morbidity – Composite endpoint

The composite endpoint for postoperative morbidity is measured dichotomously (yes, no) and defined as described in section 1.3.2. The composite endpoint for postoperative morbidity is the dependent variable. The model will contain the covariables described in section 3.5. The relationship will be assessed using a random effects logistic regression model. The results will be presented, including the sample size, the Odds Ratio (exp(ß)) of the treatment effect, its 99% confidence interval (CI), standard error, and p-value. The analysis will be performed among the ITT and PP population.

7.10 90-Day mortality

90-Day mortality is measured dichotomously (yes, no) and defined as described in section 1.3.2. 90-Day mortality is the dependent variable. The model will contain the covariables described in section 3.5. The relationship will be assessed using a random effects logistic regression model. The results will be presented, including the sample size, the Odds Ratio (exp(ß)) of the treatment effect, its 99% confidence interval (CI), standard error, and p-value. The analysis will be performed among the ITT and PP population.

7.11 Readmission

Readmission is measured dichotomously (yes, no) and categorically (no readmission, surgery-related readmission, non surgery-related readmission) and defined as described in section 1.3.2. Readmission is the dependent variable. The model will contain the covariables described in section 3.5. The relationship will be assessed using a random effects logistic regression model for the dichotomously outcome and using a random effects multinomial logistic regression model for the categorically outcome. The results will be presented, including the sample size, the Odds Ratio (exp(ß)) of the treatment effect, its 99% confidence interval (CI), standard error, and p-value. The analysis will be performed among the ITT and PP population.

7.12 Resection margin

Resection margin is measured categorically (R0, R1, R2, and Rx) and defined as described in section 1.3.2. Resection margin is the dependent variable. The model will contain the covariables described in section 3.5. The relationship will be assessed using a random effects multinomial logistic regression model. The results will be presented, including the sample size, the Odds Ratio (exp(ß)) of the treatment effect, its 99% confidence interval (CI), standard error, and p-value. The analysis will be performed among the ITT and PP population.

7.13 Time to adjuvant chemotherapy initiation

Time to adjuvant chemotherapy initiation is measured in days (continuous) and defined as described in section 1.3.2. Time to adjuvant chemotherapy initiation is the dependent variable. The model will contain the covariables described in section 3.5. The relationship will be assessed using a random effects cox proportional hazards model. The results will be presented, including the sample size, the Hazard Ratio (HR) of the treatment effect, its 99% confidence interval (CI), standard error, and p-value. Prior to the model being fitted, the proportional hazard assumption of treatment arm on time to death will be checked and handled as described in section 3.8.2. The analysis will be performed among the ITT and PP population.

7.14 Quality of life – EORTC QLQ-C30 – Global health score

The EORTC QLQ-C30 global health score is regarded a continuous variable (although it is limited between 0-100) and defined as described in section 1.3.2. The EORTC QLQ-C30 global health score is the dependent variable. The model will contain the covariables described in section 3.5. The relationship will be assessed using a random effects linear regression model. The model results will be presented, including the sample size, the increment (ß) of the treatment effect, its 99% confidence interval (CI), standard error, and p-value. Prior to the model being fitted, normality assumption will be checked and handled as described in section 3.8.1. The analysis will be performed among the ITT and PP population.

7.15 Quality of life – EORTC QLQ-C30 – Functional score

The EORTC QLQ-C30 functional score is regarded a continuous variable (although it is limited between 0-100) and defined as described in section 1.3.2. The EORTC QLQ-C30 functional score is the dependent variable. The model will contain the covariables described in section 3.5. The relationship will be assessed using a random effects linear regression model. The model results will be presented, including the sample size, the increment (ß) of the treatment effect, its 99% confidence interval (CI), standard error, and p-value. Prior to the model being fitted, normality assumption will be checked and handled as described in section 3.8.1. The analysis will be performed among the ITT and PP population.

7.16 Quality of life – EORTC QLQ-LMC21 – Symptoms score

The EORTC QLQ-LMC21 symptoms score is regarded a continuous variable (although it is limited between 0-100) and defined as described in section 1.3.2. The EORTC QLQ-LMC21 symptoms score is the dependent variable. The model will contain the covariables described in section 3.5. The relationship will be assessed using a random effects linear regression model. The model results will be presented, including the sample size, the increment (ß) of the treatment effect, its 99% confidence interval (CI), standard error, and p-value. Prior to the model being fitted, normality assumption will be checked and handled as described in section 3.8.1. The analysis will be performed among the ITT and PP population.

7.17 Body image score

The Body image score is regarded a continuous variable (although it is limited between 5-20) and defined as described in section 1.3.2 The Body image score is the dependent variable. The model will contain the covariables described in section 3.5. The relationship will be assessed using a random effects linear regression model. The model results will be presented, including the sample size, the increment (ß) of the treatment effect, its 99% confidence interval (CI), standard error, and p-value. Prior to the model being fitted, normality assumption will be checked and handled as described in section 3.8.1. The analysis will be performed among the ITT and PP population.

7.18 Cosmesis score

The Cosmesis score is regarded a continuous variable (although it is limited between 3-24) and defined as described in section 1.3.2 The Cosmesis score is the dependent variable. The model will contain the covariables described in section 3.5. The relationship will be assessed using a random effects linear regression model. The model results will be presented, including the sample size, the increment (ß) of the treatment effect, its 99% confidence interval (CI), standard error, and p-value. Prior to the model being fitted, normality assumption will be checked and handled as described in section 3.8.1. The analysis will be performed among the ITT and PP population.

7.19 Delay in discharge after functional recovery

Delay in discharge after functional recovery is measured in days (continuous) and defined as described in section 1.3.2. Delay in discharge after functional recovery is the dependent variable. The model will contain the covariables described in section 3.5. The relationship will be assessed using a random effects linear regression model. The model results will be presented, including the sample size, the increment (ß) of the treatment effect, its 99% confidence interval (CI), standard error, and p-value. Prior to the model being fitted, normality assumption will be checked and handled as described in section 3.8.1. The analysis will be performed among the ITT and PP population.

7.20 12-Month incidence of incisional hernia

12-Month incidence of incisional hernia is measured dichotomously (yes, no) and defined as described in section 1.3.2. 12-Month incidence of incisional hernia is the dependent variable. The model will contain the covariables described in section 3.5. The relationship will be assessed using a random effects logistic regression model. The results will be presented, including the sample size, the Odds Ratio (exp(ß)) of the treatment effect, its 99% confidence interval (CI), standard error, and p-value. The analysis will be performed among the ITT and PP population.

7.21 Direct and indirect costs

Direct and indirect costs are measured in euros (€) and defined as described in section 1.3.2. Summary descriptives per treatment arm will be presented.

7.22 Overall 5-year survival

The 5 year survival probability per treatment arm will be computed using a Kaplan-Meier method. In addition the overall 5-year survival rates between treatment arms will be compared using a random effects Cox proportional hazards model. Time to death was calculated from the date of surgery to the date of death from any cause, and censoring the follow-up time at 5-years for living patients. . Five-year survival is the dependent variable. The model will contain the covariables described in section 3.5. The results will be presented, including the sample size, the Hazard Ratio (HR) of the treatment effect, its 99% confidence interval (CI), standard error, and p-value. Prior to the model being fitted, the proportional hazard assumption of treatment arm on 5-year survival will be checked and handled as described in section 3.8.2. The analysis will be performed among the ITT and PP population.

Primary and secondary outcomes					
**Primary outcome**	**Open surgery (n=)**	**Laparoscopic surgery (n=)**	**ß (96% CI)**	**SE**	**p-value**
Time to functional recovery – days					
Missing					
**Secondary outcomes**	**Open surgery (n=)**	**Laparoscopic surgery (n=)**	**ß (99% CI) / OR / HR**	**SE**	**p-value**
Hospital length of stay – days					
Missing					
Intraoperative blood loss – millilitres					
Missing					
Operation time – minutes					
Missing					
Conversions – n (%)					
None					
Urgent reason					
Non-urgent reason					
Unknown reason					
Missing					
Unfavourable intraoperative incidents – n (%)					
None					
Satava grade 1					
Satave grade 2					
Satava grade 3					
Missing					
Postoperative morbidity					
Accordion classification – n (%)					
Grade 1					
Grade 2					
Grade 3					
Grade 4					
Grade 5					
Grade 6					
Missing					
Clavien-Dindo classification – yes (%)					

Grade 1<br>Grade 2<br>Grade 3A<br>Grade 3B<br>Grade 4<br><br>Grade 5					
Missing					
Comprehensive complication index - points					
Missing					
Composite endpoint – yes, no					
Missing					
90-Day mortality – yes, no					
Missing					
Readmission – yes (%)<br>No readmission<br>Surgery-related readmission<br>Non surgery-related readmission					
Missing					
Resection margin<br>R0<br>R1<br>R2<br>Rx					
Missing					
Time to adjuvant chemotherapy initiation – days					
Missing					
Quality of life					
EORTC QLQ-C30 – summary score<br>EORTC QLQ-C30 – global health score<br>EORTC QLQ-C30 – functional score<br>EORTC QLQ-C30 – symptoms score					
Missing					
Body image score					

Missing					
Cosmesis score					
Missing					
Delay in discharge after functional recovery – days					
Missing					
12-month incidence of incisional hernia – yes, no					
Missing					
Treatment costs					
Direct treatment costs					
Indirect treatment costs					
Other costs					
Missing					
Overall 5-year survival (%)					
Related to liver surgery					
Due to progressive/metastatic disease					
Due to any other reason					
Unknown reason					
Missing					

SECTION 8 SUBGROUP ANALYSES

Additional subgroup analyses

Additional subgroup analyses will be performed to assess whether outcome differences between the open and the laparoscopic treatment arms differ across subgroups based on the following baseline variables: BMI (18-<25 kg/m² / 25-<30kg/m² / ≥ 30 kg/m²), malignant liver tumours (colorectal cancer liver metastases / hepatocellular carcinoma / cholangiocarcinoma / other malignant liver tumours), WHO performance status (grade 0 or 1 / grade 2 and above), ASA physical status (grade 1 / grade 2 / grade 3) the preceding of preoperative portal venous embolisation (yes / no), the preceding of preoperative chemotherapy (yes / no), the preceding of abdominal surgery (yes / no), the occurrence of conversions (yes / no), the occurrence of unfavourable intraoperative incidents (no / Satava grade 1 / Satava grade 2 or 3), the amount of interaoperative blood loss (less than 500 mL / between 500 and 1000 mL / between 1000 and 2000ml/ more than 2000 mL), the use of intraoperative inotropic medication (yes / no), the occurrence of postoperative complications (no complications / Clavien-Dindo grade 1 or 2 / Clavien-Dindo grade 3 or higher), the occurrence of morbidity according to the composite endpoint after liver surgery complications (no morbidity / morbidity), Comprehensive Complication Index (score of 0-20 / 21-26 /27-40/ 41-100), Quality of liver parenchyma (Normal liver / steatosis or fibrosis / severe hepatitis or cirrhosis).

In view of the added risk of both false positive results due to multiple testing and false negatives due to reduced sample size, the subgroups are restricted to the most important subgroups with highest clinical impact and only assessed for time to functional recovery (FR) and length of hospital stay (LOS). These are pre-specified subgroup analyses, of which the effect estimates including p-value, and confidence intervals will be presented for each subgroup separately. Because this trial was not powered to detect potential interaction between treatment and subgrouping factor, subgroup analyses will be done even if no significant interaction is found, but its results need to be interpreted as tentative pending replication. In view of the large number of tests performed, the report will state this number explicitly to caution against overinterpretations of what may be type I errors due to multiple testing. In view of the modest sample size per subgroup, confidence intervals will be provided on top of p-values to caution against overinterpretations of what may be type II errors due to small sample size.

For the subgroup analyses a minimum number of n = 30 patients (central limit theorem) would be required in each subgroup for the analyses to be performed, and even that will be too small for sufficient power unless the effect is very large. For the subgroup analyses, only the minimisation factors described in Section 2.3 will be used as covariates. Similar to the main analysis, time to functional recovery will be tested here with p=0.04 and LOS with p=0.01 as specified in section 3.3.1.

For all these covariate and abovementioned outcomes, the following analyses will be performed:
Firstly, interaction of treatment with covariate will be tested.
Secondly, the sample is divided into subgroups of at least 30 patients per subgroup, per arm, based on the covariate, and two treatment arms are then compared within each subgroup.

In view of multiple testing, significant results from these subgroup analysis must be treated as exploratory, especially if no significant interaction is found. And, in view of the reduced sample size in the subgroups, these subgroup analysis will have low power and precision.

8.1 Normal versus overweight versus obesity body mass index

- Normal weight: 18 ≤ kg/m² < 25.
- Overweight: 25 ≤ kg/m² < 30.
- Obese: ≥ 30 kg/m².

8.2 Malignant liver tumours

- Colorectal liver cancer metastases.

- Hepatocellular carcinoma.
- Cholangiocarcinoma.
- Other malignant liver tumours.

8.3 Preoperative portal venous embolisation versus no preoperative venous embolization (ORANGE II PLUS arms only)

- No preoperative portal venous embolisation.
- Preoperative portal venous embolisation.

8.4 Preoperative chemotherapy versus no preoperative chemotherapy

- No preoperative chemotherapy.
- Preoperative chemotherapy.

8.5 Previous abdominal surgery versus no previous abdominal surgery

- No previous abdominal surgery.
- Previous abdominal surgery.

8.6 World Health Organisation performance status

- WHO status grade 0 or 1.
- WHO status grade 2 and above.

8.7 American society of Anaesthesiologists physical status classification system

- ASA grade 1.
- ASA grade 2.
- ASA grade 3.

8.8 Conversions

- No conversions.
- Conversion to open or hand-assisted liver resection.

8.9 Unfavourable intraoperative incidents

- No unfavourable intraoperative incidents.
- Satava grade 1 unfavourable intraoperative incidents.
- Satava grade 2 or 3 unfavourable intraoperative incidents.

8.10 Intraoperative blood loss (ORANGE II PLUS)

- $<$ 500 mL.
- 500 mL $\leq$ blood loss $<$ 1000 mL.
- 1000 mL $\leq$ blood loss $<$ 2000 mL.
- $\geq$ 2000 mL.

8.11 Intraoperative blood loss (ORANGE SEGMENTS)

- $<$ 250 mL.
- 250 mL $\leq$ blood loss $<$ 500 mL.
- 500 mL $\leq$ blood loss $<$ 1000 mL.
- $\geq$ 1000 mL.

8.12 Additional ablation or resection of metastasis

- Additional ablation or resection of metastasis
- No additional ablation or resection of metastasis

8.13 Intraoperative inotropic medication

- No intraoperative inotropic medication.
- Intraoperative inotropic medication.

8.14 Postoperative complications

- No postoperative complications.
- Minor postoperative complications (Clavien-Dindo grade 1 or 2)
- Severe postoperative complications (Clavien-Dindo grade 3 or higher)

8.15 Composite endpoint of morbidity after liver surgery complications

- No morbidity.
- Morbidity.

8.16 Comprehensive Complication Index

- $0 \leq$ CCI score ≤ 20.
- $20 <$ CCI score ≤ 26.
- $26 <$ CCI score ≤ 40.
- $40 <$ CCI score ≤ 100.

8.17 Quality of liver parenchyma

- Normal liver
- Steatosis / fibrosis
- Severe hepatitis / cirrhosis

META-ANALYSES

Individual patient outcomes from the ORANGE II PLUS and ORANGE SEGMENTS trials will be combined for a pooled analysis using appropriate methods, which will be determined after the trials have been published.[44]

Individual patient outcomes from the ORANGE II PLUS and OSLO-COMET trials will be combined for a pooled analysis using appropriate methods, which will be determined after both trials have been published.[45]

SECTION 9 SAFETY OUTCOMES

9.1 Safety outcomes at interim

The trial is stopped if the mortality after hepatectomy (either hemihepatectomy or parenchymal preserving poster-superior liver segment resection) exceeds 5% in patients with normal liver function or exceeds 10% in cirrhotic patients.

9.2 Safety outcomes at end of the trial

Safety outcomes are analysed in-depth in accordance with sections 7.4, 7.5, 7.6, 7.7, 7.8, 7.9, 7.10, 7.11 and 7.24.

Safety outcomes					
	Open surgery (n=)	Laparoscopic surgery (n=)	OR / ß (99% CI)	SE	p-value
Serious adverse event – no./total no. (%)					
Prolonged admission (>10 days)					
Readmission (<30 days)					
Life-threatening (ICU admission)					
Permanent or serious invalidity					
Death (<90 days)					
Missing					
Adverse event – no./total no. (%)					
Grade 2					
Grade 3A					
Grade 3B					
Grade 4A					
Grade 4B					
Grade 5					
Missing					
Comprehensive complication index – points					
Missing					
Type of adverse event (≥ grade 2)– no. (%)					
Pleural effusion					
Ascites					
Wound infection					
Intra-abdominal abscess					
Bile leakage					
Postoperative liver failure					
Pneumonia					
Intra-abdominal haemorrhage					
Intra-abdominal haematoma					
Acute renal failure					
Blood transfusion					
Other adverse event					
Missing					
Postoperative morbidity – composite endpoint					

Missing					

SECTION 10 REFERENCES

1 Stoot, J. H. *et al.* The effect of a multimodal fast-track programme on outcomes in laparoscopic liver surgery: a multicentre pilot study. *HPB (Oxford)* **11**, 140-144, doi:10.1111/j.1477-2574.2009.00025.x (2009).

2 Maessen, J. M., Dejong, C. H., Kessels, A. G., von Meyenfeldt, M. F. & Enhanced Recovery After Surgery, G. Length of stay: an inappropriate readout of the success of enhanced recovery programs. *World J Surg* **32**, 971-975, doi:10.1007/s00268-007-9404-9 (2008).

3 Suurmeijer, T. P. *et al.* The Groningen Activity Restriction Scale for measuring disability: its utility in international comparisons. *American Journal of Public Health* **84**, 1270-1273 (1994).

4 Satava, R. M. Identification and reduction of surgical error using simulation. *Minim Invasive Ther Allied Technol* **14**, 257-261, doi:10.1080/13645700500274112 (2005).

5 Kazaryan, A. M., Rosok, B. I., Marangos, I. P., Rosseland, A. R. & Edwin, B. Comparative evaluation of laparoscopic liver resection for posterosuperior and anterolateral segments. *Surg Endosc* **25**, 3881-3889, doi:10.1007/s00464-011-1815-x (2011).

6 Strasberg, S. M., Linehan, D. C. & Hawkins, W. G. The accordion severity grading system of surgical complications. *Ann Surg* **250**, 177-186, doi:10.1097/SLA.0b013e3181afde41 (2009).

7 Porembka, M. R., Hall, B. L., Hirbe, M. & Strasberg, S. M. Quantitative weighting of postoperative complications based on the accordion severity grading system: demonstration of potential impact using the american college of surgeons national surgical quality improvement program. *J Am Coll Surg* **210**, 286-298, doi:10.1016/j.jamcollsurg.2009.12.004 (2010).

8 Dindo, D., Demartines, N. & Clavien, P. A. Classification of surgical complications: a new proposal with evaluation in a cohort of 6336 patients and results of a survey. *Ann Surg* **240**, 205-213, doi:10.1097/01.sla.0000133083.54934.ae (2004).

9 Slankamenac, K. *et al.* The comprehensive complication index: a novel and more sensitive endpoint for assessing outcome and reducing sample size in randomized controlled trials. *Ann Surg* **260**, 757-762; discussion 762-753, doi:10.1097/SLA.0000000000000948 (2014).

10 van den Broek, M. A. *et al.* Feasibility of randomized controlled trials in liver surgery using surgery-related mortality or morbidity as endpoint. *Br J Surg* **96**, 1005-1014, doi:10.1002/bjs.6663 (2009).

11 Lamers, L. M., Stalmeier, P. F., McDonnell, J., Krabbe, P. F. & van Busschbach, J. J. [Measuring the quality of life in economic evaluations: the Dutch EQ-5D tariff]. *Ned Tijdschr Geneeskd* **149**, 1574-1578 (2005).

12 Lamers, L. M., McDonnell, J., Stalmeier, P. F., Krabbe, P. F. & Busschbach, J. J. The Dutch tariff: results and arguments for an effective design for national EQ-5D valuation studies. *Health Econ* **15**, 1121-1132, doi:10.1002/hec.1124 (2006).

13 Langenhoff, B. S., Krabbe, P. F., Peerenboom, L., Wobbes, T. & Ruers, T. J. Quality of life after surgical treatment of colorectal liver metastases. *Br J Surg* **93**, 1007-1014, doi:10.1002/bjs.5387 (2006).

14 Blazeby, J. M. *et al.* Validation of the European Organization for Research and Treatment of Cancer QLQ-LMC21 questionnaire for assessment of patient-reported outcomes during treatment of colorectal liver metastases. *Br J Surg* **96**, 291-298, doi:10.1002/bjs.6471 (2009).

15 Fayers, P., Bottomley, A., Group, E. Q. o. L. & Quality of Life, U. Quality of life research within the EORTC-the EORTC QLQ-C30. European Organisation for Research and Treatment of Cancer. *Eur J Cancer* **38 Suppl 4**, S125-133 (2002).

16 Cleemput, I. A social preference valuations set for EQ-5D health states in Flanders, Belgium. *Eur J Health Econ* **11**, 205-213, doi:10.1007/s10198-009-0167-0 (2010).

17 Wittrup-Jensen, K. U., Lauridsen, J., Gudex, C. & Pedersen, K. M. Generation of a Danish TTO value set for EQ-5D health states. *Scand J Public Health* **37**, 459-466, doi:10.1177/1403494809105287 (2009).

18 Greiner, W., Claes, C., Busschbach, J. J. & von der Schulenburg, J. M. Validating the EQ-5D with time trade off for the German population. *Eur J Health Econ* **6**, 124-130 (2005).

19 Scalone, L. *et al.* Italian population-based values of EQ-5D health states. *Value Health* **16**, 814-822, doi:10.1016/j.jval.2013.04.008 (2013).

20 Burstrom, K. *et al.* Swedish experience-based value sets for EQ-5D health states. *Qual Life Res* **23**, 431-442, doi:10.1007/s11136-013-0496-4 (2014).
21 Dolan, P. Modeling valuations for EuroQol health states. *Med Care* **35**, 1095-1108 (1997).
22 Janssen, M. F. *et al.* Population norms for the EQ-5D-3L: a cross-country analysis of population surveys for 20 countries. *Eur J Health Econ* **20**, 205-216, doi:10.1007/s10198-018-0955-5 (2019).
23 Wintner, L. M. *et al.* The use of EORTC measures in daily clinical practice-A synopsis of a newly developed manual. *Eur J Cancer* **68**, 73-81, doi:10.1016/j.ejca.2016.08.024 (2016).
24 W Scott, N., M Fayers, P. & Aaronson, N. EORTC QLQ-C30 Reference Values. (2008).
25 Hinz, A., Singer, S. & Brahler, E. European reference values for the quality of life questionnaire EORTC QLQ-C30: Results of a German investigation and a summarizing analysis of six European general population normative studies. *Acta Oncol* **53**, 958-965, doi:10.3109/0284186X.2013.879998 (2014).
26 Martinelli, F. *et al.* Examining the relationships among health-related quality-of-life indicators in cancer patients participating in clinical trials: a pooled study of baseline EORTC QLQ-C30 data. *Expert Rev Pharmacoecon Outcomes Res* **11**, 587-599, doi:10.1586/erp.11.51 (2011).
27 Rees, J. R. *et al.* The Prognostic Value of Patient-Reported Outcome Data in Patients With Colorectal Hepatic Metastases Who Underwent Surgery. *Clin Colorectal Cancer* **15**, 74-81 e71, doi:10.1016/j.clcc.2015.07.003 (2016).
28 Heise, D. *et al.* Long-term outcome and quality of life after initial and repeat resection of colorectal liver metastasis: A retrospective analysis. *Int J Surg* **48**, 281-285, doi:10.1016/j.ijsu.2017.11.032 (2017).
29 Paradowska, D. *et al.* A prospective study to validate the Polish language version of the European Organisation for Research and Treatment of Cancer (EORTC) Colorectal Liver Metastases (QLQ-LMC21) module. *Eur J Oncol Nurs* **29**, 148-154, doi:10.1016/j.ejon.2017.05.006 (2017).
30 Rees, J. R. *et al.* Patient-reported outcomes in long-term survivors of metastatic colorectal cancer needing liver resection. *Br J Surg* **101**, 1468-1474, doi:10.1002/bjs.9620 (2014).
31 Banz, V. M., Inderbitzin, D., Fankhauser, R., Studer, P. & Candinas, D. Long-term quality of life after hepatic resection: health is not simply the absence of disease. *World J Surg* **33**, 1473-1480, doi:10.1007/s00268-009-0032-4 (2009).
32 Studer, P., Horn, T., Haynes, A., Candinas, D. & Banz, V. M. Quality of life after hepatic resection. *Br J Surg* **105**, 237-243, doi:10.1002/bjs.10735 (2018).
33 Maessen, J. *et al.* A protocol is not enough to implement an enhanced recovery programme for colorectal resection. *Br J Surg* **94**, 224-231, doi:10.1002/bjs.5468 (2007).
34 Dagher, I. *et al.* Laparoscopic major hepatectomy: an evolution in standard of care. *Ann Surg* **250**, 856-860, doi:10.1097/SLA.0b013e3181bcaf46 (2009).
35 Dagher, I. *et al.* Laparoscopic versus open right hepatectomy: a comparative study. *Am J Surg* **198**, 173-177, doi:10.1016/j.amjsurg.2008.09.015 (2009).
36 Jarnagin, W. R. *et al.* Improvement in perioperative outcome after hepatic resection: analysis of 1,803 consecutive cases over the past decade. *Ann Surg* **236**, 397-406; discussion 406-397, doi:10.1097/01.SLA.0000029003.66466.B3 (2002).
37 Scuderi, V. *et al.* Outcome after laparoscopic and open resections of posterosuperior segments of the liver. *Br J Surg*, doi:10.1002/bjs.10489 (2017).
38 DeMets, D. L. & Lan, G. The alpha spending function approach to interim data analyses. *Cancer Treat Res* **75**, 1-27 (1995).
39 Julious, S. A. Sample sizes for clinical trials with normal data. *Stat Med* **23**, 1921-1986, doi:10.1002/sim.1783 (2004).
40 Jennison, C. & Turnbull, B. W. Meta-analyses and adaptive group sequential designs in the clinical development process. *J Biopharm Stat* **15**, 537-558, doi:10.1081/BIP-200062273 (2005).
41 Proschan, M. A. Sample size re-estimation in clinical trials. *Biom J* **51**, 348-357, doi:10.1002/bimj.200800266 (2009).
42 Schulz, K. F., Altman, D. G., Moher, D. & Group, C. CONSORT 2010 statement: updated guidelines for reporting parallel group randomised trials. *BMJ* **340**, c332, doi:10.1136/bmj.c332 (2010).

43 Kayembe, M. T., Jolani, S., Tan, F. E. S. & van Breukelen, G. J. P. Imputation of missing covariate in randomized controlled trials with a continuous outcome: Scoping review and new results. *Pharm Stat* **19**, 840-860, doi:10.1002/pst.2041 (2020).
44 Debray, T. P. *et al.* Get real in individual participant data (IPD) meta-analysis: a review of the methodology. *Res Synth Methods* **6**, 293-309, doi:10.1002/jrsm.1160 (2015).
45 Fretland, A. A. *et al.* Laparoscopic Versus Open Resection for Colorectal Liver Metastases: The OSLO-COMET Randomized Controlled Trial. *Ann Surg* **267**, 199-207, doi:10.1097/SLA.0000000000002353 (2018).
