# Supplementary material for: Health-related quality of life in patients undergoing laparoscopic versus open hemihepatectomy: a secondary analysis of the ORANGE II PLUS randomised controlled, phase 3, superiority trial
Source: Lancet Reg Health Eur. 2025 May 19;54:101311. doi: 10.1016/j.lanepe.2025.101311 (PMC12148447; doi:10.1016/j.lanepe.2025.101311)
Supplement: ORANGE-II-PLUS final study protocol [file mmc2.pdf]

RESEARCH PROTOCOL

The ORANGE II PLUS –Trial

PROTOCOL TITLE: The ORANGE II PLUS – Trial: An international multicentre randomized controlled trial of open versus laparoscopic liver surgery (hemihepatectomy or postero-superior liver segment resection).

**Protocol ID**	**NL36215.068.11**
**Short title**	**ORANGE II PLUS - Trial**
**Version**	**12**
**Date**	**March 30, 2017**
**Coordinating investigator/project leader**	***Dr. R.M. van Dam***<br>**Maastricht University Medical Center+**<br>**r.vandam@mumc.nl**<br>**+31433877489**
**Principal investigators**	
Netherlands	***Dr. R.M. van Dam***<br>**Maastricht University Medical Center+**<br>**r.vandam@mumc.nl**
United Kingdom	***Prof. J.N. Primrose***<br>**University Hospital Southampton**<br>**j.n.primrose@soton.ac.uk**
Belgium	***Prof. R.I. Troisi***<br>**University Hospital Ghent**<br>**roberto.troisi@ugent.be**
Italy	***Prof. L.A. Aldrighetti***<br>**San Raffaele Hospital**<br>**aldrighetti.luca@hsr.it**
Norway	***Prof. B.E. Edwin***<br>**University Hospital Oslo**<br>**bjoedw@ous-hf.no**
Germany	***Prof. U.P. Neumann***<br>**University Hospital Aachen**<br>**uneumann@ukaachen.de**
**Sponsor**	**Maastricht University Medical Center+**
**Independent physician**	***Dr. M. Poeze***<br>**Maastricht University Medical Center+**<br>**m.poeze@mumc.nl**

PROTOCOL SIGNATURE SHEET

Name	Signature	Date
**Head of department:**<br>**Maastricht University Medical Center+**<br>***Prof. dr. U.P. Neumann***		**2017, March 13**
**Principal investigator for the hemihepatectomy study part:**<br>***Dr. R.M. van Dam***		**2017, March 13**
**Principal investigator for the postero-superior liver segment study part:**<br>***Dr. M. Abu Hilal***		**2017, March 13**
**Study coordinator:**<br>***Drs. R.S. Fichtinger***		**2017, March 13**

TABLE OF CONTENTS

RESEARCH PROTOCOL 1
PROTOCOL SIGNATURE SHEET 3
TABLE OF CONTENTS 4
LIST OF ABBREVIATIONS AND RELEVANT DEFINITIONS 8
SUMMARY 10
1. INTRODUCTION AND RATIONALE 12
2. OBJECTIVES 15
3. STUDY DESIGN 16
4. STUDY POPULATION 17
4.1 Population (base) 17
4.2 Inclusion criteria 17
4.3 Exclusion criteria 17
4.4 Sample size calculation 18
4.4.1 Hemihepatectomy. 18
4.4.2 Hemihepatectomy sample size at interim analysis 19
4.4.3 One or two postero-superior liver segments 4A, 7, 8. 20
5. TREATMENT OF SUBJECTS 21
5.1 Investigational product/treatment 21
5.2 Use of co-intervention 21
5.3 Escape medication 21
6. INVESTIGATIONAL MEDICINAL PRODUCT 22
6.1 Name and description of Investigational Medicinal Product 22
6.2 Summary of findings from non-clinical studies 22
6.3 Summary of findings from clinical studies 22
6.4 Summary of known and potential risks and benefits 22
6.5 Description and justification of route of administration and dosage 22
6.6 Dosages, dosage modifications and method of administration 22
6.7 Preparation and labelling of Investigational Medicinal Product 22
6.8 Drug accountability 22
7. NON-INVESTIGATIONAL PRODUCT 23
7.1 Name and description of Non-Investigational Medicinal Product(s) 23
7.2 Summary of findings from non-clinical studies 23
7.3 Summary of findings from clinical studies 23
7.4 Summary of known and potential risks and benefits 23
7.5 Description and justification of route of administration and dosage 23

7.6 Dosages, dosage modifications and method of administration 23
7.7 Preparation and labelling of Non-Investigational Medicinal Product....23
7.8 Drug accountability....23
8. METHODS 24
8.1 Study parameters/endpoints....24
8.1.1 Main study parameter/endpoint 24
8.1.2 Secondary study parameters/endpoints 24
8.1.3 Other study parameters....24
8.2 Randomization, blinding and treatment allocation....25
8.2.1 Randomization 25
8.2.2 Blinding 25
8.3 Study procedures 26
8.3.1 Functional recovery 26
8.3.2 Surgery 27
8.3.3 Laboratory....27
8.3.4 Morbidity 27
8.3.5 Liver resection specific composite endpoint....28
8.3.6 Pathology 28
8.3.7 Quality of life 28
8.3.8 Body image and cosmesis....29
8.3.9 Long term incidence of incisional hernias 29
8.3.10 Economic evaluation 29
8.3.11 Oncological endpoints 30
8.4 Withdrawal of individual subjects....30
8.5 Replacement of individual subjects after withdrawal....30
8.6 Follow-up of subjects withdrawn from treatment....30
8.7 Premature termination of the study....31
8.8 Section 10 WMO event 31
8.9 AEs, SAEs and SUSARs....31
8.9.1 Adverse events (AE's)....31
8.9.2 Serious adverse events (SAEs)....31
8.9.3 Suspected unexpected serious adverse reactions (SUSARs) 33
8.10 Annual safety report 33
8.11 Follow-up of adverse events....33
8.12 Data Safety Monitoring Board (DSMB) 33
9. STATISTICAL ANALYSIS 34
9.1 Descriptive statistics....34

9.2 Inferential analysis....34
9.3 Interim analysis....35
9.3.1 Stopping for significance, 2-day difference in functional recovery....35
9.3.2 Stopping for safety....35
9.3.3 Stopping for significance, 3-day difference in functional recovery (postero-superior liver segment resection only)....35
10. ETHICAL CONSIDERATIONS....36
10.1 Regulation statement....36
10.2 Recruitment and consent....36
10.3 Objection by minors or incapacitated subjects....36
10.4 Benefits and risks assessment, group relatedness....36
10.5 Compensation for injury....38
10.6 Incentives....38
11. ADMINISTRATIVE ASPECTS AND PUBLICATION....39
11.1 Handling and storage of data and documents....39
11.2 Monitoring and Quality Assurance....39
11.3 Amendments....39
11.4 Annual progress report....39
11.5 End of study report....40
11.6 Public disclosure and publication policy....40
12. STRUCTURED RISK ANALYSIS....41
12.1 Potential issues of concern....41
a. Level of knowledge about mechanism of action Not applicable. 41
b. Previous exposure of human beings with the test product(s) and/or products with a similar biological mechanism Not applicable....41
c. Can the primary or secondary mechanism be induced in animals and/or in ex- vivo human cell material?....41
d. Selectivity of the mechanism to target tissue in animals and/or human beings....41
e. Analysis of potential effect....41
f. Pharmacokinetic considerations....41
g. Study population....41
h. Interaction with other products....41
i. Predictability of effect....41
j. Can effects be managed?....41
13. SYNTHESIS....42
14. REFERENCES....44
15. APPENDICES....48

Appendix 1: ERAS® major abdominal surgery components 48
Appendix 2: Daily guidelines of care for patients undergoing hepatectomy in the ERAS® program 49
Appendix 3: ORANGE II PLUS – Trial flowchart 50
Appendix 4: SPIRIT flowchart 50
Appendix 5: ERAS® Mobility Score (EMS) 52
Appendix 6: Functional recovery checklist 53
Appendix 7: ORANGE II PLUS - Trial participating centres 54
Appendix 8: Satava classification adapted for liver surgery 55
Appendix 9: Accordion Severity Grading System of Surgical Complications 56
Appendix 10: EQ5D 57
    Appendix 10.1: EQ5D part 1 57
    Appendix 10.2: EQ5D part 2 58
Appendix 11: EORTC QLQ-C30 59
    Appendix 11.1: EORTC QLQ-C30 part 1 59
    Appendix 11.2: EORTC QLQ-C30 part 2 60
Appendix 12: EORTC QLQ-LM21 61
Appendix 13: Body Image Questionnaire 62
Appendix 14: Cost questionnaire 63
    Appendix 14.1: Cost questionnaire part 1 63
    Appendix 14.2: Cost questionnaire part 2 64
    Appendix 14.3: Cost questionnaire part 3 65

LIST OF ABBREVIATIONS AND RELEVANT DEFINITIONS

**ABR**	**ABR form (General Assessment and Registration form) is the application form that is required for submission to the accredited Ethics Committee (in Dutch: ABR = Algemene Beoordeling en Registratie)**
**AE**	**Adverse Event**
**AR**	**Adverse Reaction**
**BIQ**	**Body Image Questionnaire**
**CA**	**Competent Authority**
**CCMO**	**Central Committee on Research Involving Human Subjects**
**CDM**	**Clinical Data Management**
**CV**	**Curriculum Vitae**
**DSMB**	**Data Safety Monitoring Board**
**EDC**	**Electronic Data Capture**
**EMS**	**ERAS Mobility Scale**
**ERAS**	**Enhanced Recovery After Surgery**
**EU**	**European Union**
**EudraCT**	**European drug regulatory affairs Clinical Trials**
**GCP**	**Good Clinical Practice**
**IB**	**Investigator's Brochure**
**IC**	**Informed Consent**
**LOS**	**Length Of Stay in hospital**
**IMP**	**Investigational Medicinal Product**
**IMPD**	**Investigational Medicinal Product Dossier**
**METC**	**Medical Research Ethics Committee (MREC); in Dutch: Medisch Ethische Toetsing Commissie (METC)**
**SAE**	**Serious Adverse Event**
**PONV**	**PostOperative Nausea and Vomiting**
**SPC**	**Summary of product Characteristics (in Dutch: officiële productinfomatie IB1-tekst)**
**Sponsor**	**The Sponsor is the party that commissions the organization or performance of the research, for example a pharmaceutical company, academic hospital, scientific organization or investigator. A party that provides funding for a study but does not commission it is not regarded as the sponsor, but referred to as a subsidising party.**
**SUSAR**	**Suspected Unexpected Serious Adverse Reaction**

**WBP**	**Personal Data Protection Act (in Dutch: Wet Bescherming Persoonsgevens)**
**WMO**	**Medical Research Involving Human Subjects Act (in Dutch: Wet Medisch-wetenschappelijk Onderzoek met Mensen)**

SUMMARY

Rationale: Recent developments in major liver surgery include the introduction of laparoscopy and enhanced recovery programmes. However, randomized controlled trials comparing laparoscopy with the standard open surgical approach for hemihepatectomy or parenchymal sparing postero-superior liver segment resection have never been conducted.

Objective: The ORANGE II PLUS - Trial aims to provide evidence on the merits of laparoscopic surgery in two separate patient populations: either patients undergoing hemihepatectomy or patients having parenchymal sparing resection of the postero-superior liver segments.

Study design: The international multicentre ORANGE II PLUS - Trial is a prospective, double blinded, randomized controlled study of two separate and independent patient populations, each producing two arms (open versus laparoscopy) with a parallel registry. These populations involve patients undergoing left or right hemihepatectomy and patients having parenchymal preserving resection of one or two postero-superior liver segments (involving one or two of segments 4a, 7 or 8).

Study population: All patients - 18 years and older - with anatomic benign and/or malignant lesions requiring a hemihepatectomy with or without the need for one additional hepatic wedge resection or metastasectomy, or patients in need for a parenchymal sparing resection of postero-superior liver segments (involving one or two of segments 4a, 7, 8) that the multidisciplinary team feel is technically feasible and safe by either the laparoscopic or open approach.

Intervention:

a) Open or laparoscopic hemihepatecomy (with or without the need for one additional hepatic wedge resection or metastasectomy), stratified for centre and hemihepatectomy side (left or right).

b) Open or laparoscopic parenchymal sparing resection of postero-superior liver segments (one or two of segments 4a, 7, 8), stratified for centre and tumour size (smaller or larger than 3 cm).

All patients will participate in an enhanced recovery programme.

Main study parameters/endpoints: The primary endpoint is time to functional recovery. Secondary endpoints are length of stay in hospital (LOS), intraoperative blood loss, operation time, resection margin, time to adjuvant chemotherapy initiation, readmission percentage, (liver specific) morbidity, quality of life (QOL), body image, reasons for delay of discharge after functional recovery, long term incidence of incisional hernias, hospital and societal costs during one year, overall five-year survival.

Nature and extent of the burden and risks associated with participation, benefit and group relatedness: The burden and risks associated with the intervention (laparoscopic liver surgery) are comparable with the normal treatment (open liver surgery). Both laparoscopic hemihepatectomy and laparoscopic postero-superior liver resections have demonstrated to be feasible with good efficacy and safety in expert hands. Besides being blinded to the intervention, patients have to complete a patient diary during admission. Quality of life and body image questionnaires are obtained preoperatively and at 4 times during follow-up (10 days, 3 months, 6 months and 12 months after discharge), whereas cost-questionnaires will be requested 3 times (at 3 months, 6 months and 12 months after discharge). Ultimately, patients will receive an ultrasound to exclude incisional hernia at 12 months after discharge.

Clinicaltrial.gov identification number: NCT01441856

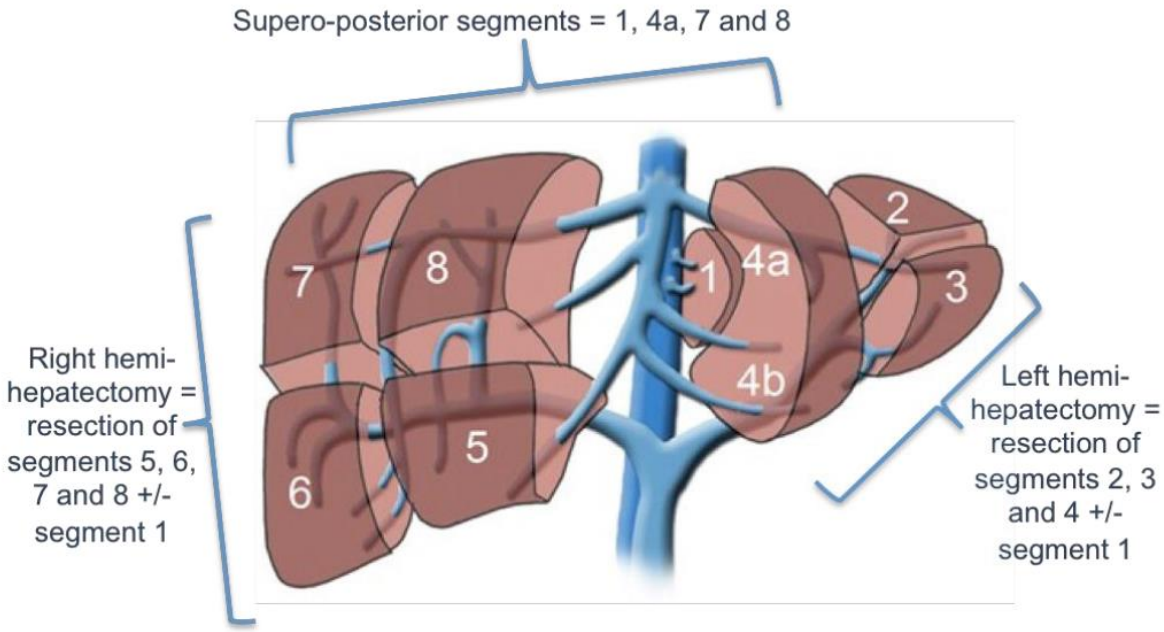

1. INTRODUCTION AND RATIONALE

Liver resection for colorectal metastasis is a potential curative therapy and has become the standard of care in appropriately staged patients, offering five-year survival rates ranging from 38 up to 61% in selected cases, with approximately 30% of patients surviving ten years or more, compared to five-year survival rates of less than 5% for patients not amenable to resection.[1-14] Liver surgery is also a widely accepted treatment for symptomatic benign lesions and those of uncertain nature or large size. Whilst the survival figures are a vast improvement on the past, there is still a need to refine the treatment of these patients, including surgical technique. Open hepatectomy is the current standard of care for the management of primary and secondary tumours. Both open hemihepatectomy and open postero-superior liver segment resection require a large incision to achieve adequate access and proper control during resection. This has a significant impact on patient's recovery and, in cases of small resections, this access may represent the major component of surgical trauma. Advances in surgical technique and expertise now permit these operations to be performed with minor incisions by using the laparoscopic approach. Although the feasibility of laparoscopic hepatectomy has been established, only select centres use this technique as their primary modality.[15-17]

Laparoscopic liver resection was first reported in 1991.[18-22] Over the past decades, the method has gained wide acceptance for various liver resection procedures.[23-25] Multiple retrospective case series, patient cohorts, systematic reviews and meta-analyses have compared open with laparoscopic liver surgery and indicate the laparoscopic approach to be safely applicable for the resection of both malignant and benign liver lesions.[26-31] Laparoscopic liver resection has been associated with shorter hospital length of stay, reduced intraoperative blood loss, less postoperative pain and earlier recovery.[29, 32-34] Despite this, concerns remain over operative times, the ability to control haemorrhage laparoscopically and long-term oncological outcomes.

Initially, the left lateral segments of the liver were chosen for anatomic laparoscopic resection, with good results. Many liver centres worldwide currently use laparoscopy for resection of the anterior liver segments. Whilst case control studies would now seem sufficient to allay such concerns in the context of minor liver resections and left lateral sectionectomies, the adoption and dissemination of laparoscopy by hepatobiliary oncologic surgeons for major hepatectomies and resections of postero-superior segments has been restricted.[23, 24, 26, 33, 35] Besides the relatively low volume of patients, major laparoscopic liver resections are technically demanding, have a significant learning curve,

are time consuming, are thought to hold an increased morbidity risk and lack in evidence. Nevertheless, a new impulse for the laparoscopic management of major liver lesions came after the first reports of laparoscopic hemihepatectomies, which demonstrated that in expert hands major anatomical laparoscopic liver resections are feasible with good efficacy and safety.[36-38]

When comparing surgical procedures, one of the easiest to measure and often used outcomes is the length of hospital stay; the time it takes for a patient to be discharged from the hospital after an operation. On the whole, a median hospital length of stay of 6.0 to 13.1 days and 3.5 to 10.0 days have been observed after open and laparoscopic hepatic resections in European centres respectively.[39] For major surgery in expert centres, median duration of hospital admission varied between 6 to 12.5 days for open surgery and 4 to 8.2 days for laparoscopic resections.[16, 38, 40-43] Concentrating on postero-superior liver segment resections, the median hospital stay is 6 days (3-44 days) for those undergoing open compared with 4 days (1-11 days) for those having laparoscopic resections.[44] Besides the immediate benefits to the patient, such as decreased intraoperative blood loss, diminished postoperative pain, earlier recovery and reduced hospital length of stay, laparoscopic liver surgery may also have the potential to improve outcomes in the longer term by reducing complications, enhancing quality of life, improving cosmesis, ensuring early commencement and completion of adjuvant therapies.[29, 32-34] However, level-1 evidence on all outcomes is still to be presented.[16, 17, 42, 43, 45, 46]

At the international consensus conference on laparoscopic liver surgery in Morioka in 2014, it was concluded that laparoscopic resections of postero-superior liver segments should be considered ‘major’ liver surgery; while parenchymal sparing resection of lesions in the postero-superior segments of the liver are often minor in terms of the resected liver volume, they are technically major due to the anatomical location of these segments. At that same conference, it was stated that major laparoscopic liver surgery (including hemihepatectomies and postero-superior liver segment resections amongst others) is to be regarded as an innovative procedure, still in its exploration or learning phase (IDEAL 2b) and ought to be introduced with great caution. Furthermore, as the quality of the available studies comparing open with laparoscopic major liver resections was generally designated to be low, there was a strong recommendation to organise higher quality studies.[46]

Besides improvements in major laparoscopic liver surgery, enthusiasm has also arisen for standardization of the perioperative care in the form of fast-track recovery protocols over the past decades, such as the Enhanced Recovery After Surgery (ERAS®) programme. This

fast-track recovery programme, derived from Kehlet's 1990's pioneer work in the multimodal surgical care field, involves optimization of several aspects of the perioperative management of patients undergoing major abdominal surgery (see *appendix 1*). In liver surgery, it was demonstrated that functional recovery and hospital length of stay after open and laparoscopic liver resection could be reduced when patients were managed within the multimodal ERAS® programme.[47, 48] Functional recovery can be regarded as the moment in time when it is medically justified to discharge a patient from hospital care. It is important to emphasize the discrepancy between hospital length of stay (LOS) and the time to functional recovery, which is influenced by tertiary (out-of-hospital) problems such as patient insecurity, problems in homecare support or logistic problems.[49] Scoring functional recovery instead of hospital length of stay may thus be an adequate, more reliable and better comparable outcome measure.

An evaluation of the benefits of the laparoscopic approach in patients undergoing major liver surgery, either hemihepatectomy or parenchymal preserving resection of postero-superior liver segments, is both timely and necessary and will inform clinical practice. Within the framework of optimized perioperative care, broader indications for hepatic surgery and further adoption of laparoscopic liver resections, there is a clear need for a randomized trial. Therefore, the multicentre and international ORANGE II PLUS – Trial has been designed to provide evidence on the merits of laparoscopic versus open hemihepatectomy and parenchymal preserving postero-superior liver segment resection within an enhanced recovery programme in terms of time to functional recovery, hospital length of stay, intraoperative blood loss, operation time, resection margin, time to adjuvant chemotherapy initiation, readmission percentage, (liver specific) morbidity, quality of life, body image, reasons for delay of discharge after functional recovery, long term incidence of incisional hernias, hospital and societal costs during one year and overall five-year survival.

2. OBJECTIVES

Hypotheses:

- For patients undergoing a laparoscopic left or right hemihepatectomy (with or without the need for one additional hepatic wedge resection or metastasectomy), time to functional recovery is reduced by 2 days in comparison with patients undergoing the open procedure.
- For patients undergoing parenchymal preserving laparoscopic resection of postero-superior liver segments, time to functional recovery is also reduced by 2 days compared with the open procedure.

Primary objective: to provide evidence on the merits of laparoscopic compared with open liver surgery, for standard indications and within an enhanced recovery programme in terms of time to functional recovery in:

- Patients undergoing either left or right hemihepatectomy (with or without the need for one additional hepatic wedge resection or metastasectomy).
- Patients undergoing parenchymal preserving resection of postero-superior liver segments.

Secondary objectives: to provide evidence on the benefits of laparoscopic compared with open surgery, for standard indications and within an enhanced recovery programme in terms of hospital length of stay, intraoperative blood loss, operation time, resection margin, time to adjuvant chemotherapy initiation, readmission percentage, (liver-specific) morbidity, quality of life, body image, reasons for delay of discharge after functional recovery, long term incidence of incisional hernias, hospital and societal costs during one year and overall five-year survival in:

- Patients undergoing either left or right hemihepatectomy (with or without the need for one additional hepatic wedge resection or metastasectomy).
- Patients undergoing parenchymal preserving resection of postero-superior liver segments.

3. STUDY DESIGN

The international and multicentre ORANGE II PLUS - Trial is a prospective, double blinded, randomized controlled study of two separate and independent patient populations, each producing two arms (open versus laparoscopy) with a parallel registry. These populations involve patients undergoing left or right hemihepatectomy and patients having parenchymal preserving resection of postero-superior liver segments (involving one or two of segments 4a, 7 or 8). All patients will be participating in an enhanced recovery programme. For daily guidelines of the pre-, intra- and postoperative care of patients undergoing major liver resection in the ERAS® programme, see *appendix 2.*

Baseline values of the primary and secondary outcomes are established before surgery. Next, data is gathered during surgery, while the patient is admitted and when the patient is discharged. Follow-up moments are planned at 10 days, 3, 6, 12 months and 5 years after discharge. Please see *appendix 3* for the trial flowchart and *appendix 4* for the SPIRIT flowchart.[50]

4. STUDY POPULATION

4.1 Population (base)

Patients are considered for hemihepatectomy (with or without the need for one additional metastasectomy or wedge resection) or parenchymal sparing postero-superior liver segment surgery only after careful evaluation by a multidisciplinary board that includes surgeons, radiologists, pathologists, hepathologists, oncologists and radiotherapists. At these multidisciplinary meetings, all specific in- and exclusion criteria for trial participation are assessed. Patients are approached for trial participation only if the multidisciplinary team feel the operation is technically feasible and safe by either the laparoscopic or open approach.

Trial candidates are informed of the study by their treating surgeon. Only after the patient has consented to be further informed about the study, he or she will be approached by the principal investigator, the authorized researcher, an informed surgical resident or a research nurse at the outpatient clinic of the surgical departments. After 1 week of reflection, a definitive personal written informed consent will be obtained by the principal investigator, the authorized researcher or the research nurse. All patients ineligible for randomization are asked to participate in the prospective registry. The number of ineligible patients and the reasons for randomization ineligibility are registered in screening logs.

4.2 Inclusion criteria

- Either: patients requiring left or right hemihepatectomy, with or without the need for one additional hepatic wedge resection or metastasectomy for accepted indications.
- Or: patients requiring a parenchymal sparing liver resection (including wedge resections and full segmentectomies) involving one or two of segments 4a, 7, 8 for accepted indications . A segment 6/7 resection would also be eligible.
- Able to understand the nature of the study and what will be required of them.
- Men and non-pregnant, non-lactating women aged 18 years and older.
- BMI between and including 18-35.
- Patients with ASA physical status I-II-III.

4.3 Exclusion criteria

- Inability to give (written) informed consent.
- Either: patients requiring another resection than left or right hemihepatectomy, with or without the need for one additional hepatic wedge resection or metastasectomy.

- Or: patients requiring other liver surgery than a parenchymal sparing resection involving one or two of segments 4a, 7, 8.
- Patients requiring parenchymal sparing liver resection involving segment 1. This is due to the high level of technical difficulty.
- Patients with hepatic lesion(s), that are located with insufficient margin from vascular or biliary structures to be operated laparoscopically.
- Patients with ASA physical status IV-V.
- Repeat hepatectomy.

4.4 Sample size calculation

4.4.1 Hemihepatectomy.

Since laparoscopic liver surgery focuses on accelerated recovery, time to functional recovery is used as the primary outcome parameter. In a prospectively collected database of three European, two U.S. and one Australian centre, a median hospital length of stay of 6.0 days (SD: 4.5) was observed after major laparoscopic hepatic resection. In European centres, the median LOS was significantly longer compared to the non-European hospitals (8.5 versus 5.9 days; p = 0.0001).[16] In a single centre report, of which data were also used in the previous reference, 22 laparoscopic and 50 open major liver resections were evaluated. This demonstrated a mean hospital length of stay of 8.2 days (SEM 1.1, SD = 5.1) after major laparoscopic hepatectomy.[51] A large analysis of Jarnagin et al. showed a mean duration of admission after open major liver resection of 11.1 days.[52] Based on these reports, mean LOS and SD after a laparoscopic hemihepatectomy in Europe can be estimated to be 8.5 and 5.0 days, respectively. Therefore, an average reduction in hospital length of stay of 2 days seems feasible.

We assume that in patients undergoing laparoscopic left or right hemihepatectomy, time to functional recovery is reduced in comparison to patients undergoing the open procedure. We aim at a reduction in time to functional recovery of 2 days. If we assume a standard deviation of 5.0 days for time to functional recovery, the implied effect size *d* will be 0.40 (2/5). If we also take into account a drop-out rate of 10% and a loss in degrees of freedom for estimating covariate effects (participating centres, left/right hemihepatectomy), a total sample size of 250 patients (125 per arm) can demonstrate a 2-day reduction in functional recovery with a level of significance alpha = 0.04, two-tailed and a power of 80% (see the calculation below).

2-day difference in functional recovery, 5 participating centres.

Effect size (d) = 2/5 = 0.40

Power = 80% → Zbeta = 0.84

Alfa = 4% → Zalfa = 2.05

n per arm = 2*((Zalfa + Zbeta)/d)**2

X = 2*((2.05+0.84)/(0.40))**2

X = 2*((2.89/0.40)**2

X = 105

N total = 105+105 = 210

* Corrections for loss in degrees of freedom, random effects, fixed effects: 5%
  N total = 210*1.05 = 221
* Corrections sequential analyses (O'Brien & Fleming), K = 2, factor 1.00625
  N total = 221*1.00625 = 222
* 10% drop-out
  N total = 222*(100/90) = 247

This number was rounded to **250 patients.**

4.4.2 Hemihepatectomy sample size at interim analysis

Revisiting the results at interim analysis (N = 112, 13 patients less than expected due to drop-outs), the effect size was lower than anticipated (*d* = 0.34). Also the amount of randomising centres is more than was calculated for. Based on the decreased effect size and the extra participating centres, we recalculated the sample size. A total sample size of 350 patients (175 per arm) can demonstrate a 2-day reduction in functional recovery with a level of significance alpha = 0.04, two-tailed and a power of 80% (see the calculation below).

Recalculated sample size: 2-day difference in functional recovery, 15 participating centres, effect size is 0.34 instead of 0.40.

Effect size = 0.34
Power = 80% → Zbeta = 0.84
Alfa = 4% → Zalfa = 2.05
n per arm = 2*((Zalfa + Zbeta)/d)**2
X = 2*((2.05 + 0.84)/(0.40)**2
X = 2*((2.89/0.34)**2
X = 2*72 = 144
N total = 144 + 144 = 288

* Corrections for loss in degrees of freedom, random effects, fixed effects: 5%
  N total = 288*1.05 = 302
* Corrections sequential analyses (O'Brien & Fleming), K = 2, factor 1.00625
  N total = 302*1.00625 = 305
* 10% drop-out
  N total = 305*(100/90) = **338**
* 10 extra centres: +10 = **348**

This can be rounded to **350 patients.**

4.4.3 One or two postero-superior liver segments 4A, 7, 8.

In a multicentre propensity score-matched analysis of laparoscopic versus open resections of postero-superior liver segments, patients operated with the conventional open procedure approach, the median hospital stay was 6 days (3-44 days) compared to 4 days (1-11 days) of that of patients undergoing laparoscopic liver resections.[44] Based on this information, an average reduction in hospital length of stay of 2 days (SD = 5) seems feasible and safe for patients undergoing laparoscopic surgery, in comparison to patients undergoing the open procedure. The sample size calculation would thus be identical to those for the hemihepactomy patients: a total sample size of 250 patients (125 per arm) can demonstrate a 2-day reduction with a level of significance α=0.04, two-tailed and a power of 80%. Note that a 3-day reduction in functional recovery days would give a total sample size of $125*(2/3)^2$ = 56 patients per arm.

5. TREATMENT OF SUBJECTS

In this interventional study we will evaluate the impact of laparoscopic and open hemihepatectomy (with or without the need for one additional hepatic wedge resection or metastasectomy) and laparoscopic and open parenchymal sparing postero-superior liver segment resection. All subjects will be treated within an enhanced recovery after surgery (ERAS®) programme. For both hemihepactectomy and parenchymal preserving postero-superior liver resections the open and laparoscopic surgical procedures as well as the ERAS® programme already exist as interventions that are part of current practice in the participating international centres.

5.1 Investigational product/treatment

All patients will be included within an ERAS® programme. Patients are only considered for randomization if the multidisciplinary team feel is technically feasible and safe by either approach for:

a) Hemihepatectomy (with or without the need for one additional hepatic wedge resection or metastasectomy).
b) Parenchymal sparing resection involving one or two of liver segments 4a, 7, 8. A segment 6/7 resection would be eligible as well.

If patients agree to participate in the trial, they will be randomized for the surgical intervention type. The patient will remain blinded up to and including postoperative day 4 (POD 4). Only in case of strong treatment preference from the operating surgeon, the patient can be included in a prospective registry and will not be blinded. This prospective registry will be maintained for both the hemihepatectomies and the postero-superior resections. Due to the administrative burden, the required time and the associated costs, the centres in Germany, Norway and the United Kingdom have decided not to add patients to the registries.

5.2 Use of co-intervention

Not applicable

5.3 Escape medication

Not applicable

6. INVESTIGATIONAL MEDICINAL PRODUCT

Not applicable.

6.1 Name and description of Investigational Medicinal Product

Not applicable.

6.2 Summary of findings from non-clinical studies

Not applicable.

6.3 Summary of findings from clinical studies

Not applicable.

6.4 Summary of known and potential risks and benefits

Not applicable.

6.5 Description and justification of route of administration and dosage

Not applicable.

6.6 Dosages, dosage modifications and method of administration

Not applicable.

6.7 Preparation and labelling of Investigational Medicinal Product

Not applicable.

6.8 Drug accountability

Not applicable.

7. NON-INVESTIGATIONAL PRODUCT

Not applicable.

7.1 Name and description of Non-Investigational Medicinal Product(s)

Not applicable.

7.2 Summary of findings from non-clinical studies

Not applicable.

7.3 Summary of findings from clinical studies

Not applicable.

7.4 Summary of known and potential risks and benefits

Not applicable.

7.5 Description and justification of route of administration and dosage

Not applicable.

7.6 Dosages, dosage modifications and method of administration

Not applicable.

7.7 Preparation and labelling of Non-Investigational Medicinal Product

Not applicable.

7.8 Drug accountability

Not applicable.

8. METHODS

8.1 Study parameters/endpoints

8.1.1 Main study parameter/endpoint

The primary endpoint of the ORANGE II PLUS – Trial is time to functional recovery. A patient is considered functionally recovered if the five following criteria are met. A patient needs:

- to have adequate pain control with oral analgesics;
- to be independently mobile; as objectified with an ERAS® Mobility Score (EMS) of 8 or higher (see *appendix 5);*
- to tolerate solid food;
- to have a normal or decreasing TB (total bilirubin), ALT (alanine aminotransferase), AST (aspartate aminotransferase) and a normal INR (international normalized ratio) or an INR of at least 80% of its normal value;
- to be independent of intravenous fluid administration.

It is medically justified to discharge patients when the criteria for full functional recovery are satisfied and if the patient is willing to go home (see *appendix 6*). The individual criteria are further specified in paragraph 8.3.1.

8.1.2 Secondary study parameters/endpoints

- Hospital length of stay
- Intraoperative blood loss
- Operation time
- (Liver specific) morbidity
- Readmission percentage
- Resection margin
- Quality of life
- Body image and cosmesis
- Reasons for delay of discharge after functional recovery
- Long term incidence of incisional hernias
- Hospital and societal costs during one year
- Time to adjuvant chemotherapy initiation
- Overall five-year survival

8.1.3 Other study parameters

Not applicable

8.2 Randomization, blinding and treatment allocation

The international multicentre ORANGE II PLUS - Trial is a prospective, double blinded, randomized controlled study of two separate and independent patient populations, each producing two arms (open versus laparoscopy) with a parallel registry (see *appendix 3*). These populations involve patients undergoing left or right hemihepatectomy and patients undergoing parenchymal preserving resection of one or two postero-superior liver segments (involving one or two of segments 4a, 7 or 8). All patients will be participating in an enhanced recovery programme. Participating centres (*appendix 7*) need to be able to perform and have adequate experience with the open and laparoscopic surgical techniques. To assure the quality of a procedure, surgeons can be allocated for proctoring.

Trial candidates who meet the inclusion criteria are informed of the study by their treating surgeon at the outpatient clinic of the surgical departments. Only after the patient has consented to be further informed about the study, he or she will be approached by the principal investigator, the authorized researcher, an informed surgical resident or a research nurse at the outpatient clinic of the surgical departments. After 1 week of reflection, a personal written informed consent will be obtained by the principal investigator, the authorized researcher or the research nurse. All patients ineligible for randomization are asked to participate in the prospective registry. The number of ineligible patients and the reasons for randomization ineligibility are registered in screening logs.

8.2.1 Randomization

The randomization procedure is performed with internet-based randomization software (TENALEA®, online randomization module, maintained by the Clinical Trial Center Maastricht). Randomization is based on minimization and will be stratified according to centre and either side (for hemihepactectomies: left or right) or tumour size (for parenchymal sparing postero-superior liver segment resections: tumours larger or smaller than 3 centimeters).

8.2.2 Blinding

The patient, ward physicians and nurses will be blinded to the type of intervention using a large abdominal dressing that covers the surgical incisions until postoperative day 4. Besides physical blinding, the operating schedules and surgical reports are also adjusted by the principal investigators to prevent any preliminary unblinding before day 4. The medical and nursing staff can be unblinded if this is necessitated by patients' condition, for instance because of severe wound leakage, infection or any other adverse event that requires

immediate medical attention. All unblindings and reasons for unblinding will be registered. The trial's blinding adequacy will be tested at the second day after surgery by asking the patient if open or laparoscopic surgery has been performed. Theoretically, a 50% correct overall score will be obtained if blinding has been executed perfectly.

8.3 Study procedures

8.3.1 Functional recovery

The primary endpoint of the ORANGE II PLUS – Trial is time to functional recovery. A patient is considered functionally recovered if the five following criteria are met. A patient needs:

- to have adequate pain control with oral analgesics;
- to be independently mobile, as objectified with an ERAS® Mobility Score (EMS) of 8 or higher;
- to tolerate solid food;
- to have a normal or decreasing TB (total bilirubin), ALT (alanine aminotransferase), AST (aspartate aminotransferase) and a normal INR (international normalized ratio) or an INR of at least 80% of its normal value;
- to be independent of intravenous fluid administration.

It is medically justified to discharge patients when the criteria for full functional recovery are satisfied and if the patient is willing to go home (see *appendix 6*).

Adequate pain control with oral analgesics

Postoperative pain will be systematically recorded twice daily using the validated verbally administered 0-10 NRS-11 (11 points numeric rating scale).[53-55] Members of a specialized pain team ask patients to rate the intensity of their current pain on a scale of 0 ("no pain") to 10 ("worst possible pain"). A score of 1-3 will be considered to be mild, 4-6 to be moderate and 7-10 to be severe.[56]

Tolerance for solid food

Fluid and solid food intake will be monitored and must be returned to normal tolerance level. Tolerance is considered normal when oral intake of water or normal food is resumed and continued for at least 24 hours. Furthermore, the postoperative nausea and vomiting (PONV), which obviously influences the intake, will be prophylactically countered (antiemetic) and also be registered postoperatively (until the 6th day after surgery) by use of a scale ranging from 0 ("no nausea") to 10 ("worst possible nausea").

Mobile at preoperative level

In order to report the difference between pre- and postoperative level of mobility, the EMS (see *appendix 5*), which is inferred from the ´Groningen activity restriction scale', was developed.[57] The EMS utilizes ten items of basic actions to compare the level of mobility before and after surgical intervention. When patients are able to perform 8 out of 10 items, they are considered independently mobile. Patients will have to report whether they are able to fully and independently perform these basic actions or not. Twice a day the assessment will be repeated and compared to the preoperative baseline score until mobility at an independent level is achieved.

8.3.2 Surgery

The intraoperative surgical technique in this study will not be standardized. Surgeons in the participating centres performing hemihepatectomies and parenchymal preserving postero-superior liver resections are free to use their preferred open and laparoscopic techniques and devices to gain intra-abdominal access, perform parenchymal transection and maintain vascular control. Intraoperative blood loss, vascular occlusion methods, Pringle time and total operation time will be monitored.

8.3.3 Laboratory

Preoperatively, venous blood samples will be drawn and analysed for blood group (Rhesus factor, irregular antibodies), haemoglobin, leucocytes, platelets, PT, aPTT, INR, renal function (Na^+, K^+, Urea, Creatinine), liver function (AF, Y-GT, AST, ALT, LDH, Bilirubin), CRP, CEA and AFP. In the postoperative days (PODs), venous blood samples will be drawn on POD 1, 2, 3, 5 and 7 (additional testing can be considered if indicated). Postoperative blood tests are identical to the preoperative samples, except the tumour marker concentrations will not be determined.

8.3.4 Morbidity

Patients' preoperative morbidity status is measured using the American Society of Anesthesiologists (ASA) physical status classification system. The Satava classification (*appendix 8*) adapted for liver surgery will be used to score unfavourable intraoperative incidents.[58, 59] Postoperative morbidity is rationally predictable; haemorrhagic

complications occurring predominantly during surgery or in the early postoperative phase, and biliary complications, intra-abdominal abscess or liver failure in the later postoperative phase. Also, wound infection and sepsis will be complications to monitor. All morbidity will be classified according to the Accordion Severity Grading System of Surgical Complications (see *appendix 9*) as described by Strasberg et al. and registered as adverse or serious adverse events.[60, 61]

8.3.5 Liver resection specific composite endpoint

In this trial, we have chosen to use a well-defined liver surgery-specific composite endpoint, as suggested by van den Broek et al.[62] This endpoint is a parameter composed of a combination of procedure-specific complications and considered as a single, dichotomous outcome: operative mortality, intra-abdominal haemorrhage, ascites, bile leakage, intra-abdominal abscess and postresectional liver failure. These components, which are all specific to liver surgery and have substantial clinical relevance, reflect Dindo grade 3-5 complications.

8.3.6 Pathology

Resection margin will be defined in both millimetres and status (R0 – R2) in all resected specimens as a secondary endpoint. The resected non-tumorous liver parenchyma will be analysed by an experienced pathologist in each participating centre for underlying liver disease. Level of fibrosis, inflammation and steatosis will be determined. The METAVIR score will reflect fibrosis (F0-F4) and inflammatory activity (A0-A3).[63] In addition, the level of steatosis (Grade 0 to 3) and presence of chemotherapy-induced hepatopathy (Sinusoidal Obstruction Syndrome = SOS, Chemotherapy-Associated Steatohepatitis = CASH) will be defined. This will enable stratification for underlying liver disease during data analysis.

8.3.7 Quality of life

To assess quality of life in patients undergoing laparoscopic surgery versus those undergoing open surgery, the Dutch version of the EQ-5D (EuroQol Group) status test and the translated EQ-5D for international centres will be used. EQ-5D (*appendix 10*) is a standardized instrument to measure health outcomes, which is divided in five dimensions (mobility, self-care, usual activities, pain/discomfort and anxiety/depression) with three levels each and an EQ-VAS score (0-100).[64-66] Furthermore, the EORTC QLQ-C30 (*appendix 11)* with the LM21 module (*appendix 12)* will be used for liver specific treatment measurements.[67] Assessment of the patients' quality of life will be performed pre- and

postoperatively: at hospital admission, hospital discharge and at 10 days, 3, 6 and 12 months after discharge.

8.3.8 Body image and cosmesis

In order to evaluate differences in postoperative body image and cosmesis, the Body image Questionnaire (BIQ) will be used (*appendix 13*).[68, 69] The BIQ consists of 8 questions regarding body image and cosmesis. The body image assessment will be performed pre- and postoperatively: at hospital admission, hospital discharge and at 10 days, 3, 6 and 12 months after discharge.

8.3.9 Long term incidence of incisional hernias

Incisional hernia after open surgery is a well-known complication with an incidence of up to 20% after a 10-year period.[70] In patients undergoing a sigmoid resection, Anderson et al. found that laparoscopic resection leads to a significantly lower incidence of incisional hernia compared with the open surgical technique.[71] The incidence of incisional hernia after open liver resection is reported to be 9.2% by Ito et al.[72] A randomized controlled trial by Halm et al. - comparing transversal versus midline incision for upper abdominal surgery - demonstrated an incisional hernia incidence of respectively 1.7% and 14.5%.[73] To assess the incidence of incisional hernia in open and laparoscopic hemihepatectomy, patients will be contacted at a mean time of 1 year after resection to receive an ultrasound or CT-scan to exclude incisional hernia.

8.3.10 Economic evaluation

(In)direct treatment costs

Liver surgery expenses constitute of personnel and material costs. Total operating time, operating theatre and material costs (e.g. disposables, monitors, endoscopic tower), personnel costs (surgeon, anaesthesiologist, assistants, operating nurses) and hospital stay will be documented. Intraoperative costs will be listed by operating nurses who will register the use of all instruments and other items. Unit prices will be based on prices from the participating hospital financial departments or, if unavailable, will be derived from general national guidelines for pricing.

Pre-admission charges for screening are not considered, as these are equal for both surgical techniques. All readmission related costs will be added to the total hospital expenses.

Furthermore, postoperative outpatient clinic visits, general practitioner consultations, and home care related costs will be documented and quantified. A cost questionnaire offered at regular follow-up consultations (3, 6 and 12 months) will help us to assess the societal and individual costs due to patients' work absence and the impact of the surgery on work and normal daily activities (*appendix 14*).

Societal Costs and Cost-Utility Analysis

For patients performing paid labour, productivity loss will be calculated using the human-capital approach, which counts any hour not worked as an hour lost. Preoperative assessment will document the type of occupation and a full- or part-time employment. The incremental costs per Quality Adjusted Life Year (QALY) gained will be based on utility scores from the EQ-5D combined with the total (direct and indirect) patient costs.[64-66]

8.3.11 Oncological endpoints

When admitted, the preoperative use of neo-adjuvant treatment is registered. During follow-up, the time frame between the liver resection and the start of adjuvant chemotherapy (if indicated) and the 5-year overall survival are monitored.

8.4 Withdrawal of individual subjects

Subjects can leave the study at any time for any reason if they wish to do so, without any consequences.

8.5 Replacement of individual subjects after withdrawal

Subjects will only be replaced after withdrawal if the withdrawal takes places before the patient is functionally recovered (primary endpoint). A dropout rate of 10% has been taken into account for the power calculation of the primary endpoint. Exceeding of the dropout rate will be corrected by prolonged inclusion to obtain the necessary number of participants. Patients will not be withdrawn from the study if the recovery is not within the anticipated time frame.

8.6 Follow-up of subjects withdrawn from treatment

As required by the CONSORT statement, analysis of all patients will be performed using the intention-to-treat (ITT) principle: all randomized patients will be analysed according to their initial treatment assignment.[74] For example, the small number of patients who require an

unplanned hand-assisted procedure, or for whom the liver resection procedure is altered intraoperatively, will remain in their randomization arm. Moreover, the surgical procedure itself may be stopped or even cancelled after randomization (e.g. due to unforeseen extrahepatic metastases); these patients will be regarded as ITT. In addition, also whenever the blinding cannot be continued until POD 4, patients will be seen as ITT. All ITT patients will be included in the final data analysis with proper methods for handling missing data.

8.7 Premature termination of the study

Reasons for a premature termination of the trial are the inability to randomise patients, significant differences between two groups during interim analysis or significant differences between the groups regarding the occurrence of serious adverse events.

8.8 Section 10 WMO event

In accordance with section 10, subsection 1, of the WMO, the investigator will inform the subjects and the reviewing accredited METC if anything occurs, on the basis of which it appears that the disadvantages of participation may be significantly greater than was foreseen in the research proposal. The study will be suspended pending further review by the accredited METC, except if a suspension would jeopardise the subjects' health. The investigator will take care that all subjects are kept informed.

8.9 AEs, SAEs and SUSARs

8.9.1 Adverse events (AE's)

Adverse events are defined as any undesirable experience occurring to a subject during a clinical trial, whether or not related to the investigational drug. All adverse events reported spontaneously by the subject or observed by the investigator or clinical staff will be recorded for one year according to the Accordion Severity Grading System of Surgical Complications (see *appendix 9*).[60, 61]

8.9.2 Serious adverse events (SAEs)

Adverse events are regarded serious if they:

- lead to death;
- are life threatening (e.g. intensive care admittance);
- lead to and admission longer than 10 days;

- lead to a readmission within 30 days after surgery,
- lead to permanent or serious invalidity.

All serious adverse events are announced within 24 hours to the coordinating investigator of the Maastricht University Medical Center+ by fax or email and will be reported through the web portal *ToetsingOnline* to the research ethics committee. SAEs that result in death or are life-threatening should undergo expedited reporting. The expedited reporting will occur not later than 7 days after the responsible investigator becomes aware of the adverse reaction. This is for a preliminary report with another 8 days for completion of the report.

The responsibilities of investigators in participating centres as well as of the coordinating investigator (Sponsor) in this multicentre study are outlined below.

	Responsibility to:	Responsible Party:	If Responsibility is delegated, name the body / individual that it is delegated to:
**Adverse events**	a) Maintain detailed records of all adverse events as specified in the protocol.	Sponsor and local organization	Local Clinical Trials Unit (on behalf of Sponsor)
	b) Report adverse events as agreed in the protocol and to legal requirements and in accordance with local organization policy.	Sponsor and local organization	Local Clinical Trials Unit (on behalf of Sponsor)
	c) Ensure that procedures are in place for emergency unblinding of the randomization code.	Sponsor	Local Clinical Trials Unit (on behalf of Sponsor)
	d) Promptly inform regulatory authorities, ethics committees and investigators of any urgent safety measures taken to protect participants in the study.	Sponsor	Local Clinical Trials Unit (on behalf of Sponsor)
	e) Ensure that annual safety reports and end of study reports are generated and submitted to the regulatory authority and relevant ethics committee within the required timeframes.	Sponsor	Local Clinical Trials Unit (on behalf of Sponsor)
	f) Ensure that all investigators are, at all times, in possession of the current relevant safety information of the Study.	Sponsor	Local Clinical Trials Unit (on behalf of Sponsor)

Either party may suspend their involvement in the study pending the outcome of an investigation of a serious adverse event or otherwise on grounds of participant safety.

8.9.3 Suspected unexpected serious adverse reactions (SUSARs)

Not applicable.

8.10 Annual safety report

Not applicable.

8.11 Follow-up of adverse events

All adverse events will be followed until they have abated, or until a stable situation has been reached. Depending on the event, follow-up may require additional tests or medical procedures as indicated, and/or referral to the general physician or a medical specialist. The reporting requirement for SAEs affecting participants applies for all events occurring up to 30 days post-surgery.

8.12 Data Safety Monitoring Board (DSMB)

For this trial a DSMB is appointed that consists of three members: a chair, a statistician and a medical specialist. In a concerted effort a DSMB Charter has been developed and all three members have signed a non-competing interest form. The main responsibility of the DSMB is to safeguard the interests of trial participants, assess the safety and efficacy of the interventions during the trial, and monitor the overall conduct of the clinical trial.

9. STATISTICAL ANALYSIS

9.1 Descriptive statistics

Time to functional recovery and hospital length of stay are presented in days (mean, median, standard deviation, range). Morbidity will be classified according to the Accordion Severity Grading System of Surgical Complications and defined as a dichotomous composite endpoint. Readmission percentage will be given as a mean with standard deviation. All other outcomes and baseline characteristics will be summarized by descriptive statistics per treatment arm of the RCT. This procedure will be repeated for the non-randomized study part, using significance tests of baseline group differences to detect the most important candidate confounders.

9.2 Inferential analysis

The primary outcome measure "time to functional recovery" will be measured in days and will be analysed with fixed effects linear regression, taking into account treatment, centre, hemihepatectomy side (hemihepatectomies), tumour size (parenchynmal sparing postero-superior liver segment resections), patient age and gender, and using $\alpha = 0.04$ two-tailed for the treatment effect in view of the interim analysis. All other outcomes will be similarly analysed with regression (linear for quantitative outcomes, logistic for binary outcomes), applying a two-tailed alpha = 0.01 to adjust for multiple testing. Repeatedly measured outcomes will be analysed with mixed regression for repeated measures in order to include persons with missing data. Morbidity will be classified following the Accordion Severity Grading System of Surgical Complications and will only be presented as raw data since the required sample size for intervention effects on morbidity is much larger than the calculated sample size for this trial.[75]

Treatment by covariate interactions will be explored and - if found - will lead to subgroup analyses. These are secondary analyses however, in view of the lower power for interaction effects and the increased risk of type I errors due to multiple testing.

Finally, all analyses will be repeated for the hemihepatectomy and postero-superior liver segment resection registries and the results will be compared with those from the RCT.

9.3 Interim analysis

An interim analysis of the primary outcome and mortality will be performed after inclusion of 50% of the sample, using two general stopping rules and one specific stopping rule for the parenchymal sparing postero-superior liver segment resections:

9.3.1 Stopping for significance, 2-day difference in functional recovery

The trial will be stopped if an interim analysis after 50% of the needed randomized sample size (N = 125) shows a significant difference between both trial arms with respect to the primary outcome, time to functional recovery, at a two-tailed alpha of 0.01. Otherwise, the trial will be continued.

9.3.2 Stopping for safety

The trial will be stopped if the mortality after liver surgery in patients with a normal liver is greater than 5% or greater than 10% in cirrhotic patients at interim analysis when 50% of the needed randomized sample size (N = 125) has been included (irrespective of the trial arm).

9.3.3 Stopping for significance, 3-day difference in functional recovery (postero-superior liver segment resection only)

The trial will be stopped if there is a significant difference between both trial arms of the postero-superior liver segment resection cohort of the trial after 100% of the needed randomized sample size needed for a 3-day difference in functional recovery (N = 110, see sample size calculation). Otherwise – and if the trial should not be stopped for any of the other two reasons - the trial will continue randomization to N = 250, which is the sample size based on a 2-day difference in functional recovery.

The primary goal is to look for a 2-day reduction in functional recovery, but this setup gives the freedom to stop the trial if it is very successful, e.g. if the reduction to functional recovery turns out to be 3 days instead of 2 days.

10. ETHICAL CONSIDERATIONS

10.1 Regulation statement

This study will be conducted according to the principles of the Declaration of Helsinki (Seoul, 2008) and in accordance with the Medical Research Involving Human Subjects Act (WMO).

10.2 Recruitment and consent

Patients are considered for hemihepatectomy or parenchymal sparing postero-superior liver segment resection only after careful evaluation by a multidisciplinary board that includes surgeons, radiologists, pathologists, hepathologists, oncologists and radiotherapists. At these multidisciplinary meetings, all specific in- and exclusion criteria for trial participation are assessed.

Trial candidates who meet the inclusion criteria are informed of the study by their treating surgeon at the outpatient clinic of the surgical departments. Only after the patient has consented to be further informed about the study, he or she will be approached by the principal investigator, the authorized researcher, an informed surgical resident or a research nurse at the outpatient clinic of the surgical departments. After 1 week of reflection, a personal written informed consent will be obtained by the principal investigator, the authorized researcher or the research nurse. All patients ineligible for randomization are asked to participate in the prospective registry. The number of ineligible patients and the reasons for randomization ineligibility are registered in screening logs.

10.3 Objection by minors or incapacitated subjects

Not applicable.

10.4 Benefits and risks assessment, group relatedness

The risks associated with participation in the trial concerning the intervention (laparoscopic liver surgery) are comparable with the normal treatment (open liver surgery). As outlined in the introduction, when performed in expert centres, both laparoscopic hemihepatectomy and postero-superior liver resections have proven to feasible with good efficacy and safety outcomes.

Potential risks for the participating subjects, besides the standard surgical risks of infection,

bleeding and injury / perforation of an adjacent organ, are mostly related to the procedure:

- Postoperative pain from the abdominal incisions. A dedicated pain team will monitor the patients daily while they are in-patients;
- The carbon dioxide, used to inflate the abdomen for the laparoscopic procedure could lead to shoulder or back pain. Rest and analgesia will reduce this pain;
- Postoperative fatigue and reduced strength. A liver resection is a major surgical procedure and the human body needs time to fully recover to a preoperative level;
- Postoperative liver failure. Although every patient will be discussed in a multidisciplinary oncology meeting to ensure the feasibility of the hepatic resection. In case of a hemihepatectomy (thus not in case of a parenchymal preserving postero-superior liver segment resection) the remnant liver could be insufficient in volume and function, and this could potentially lead to liver failure;
- Conversion. In case of intra-operative complications, such as bleeding and insufficient progress, a laparoscopic procedure sometimes needs to be converted to an open procedure;

Several measures have been taken to reduce potential risks for participants in this study:
- The predefined in- and exclusion criteria ensure that patients are not subjected to increased risk of developing postoperative morbidity or mortality. These criteria clearly describe the eligible population;
- All participating centres are renowned hospitals for liver surgery and all operating surgeons have extensive experience with both minor, major, laparoscopic and open liver surgery (thus including hemihepatectomies and parenchymal preserving postero-superior liver segment resections);
- All patients are discussed preoperatively in a multidisciplinary team with surgeons, radiologists, pathologists, hepathologists, oncologists and radiotherapists;
- All perioperative care is standardized according to an evidence-based enhance recovery programme;
- During admission, patients will be more intensively monitored compared with standard care. Not only will we perform additional blood analyses from clinical blood samples during the first 9 postoperative days, nurses and physicians will also closely monitor the recovery of the patient applying the standardized functional recovery criteria and postoperative milestone checklists;
- All adverse events will be monitored and all SAEs will be reported to both the local and central research ethical committees;

- A DSMB has been formed to safeguard the interests of trial participants, assess the safety and efficacy of the interventions during the trial, and monitor the overall conduct of the clinical trial;
- Study contracts between each participating centres have been signed to ensure adequate delegation of responsibilities and liability.

The burden for participating subjects, which consists of blinding, completing quality of life, body image, cost questionnaires and having an additional ultrasound is in proportion with the potential value of this particular study.

10.5 Compensation for injury

The Sponsor/investigator has a liability insurance which is in accordance with article 7, subsection 6 of the WMO. A copy of the insurance certificate is available at the METC of the Maastricht University Medical Center+. Participating national and international centres will provide their patients with their own insurance, which as been subject to a local feasibility test according to the guidelines for External Audit.

The Sponsor (also) has insurance, which is in accordance with the legal requirements in the Netherlands (Article 7 WMO and the Measure regarding Compulsory Insurance for Clinical Research in Humans of 23rd June 2003). This insurance provides cover for damage to research subjects through injury or death caused by the study.

1. € 450.000,-- (i.e. four hundred and fifty thousand Euro) for death or injury for each subject who participates in the Research;
2. € 3.500.000,-- (i.e. three million five hundred thousand Euro) for death or injury for all subjects who participate in the Research;
3. € 5.000.000,-- (i.e. five million Euro) for the total damage incurred by the organization for all damage disclosed by scientific research for the Sponsor as 'verrichter' in the meaning of said Act in each year of insurance coverage.

The insurance applies to the damage that becomes apparent during the study or within 4 years after the end of the study.

10.6 Incentives

Not applicable.

11. ADMINISTRATIVE ASPECTS AND PUBLICATION

11.1 Handling and storage of data and documents

Data concerning patient characteristics, functional recovery, surgical/ anaesthesiological parameters, morbidity, length of stay, quality of life, patient compliance and costs will be prospectively collected using OpenClinica® (Ikaza Research, Cambridge, Massachusets, USA) and stored in a secured Oracle database (Oracle Corporation, Redwood Shores, CA, USA). OpenClinica is an open source clinical trial software platform, that uses e-CRF's, for electronic data capture (EDC) and clinical data management (CDM), validated and stored in compliance with good clinical practice (GCP) guidelines. All patient data is encoded to ensure the patient's anonymity. Data will be kept for at least 15 years after the end of the study.

11.2 Monitoring and Quality Assurance

A DSMB will monitor;

- patient safety
- quality of performance within the trial (trial conduct)
- stopping rules evaluation (superior / inferior effect)

The DSMB will evaluate the report after every 50 (randomized) patients and the interim analysis. Specific details regarding the monitoring and quality assurance can be found in the DSMB Charter. In addition, some participating countries also require local monitoring of the trial. Any report or decision from other monitoring bodies will be made available to the DSMB.

11.3 Amendments

Substantial changes to the research protocol have to be assessed by the accredited METC and may only be implemented after the METC has approved the changes.

All non-substantial amendments will be notified to the METC.

11.4 Annual progress report

The Sponsor/investigator will submit a summary of the progress of the trial to the accredited METC once a year. Information will be provided on the date of inclusion of the first subject, numbers of subjects included and numbers of subjects that have completed the trial, serious adverse events, other problems, and amendments.

11.5 End of study report

The investigator will notify the accredited METC of the end of the study within 8 weeks. The end of the study is defined as the last patient's last visit.
In case the study is ended prematurely, the investigator will notify the accredited METC, including the reasons for the premature termination.

Within one year of the end of the study, the investigator/Sponsor will submit a final study report with the results of the study, including any publications/abstracts of the study, to the accredited METC.

11.6 Public disclosure and publication policy

All data, positive and negative will be published. After extensive discussion within the Trial group and considering the international publication policy guidelines of the EORTC, IMCJE and CONSORT, the authorships have been distributed as follows. However, the final authorship division may be bound by journal regulations. All author staff involved per centre will at least be listed as 'collaborator in PubMed'. Secondary authors are allocated by the centres themselves.

1st author:	Principal Investigator major contributing center
Shared 1st author:	ORANGE PLUS team study coordinator
2nd author:	Principal Investigator second contributing center
3rd author:	Principal Investigator third contributing center
4th author:	ORANGE PLUS team study coordinator
First row authors:	Principal Investigators of the centres ranked according to the contribution (4th, 5th, 6th etc.)
Second row authors:	Secondary authors ranked according to the contribution (if >15 patients randomized and depending on journal regulations)
Third row authors:	Trial unit members coordinating centres
Shared last author:	ORANGE PLUS team Principal Investigator
Last author:	ORANGE PLUS study initiator and Principal Investigator

12. STRUCTURED RISK ANALYSIS

12.1 Potential issues of concern

a. **Level of knowledge about mechanism of action**

Not applicable**.**

b. **Previous exposure of human beings with the test product(s) and/or products with a similar biological mechanism**

Not applicable.

c. **Can the primary or secondary mechanism be induced in animals and/or in ex-vivo human cell material?**

Not applicable.

d. **Selectivity of the mechanism to target tissue in animals and/or human beings**

Not applicable.

e. **Analysis of potential effect**

Not applicable.

f. **Pharmacokinetic considerations**

Not applicable.

g. **Study population**

Not applicable.

h. **Interaction with other products**

Not applicable.

i. **Predictability of effect**

Not applicable.

j. **Can effects be managed?**

Not applicable.

13. SYNTHESIS

Optimising patient recovery is something both laparoscopic surgery and multimodal enhanced recovery programmes aim to achieve. One and the other may actually complement each other, possibly resulting in an additive or even synergistic effect. Laparoscopic liver resection is supposed to hold many benefits over conventional open liver resection, such as less postoperative pain and analgesic requirements, faster recovery, shorter hospital length of stay, smaller scars with preservation of the abdominal wall, lower transfusion requirements, and fewer postoperative adhesions.[45] High-volume liver surgery expert centres in the United Kingdom, France, Norway and United States of America already perform up to 60% of their hepatic resections laparoscopically.[45, 76] Therefore, it is attractive to centres with limited experience in the laparoscopic approach to adopt this technique. However, laparoscopic liver surgery should be implemented cautiously, as it also has considerable learning curves and seems more expensive.[77-79]

At the first international consensus conference on laparoscopic liver resections (ICCLLR) in 2008 in Louisville, laparoscopic left lateral sectionectomies were considered standard practice, whereas major laparoscopic liver resections were only to be reserved for experienced surgeons in high volume expert centres. In the following years, multiple studies indicated that major laparoscopic liver resections could be performed safely, although higher level evidence was required to value patient and economic outcomes, major hepatectomy-specific and long-term oncologic outcomes. This necessity was stressed again 6 years later at the second ICCLLR in Morioka in 2014, where the development of randomized trials for major laparoscopic surgery was strongly recommended.[46]

Despite the necessity to conduct an RCT in major laparoscopic liver surgery has been accentuated, its feasibility has been questioned by many publications, stating it to be too difficult to conduct, to be logistically not attainable, to be impractical or to just take too much time to complete.[80] Nevertheless, RCT's in liver surgery have actually proven to be plausible, if rightly timed and with properly chosen endpoints.[81, 82]

Indeed, a trial focussing on hepatic surgery-related mortality or morbidity as its primary endpoint seems futile due to the enormous sample size that would be required. In this light, hospital length of stay is a frequently used alternative outcome parameter, yet an endpoint that is difficult to use in a multicentre trial, as the gap between the moment a patient is functionally recovered and actual hospital discharge is prone to variation.[49, 83] This is why the ORANGE II PLUS – Trial opts for time to functional recovery as its primary outcome

measure; with clearly defined prerequisites and less external influences it is a more reliable and relevant outcome. Hospital length of stay is thus part of the secondary outcomes. Other secondary outcomes include intraoperative surgical parameters such as operation time and blood loss and more oncologically orientated results like resection margins, time needed to first adjuvant chemotherapy, disease free survival, overall survival and quality of life.

Formally, a randomized controlled trial requires standardization of the intervention and its environment. These preconditions can be hard to recreate in a surgical trial and thus may need to be addressed in a more pragmatic way. For instance, even though all participating hospitals are qualified and high-volume liver surgery centres with proficient practice in open and laparoscopic hemihepactomies and parenchymal preserving postero-superior liver segment resections, surgical experience and technique between surgeons and centres will vary. On the contrary, this setup will actually reflect current day surgical practice more precisely and is thus likely to strengthen the trial's generalisability.

Recapitulating all, the international multicentre ORANGE II PLUS - Trial is a prospective, double blinded, randomized controlled study of two separate and independent patient populations, each producing two arms (open versus laparoscopy) with a parallel registry. These populations involve patients undergoing left or right hemihepatectomy and patients having parenchymal preserving resection of one or two postero-superior liver segments (involving one or two of segments 4a, 7 or 8). The trial has been designed to compare the clinically relevant 'time to functional recovery' as its primary outcome and to assess multiple intra- and postoperative secondary outcomes including complications, a composite endpoint of liver-specific morbidity, oncological outcomes, quality of life and costs. The parallel prospective registries of patients who are ineligible for or refusing randomization is appended to increase the external validity.

14. REFERENCES

1. Scheele, J., R. Stangl, and A. Altendorf-Hofmann, *Hepatic metastases from colorectal carcinoma: impact of surgical resection on the natural history.* Br J Surg, 1990. **77**(11): p. 1241-6.
2. Stangl, R., et al., *Factors influencing the natural history of colorectal liver metastases.* Lancet, 1994. **343**(8910): p. 1405-10.
3. Lodge, J.P., *Modern surgery for liver metastases.* Cancer Imaging, 2000. **1**: p. 77-85.
4. Rees, M. and T.G. John, *Current status of surgery in colorectal metastases to the liver.* Hepatogastroenterology, 2001. **48**(38): p. 341-4.
5. Choti, M.A., et al., *Trends in long-term survival following liver resection for hepatic colorectal metastases.* Ann Surg, 2002. **235**(6): p. 759-66.
6. Simmonds, P.C., et al., *Surgical resection of hepatic metastases from colorectal cancer: a systematic review of published studies.* Br J Cancer, 2006. **94**(7): p. 982-99.
7. Wei, A.C., et al., *Survival after hepatic resection for colorectal metastases: a 10-year experience.* Ann Surg Oncol, 2006. **13**(5): p. 668-76.
8. Rees, M., et al., *Evaluation of long-term survival after hepatic resection for metastatic colorectal cancer: a multifactorial model of 929 patients.* Ann Surg, 2008. **247**(1): p. 125-35.
9. Pawlik, T.M., R.D. Schulick, and M.A. Choti, *Expanding Criteria for Resectability of Colorectal Liver Metastases.* The Oncologist, 2008. **13**(1): p. 51-64.
10. Schiesser, M., et al., *Perioperative morbidity affects long-term survival in patients following liver resection for colorectal metastases.* J Gastrointest Surg, 2008. **12**(6): p. 1054-60.
11. de Jong, M.C., et al., *Rates and patterns of recurrence following curative intent surgery for colorectal liver metastasis: an international multi-institutional analysis of 1669 patients.* Ann Surg, 2009. **250**(3): p. 440-8.
12. Morris, E.J., et al., *Surgical management and outcomes of colorectal cancer liver metastases.* Br J Surg, 2010. **97**(7): p. 1110-8.
13. Jones, R.P., et al., *Systematic review and meta-analysis of follow-up after hepatectomy for colorectal liver metastases.* Br J Surg, 2012. **99**(4): p. 477-86.
14. van Dam, R.M., et al., *Outcomes of extended versus limited indications for patients undergoing a liver resection for colorectal cancer liver metastases.* HPB (Oxford), 2014. **16**(6): p. 550-9.
15. Castaing, D., et al., *Oncologic results of laparoscopic versus open hepatectomy for colorectal liver metastases in two specialized centers.* Ann Surg, 2009. **250**(5): p. 849-55.
16. Dagher, I., et al., *Laparoscopic major hepatectomy: an evolution in standard of care.* Ann Surg, 2009. **250**(5): p. 856-60.
17. Nguyen, K.T., et al., *Minimally invasive liver resection for metastatic colorectal cancer: a multi-institutional, international report of safety, feasibility, and early outcomes.* Ann Surg, 2009. **250**(5): p. 842-8.
18. Cuesta, M.A., et al., *Limited laparoscopic liver resection of benign tumors guided by laparoscopic ultrasonography: report of two cases.* Surg Laparosc Endosc, 1995. **5**(5): p. 396-401.
19. Gagner, M., M. Rheault, and J. Dubuc, *Laparoscopic partial hepatectomy for liver tumor.* Surg Endosc, 1992. **6**: p. 97-8.
20. Hashizume, M., et al., *Laparoscopic hepatic resection for hepatocellular carcinoma.* Surg Endosc, 1995. **9**(12): p. 1289-91.
21. Rau, H.G., et al., *Laparoscopic liver resection with the water-jet dissector.* Surg Endosc, 1995. **9**(9): p. 1009-12.
22. Reich, H., et al., *Laparoscopic excision of benign liver lesions.* Obstet Gynecol, 1991. **78**(5 Pt 2): p. 956-8.
23. Carswell, K.A., et al., *Laparoscopic versus open left lateral segmentectomy.* BMC Surg, 2009. **9**: p. 14.

24. Vigano, L., et al., *Laparoscopic liver resection: a systematic review.* J Hepatobiliary Pancreat Surg, 2009. **16**(4): p. 410-21.
25. Cherqui, D., *Laparoscopic liver resection.* Br J Surg, 2003. **90**(6): p. 644-6.
26. Abu Hilal, M. and N.W. Pearce, *Laparoscopic left lateral liver sectionectomy: a safe, efficient, reproducible technique.* Dig Surg, 2008. **25**(4): p. 305-8.
27. Dagher, I., et al., *Laparoscopic liver resection for hepatocellular carcinoma.* Surg Endosc, 2008. **22**(2): p. 372-8.
28. Dagher, I., et al., *Laparoscopic liver resection: results for 70 patients.* Surg Endosc, 2007. **21**(4): p. 619-24.
29. Lee, K.F., et al., *Laparoscopic versus open hepatectomy for liver tumours: a case control study.* Hong Kong Med J, 2007. **13**(6): p. 442-8.
30. O'Rourke, N., et al., *Laparoscopic resection of hepatic colorectal metastases.* HPB (Oxford), 2004. **6**(4): p. 230-5.
31. Troisi, R., et al., *The value of laparoscopic liver surgery for solid benign hepatic tumors.* Surg Endosc, 2008. **22**(1): p. 38-44.
32. Abu Hilal, M., et al., *Laparoscopic versus open left lateral hepatic sectionectomy: A comparative study.* Eur J Surg Oncol, 2008. **34**(12): p. 1285-8.
33. Laurence, J.M., et al., *Laparoscopic hepatectomy, a systematic review.* ANZ J Surg, 2007. **77**(11): p. 948-53.
34. Alkari, B., A. Owera, and B.J. Ammori, *Laparoscopic liver resection: preliminary results from a UK centre.* Surg Endosc, 2008. **22**(10): p. 2201-7.
35. Mirnezami, R., et al., *Short- and long-term outcomes after laparoscopic and open hepatic resection: systematic review and meta-analysis.* HPB (Oxford), 2011. **13**(5): p. 295-308.
36. Hüscher, C.G., et al., *Current position of advanced laparoscopic surgery of the liver.* J R Coll Surg Edinb, 1997. **42**(4): p. 219-25.
37. Samama, G., et al., *Laparoscopic anatomical hepatic resection. Report of four left lobectomies for solid tumors.* Surg Endosc, 1998. **12**(1): p. 76-8.
38. O'Rourke, N. and G. Fielding, *Laparoscopic right hepatectomy: surgical technique.* J Gastrointest Surg, 2004. **8**(2): p. 213-6.
39. Nguyen, K.T., et al., *Comparative benefits of laparoscopic vs open hepatic resection: a critical appraisal.* Arch Surg, 2011. **146**(3): p. 348-56.
40. Gayet, B., et al., *Totally laparoscopic right hepatectomy.* Am J Surg, 2007. **194**(5): p. 685-9.
41. Topal, B., R. Aerts, and F. Penninckx, *Laparoscopic intrahepatic Glissonian approach for right hepatectomy is safe, simple, and reproducible.* Surg Endosc, 2007. **21**(11): p. 2111.
42. Reddy, S.K., A. Tsung, and D.A. Geller, *Laparoscopic liver resection.* World J Surg, 2011. **35**(7): p. 1478-86.
43. Abu Hilal, M., et al., *Single-centre comparative study of laparoscopic versus open right hepatectomy.* J Gastrointest Surg, 2011. **15**(5): p. 818-23.
44. Scuderi, V., et al., *Outcome after laparoscopic and open resections of posterosuperior segments of the liver.* Br J Surg, 2017.
45. Nguyen, K.T., T.C. Gamblin, and D.A. Geller, *World review of laparoscopic liver resection-2,804 patients.* Ann Surg, 2009. **250**(5): p. 831-41.
46. Wakabayashi, G., et al., *Recommendations for laparoscopic liver resection: a report from the second international consensus conference held in Morioka.* Ann Surg, 2015. **261**(4): p. 619-29.
47. van Dam, R.M., et al., *Initial experience with a multimodal enhanced recovery programme in patients undergoing liver resection.* Br J Surg, 2008. **95**(8): p. 969-75.
48. Stoot, J.H., et al., *The effect of a multimodal fast-track programme on outcomes in laparoscopic liver surgery: a multicentre pilot study.* HPB (Oxford), 2009. **11**(2): p. 140-4.
49. Maessen, J., et al., *A protocol is not enough to implement an enhanced recovery programme for colorectal resection.* Br J Surg, 2007. **94**(2): p. 224-31.

50. Chan, A.W., et al., *SPIRIT 2013 explanation and elaboration: guidance for protocols of clinical trials.* BMJ, 2013. **346**: p. e7586.
51. Dagher, I., et al., *Laparoscopic versus open right hepatectomy: a comparative study.* Am J Surg, 2009. **198**(2): p. 173-7.
52. Jarnagin, W.R., et al., *Improvement in perioperative outcome after hepatic resection: analysis of 1,803 consecutive cases over the past decade.* Ann Surg, 2002. **236**(4): p. 397-406; discussion 406-7.
53. Paice, J.A. and F.L. Cohen, *Validity of a verbally administered numeric rating scale to measure cancer pain intensity.* Cancer Nurs, 1997. **20**(2): p. 88-93.
54. McGuire, D.B., *Comprehensive and multidimensional assessment and measurement of pain.* J Pain Symptom Manage, 1992. **7**(5): p. 312-9.
55. Dorrepaal, K.L., N.K. Aaronson, and F.S. van Dam, *Pain experience and pain management among hospitalized cancer patients. A clinical study.* Cancer, 1989. **63**(3): p. 593-8.
56. Krebs, E.E., T.S. Carey, and M. Weinberger, *Accuracy of the pain numeric rating scale as a screening test in primary care.* J Gen Intern Med, 2007. **22**(10): p. 1453-8.
57. Suurmeijer, T.P., et al., *The Groningen Activity Restriction Scale for measuring disability: its utility in international comparisons.* Am J Public Health, 1994. **84**(8): p. 1270-3.
58. Satava, R.M., *Identification and reduction of surgical error using simulation.* Minim Invasive Ther Allied Technol, 2005. **14**(4): p. 257-61.
59. Kazaryan, A.M., et al., *Comparative evaluation of laparoscopic liver resection for posterosuperior and anterolateral segments.* Surg Endosc, 2011. **25**(12): p. 3881-9.
60. Strasberg, S.M., D.C. Linehan, and W.G. Hawkins, *The accordion severity grading system of surgical complications.* Ann Surg, 2009. **250**(2): p. 177-86.
61. Porembka, M.R., et al., *Quantitative weighting of postoperative complications based on the accordion severity grading system: demonstration of potential impact using the american college of surgeons national surgical quality improvement program.* J Am Coll Surg, 2010. **210**(3): p. 286-98.
62. van den Broek, M.A., et al., *Development of a composite endpoint for randomized controlled trials in liver surgery.* Br J Surg, 2011. **98**(8): p. 1138-45.
63. Bedossa, P. and T. Poynard, *An algorithm for the grading of activity in chronic hepatitis C. The METAVIR Cooperative Study Group.* Hepatology, 1996. **24**(2): p. 289-93.
64. Lamers, L.M., et al., *[Measuring the quality of life in economic evaluations: the Dutch EQ-5D tariff].* Ned Tijdschr Geneeskd, 2005. **149**(28): p. 1574-8.
65. Lamers, L.M., et al., *The Dutch tariff: results and arguments for an effective design for national EQ-5D valuation studies.* Health Econ, 2006. **15**(10): p. 1121-32.
66. Langenhoff, B.S., et al., *Quality of life after surgical treatment of colorectal liver metastases.* Br J Surg, 2006. **93**(8): p. 1007-14.
67. Blazeby, J.M., et al., *Validation of the European Organization for Research and Treatment of Cancer QLQ-LMC21 questionnaire for assessment of patient-reported outcomes during treatment of colorectal liver metastases.* Br J Surg, 2009. **96**(3): p. 291-8.
68. Dunker, M.S., et al., *Cosmesis and body image after laparoscopic-assisted and open ileocolic resection for Crohn's disease.* Surg Endosc, 1998. **12**(11): p. 1334-40.
69. Polle, S.W., et al., *Body image, cosmesis, quality of life, and functional outcome of hand-assisted laparoscopic versus open restorative proctocolectomy: long-term results of a randomized trial.* Surg Endosc, 2007. **21**(8): p. 1301-7.
70. Höer, J., et al., *Einflussfaktoren der Narbenhernienentstehung Retrospektive Untersuchung an 2.983 laparotomierten Patienten über einen Zeitraum von 10 Jahren.* Der Chirurg, 2002. **73**(5): p. 474-480.
71. Andersen, L.P., et al., *Incisional hernia after open versus laparoscopic sigmoid resection.* Surg Endosc, 2008. **22**(9): p. 2026-9.

72. Ito, K., et al., *Laparoscopic versus open liver resection: a matched-pair case control study.* J Gastrointest Surg, 2009. **13**(12): p. 2276-83.
73. Halm, J.A., et al., *Incisional hernia after upper abdominal surgery: a randomized controlled trial of midline versus transverse incision.* Hernia, 2009. **13**(3): p. 275-80.
74. Schulz, K.F., et al., *CONSORT 2010 statement: updated guidelines for reporting parallel group randomized trials.* BMJ, 2010. **340**: p. c332.
75. van den Broek, M.A., et al., *Feasibility of randomized controlled trials in liver surgery using surgery-related mortality or morbidity as endpoint.* Br J Surg, 2009. **96**(9): p. 1005-14.
76. Kazaryan, A.M., et al., *Laparoscopic liver resection for malignant and benign lesions: ten-year Norwegian single-center experience.* Arch Surg, 2010. **145**(1): p. 34-40.
77. Vigano, L., et al., *The learning curve in laparoscopic liver resection: improved feasibility and reproducibility.* Ann Surg, 2009. **250**(5): p. 772-82.
78. van der Poel, M.J., et al., *Outcome and Learning Curve in 159 Consecutive Patients Undergoing Total Laparoscopic Hemihepatectomy.* JAMA Surg, 2016. **151**(10): p. 923-928.
79. Stoot, J.H., et al., *The introduction of a laparoscopic liver surgery programme: a cost analysis of initial experience in a university hospital.* Scand J Surg, 2012. **101**(1): p. 32-7.
80. Buell, J.F., et al., *The International Position on Laparoscopic Liver Surgery.* Annals of Surgery, 2009. **250**(5): p. 825-830.
81. Fretland, A.A., et al., *Open versus laparoscopic liver resection for colorectal liver metastases (the Oslo-CoMet Study): study protocol for a randomized controlled trial.* Trials, 2015. **16**: p. 73.
82. Wong-Lun-Hing, E.M., et al., *Randomized clinical trial of open versus laparoscopic left lateral hepatic sectionectomy within an enhanced recovery after surgery programme (ORANGE II study).* Br J Surg, 2017. **104**(5): p. 525-535.
83. Hendry, P.O., et al., *Randomized clinical trial of laxatives and oral nutritional supplements within an enhanced recovery after surgery protocol following liver resection.* Br J Surg, 2010. **97**(8): p. 1198-206.

15. APPENDICES

Appendix 1: ERAS® major abdominal surgery components

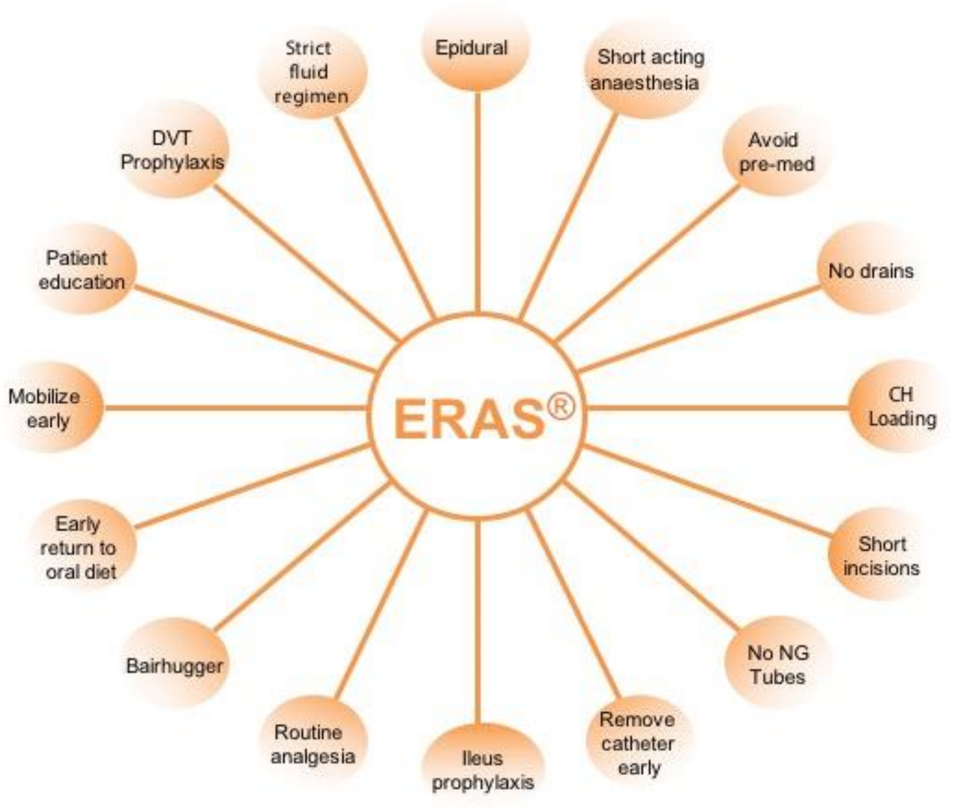

Appendix 2: Daily guidelines of care for patients undergoing hepatectomy in the ERAS® program

Day before surgery (POD-1)
- Normal oral nutrition up to 6 hours before surgery
- No standard anxiolytic premedication
- Laboratory tests

Day of surgery (POD0)
- Carbohydrate drinks up to 2 hours prior to surgery
- Balanced general anesthesia + PCIA or PCEA
- Preferably no nasogastric drainage, but when used remove after surgery
- Use warm IV-fluids and upper and lower body air-warming device
- Avoid excessive IV-fluids
- CVP <5 mmHg during transection
- Minimal incision / laparoscopy
- No routine drainage of the peritoneal cavity
- PONV + antithrombotic prophylaxis
- Patient sent to recovery ward
- Restart oral intake of water / nutrition

Postoperative day 1 (POD1)
- Patient sent to surgical ward
- Patient mobilizes a minimum of four times a day (sitting in chair / bedside)
- Discontinuation of IV-fluids
- Patient drinks at least 1.5 litres
- Normal diet
- Continue PCIA or PCEA
- Check functional recovery + discharge criteria
- 1000 mg aminocetophen 6 hourly
- Start laxatives
- Laboratory tests

Postoperative day 2 (POD2)
- Continue PCIA or PCEA
- Stop low dose opioids
- Continue mobilization (3 times 1 hour, walk)
- 1000 mg aminocethophen 6 hourly
- Normal diet
- Laxatives
- Check functional recovery +discharge criteria

Postoperative day 3 (POD3)
- Stop / continue PCIA or PCEA
- Remove urinary catheter (epidural)
- Start NSAIDs
- Continue mobilization (<6 hours)
- Normal diet
- Laxatives
- Laboratory tests
- Outpatient appointment made on postoperative day 10, 11 or 12
- Check functional recovery + discharge criteria

Appendix 3: ORANGE II PLUS – Trial flowchart

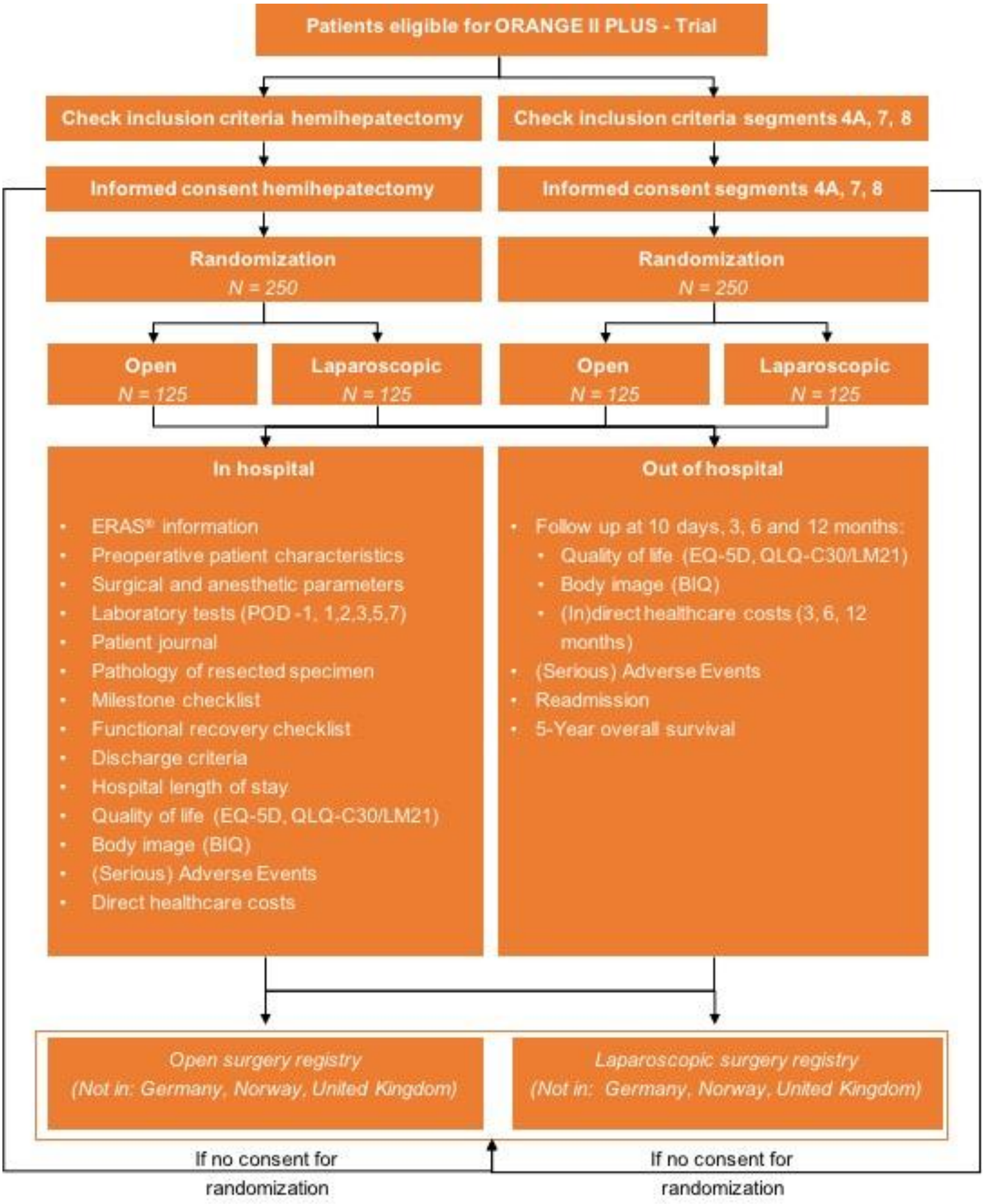

Appendix 4: SPIRIT flowchart

	STUDY PERIOD										
	Enrolment	Randomization	Post-allocation								Close-out
**TIMEPOINT**	**Screening**	**>1 week post screening**	**POD -1**	**Surgery (POD 0)**	**Admission**	**Discharge**	**Follow-up 10 days**	**Follow-up 3 months**	**Follow-up 6 months**	**Follow-up 12 months**	**5 years**
**ENROLLMENT**											
Eligibility screen	●										
Informed consent		●									
Randomization or registry		●									
**INTERVENTION**											
Open hemihepatectomy				●							
Laparoscopic hemihepatectomy				●							
**ASSESSMENT**											
Functional recovery				●	—	●					
Hospital length of stay				●	—	●					
Intraoperative blood loss				●							
Operation time				●							
Time to adjuvant chemotherapy						●	—	—	—	●	
Readmission percentage						●	—	—	—	●	
(Liver specific) morbidity				●	—	—	—	—	—	●	
Quality of life			●			●	—	—	—	●	
Body image			●			●	—	—	—	●	
Reasons for delay of discharge						●					
Incisional herniations						●	—	—	—	●	
Hospital and societal costs				●	—	—	—	—	—	●	
Ultrasound incisional herniation										●	
Five-year survival											●

Appendix 5: ERAS® Mobility Score (EMS)

Can you fully independently:		YES = 1 / NO = 0
1.	Feed yourself?	YES/NO
2.	Get in and out of bed?	YES/NO
3.	Stand up from sitting in a chair?	YES/NO
4.	Get on and off the toilet?	YES/NO
5.	Wash yourself?	YES/NO
6.	Dress yourself completely?	YES/NO
7.	Prepare breakfast or lunch (light meals)?	YES/NO
8.	Get around in the house/ward (if necessary with a crane)?	YES/NO
9.	Walk over 100 meters (not only inside home)?	YES/NO
10.	Go up and down the stairs?	YES/NO
TOTAL POINTS:		________

Appendix 6: Functional recovery checklist

	Functional Recovery	Discharge (LOS)
Tolerance for solid food	✔	✔
No intravenous fluids	✔	✔
Oral analgetics only	✔	✔
Mobile at preoperative level	✔	✔
Normal or improving serum bilirubine & INR	✔	✔
Willing to go home		✔

Appendix 7: ORANGE II PLUS – Trial participating centres

University Hospital Aachen
Academic Medical Center, Amsterdam
Queen Elizabeth Hospital, Birmingham
Erasmus Hospital, Brussels
Ghent University Hospital
Jessa Hospital, Hasselt
General Hospital Groeninge, Kortrijk
King's College Hospital, London
Royal Free Hospital, London
Maastricht University Medical Center+
San Raffaele Hospital, Milan
Freeman Hospital Newcastle
University Hospital Oslo
Derriford Hospital, Plymouth
University Hospital Southampton

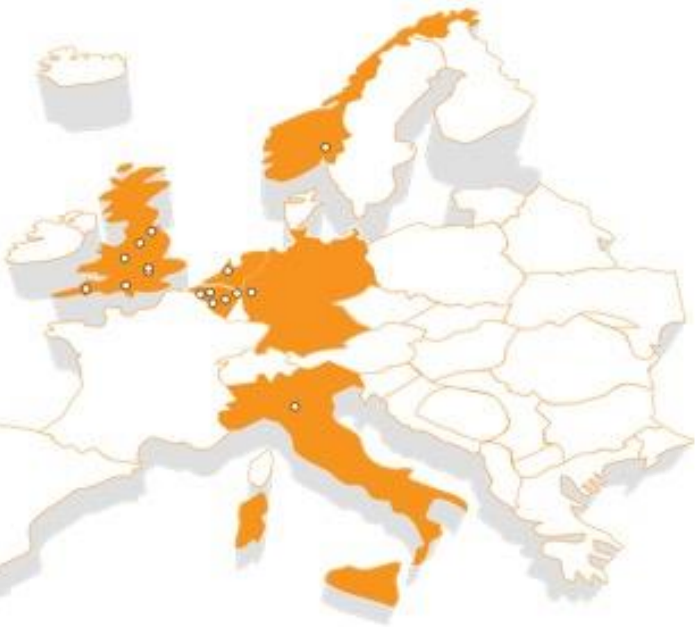

Appendix 8: Satava classification adapted for liver surgery

Grade	Definition of intraoperative incidents
Grade I ☐	Incidents managed without change of operative approach and without further consequences for the patient. It includes perforations of adherent or adjacent organs and minor change of intraoperative tactics and cases with blood loss over normal range (It corresponds to blood loss over 1000 ml in case of liver resection).
Grade II ☐	Incidents with consequences for patient. It includes cases requiring limited resection of intra-operatively injured organs or cases with blood loss which is appreciably over normal range (It corresponds to blood loss over 2000 ml in case of liver resection). In case of laparoscopic approach it also includes cases requiring conversion to open approach.*
Grade III ☐	Incident leading to significant consequences for patient.

* Conversion to hand-assisted laparoscopic resection is not scored as a Grade II intra-operative incident.

Appendix 9: Accordion Severity Grading System of Surgical Complications

Grade	Definition of postoperative complications
Grade I ☐	Requires only minor invasive procedures that can be done at the bedside, such as insertion of intravenous lines, urinary catheters, and nasogastric tubes, and drainage of wound infections. Physiotherapy and anti-emetics, antipyretics, analgesics, diuretics, electrolytes, and physiotherapy are permitted.
Grade II ☐	Requires pharmacologic treatment with drugs other than such allowed for minor complications, e.g. antibiotics. Blood transfusions and total parenteral nutrition are also included.
Grade III ☐	No general anaesthesia: requires management by an endoscopic, interventional procedure or reoperation without general anaesthesia.
Grade IV ☐	Requires management under general anaesthesia or single-organ failure.
Grade V ☐	Requires management under general anaesthesia and single organ failure or multisystem organ failure (> 2 organ systems).<br>Such complications would normally be managed in an increased acuity setting but in some cases patients with complications of lower severity might also be admitted to an ICU.
Grade VI ☐	Death.

Appendix 10: EQ5D

Appendix 10.1: EQ5D part 1

By placing a check-mark in one box in each group below, please indicate which statements best describe your own state of health today.

Mobility

I have no problems in walking about. ☐

I have some problems in walking about. ☐

I am confined to bed. ☐

Self-care

I have no problems with self-care. ☐

I have some problems washing or dressing myself. ☐

I am unable to wash or dress myself. ☐

Usual activities *(e.g. work, study, housework, family or leisure activities)*

have no problems with performing my usual activities. ☐

I have some problems with performing my usual activities. ☐

I am unable to perform my usual activities. ☐

Pain/discomfort

I have no pain or discomfort. ☐

I have moderate pain or discomfort. ☐

I have extreme pain or discomfort. ☐

Anxiety / Depression

I am not anxious or depressed. ☐

I am moderately anxious or depressed. ☐

I am extremely anxious or depressed. ☐

Appendix 10.2: EQ5D part 2

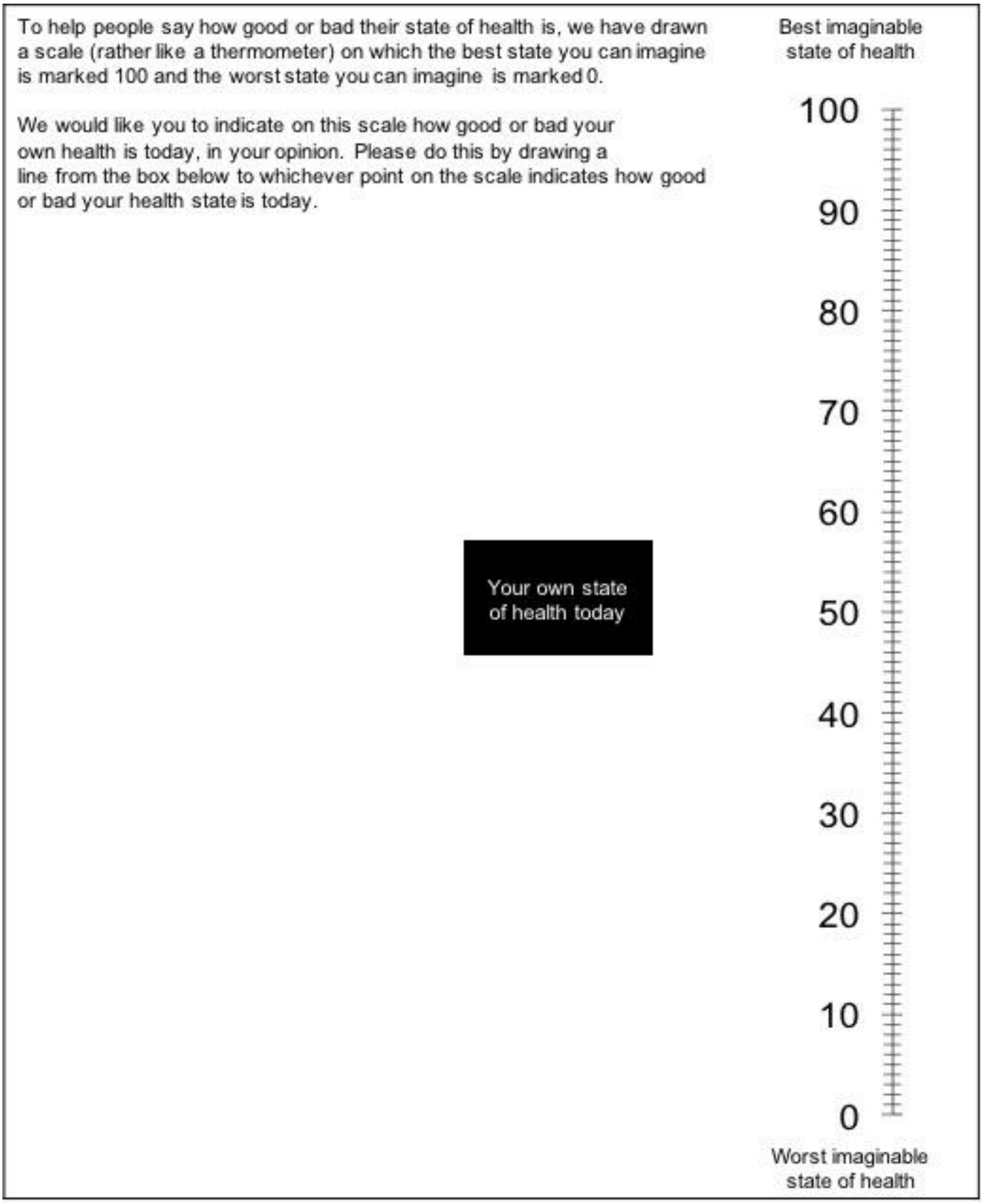

To help people say how good or bad their state of health is, we have drawn a scale (rather like a thermometer) on which the best state you can imagine is marked 100 and the worst state you can imagine is marked 0.

We would like you to indicate on this scale how good or bad your own health is today, in your opinion. Please do this by drawing a line from the box below to whichever point on the scale indicates how good or bad your health state is today.

Your own state of health today

Best imaginable state of health

100
90
80
70
60
50
40
30
20
10
0

Worst imaginable state of health

Appendix 11: EORTC QLQ-C30

Appendix 11.1: EORTC QLQ-C30 part 1

We are interested in some things about you and your health. Please answer all of the questions yourself by circling the number that best applies to you. There are no "right" or "wrong" answers. The information that you provide will remain strictly confidential.

Please fill in your initials:

Your birthdate (Day, Month, Year):

Today's date (Day, Month, Year):

		Not at all	A little	Quite a bit	Very much
1.	Do you have any trouble doing strenuous activities, like carrying a heavy shopping bag or a suitcase?	1	2	3	4
2.	Do you have any trouble taking a long walk?	1	2	3	4
3.	Do you have any trouble taking a short walk outside of the house?	1	2	3	4
4.	Do you need to stay in bed or a chair during the day?	1	2	3	4
5.	Do you need help with eating, dressing, washing yourself or using the toilet?	1	2	3	4

**During the past week:**		Not at all	A little	Quite a bit	Very much
6.	Were you limited in doing either your work or other daily activities?	1	2	3	4
7.	Were you limited in pursuing your hobbies or other leisure time activities?	1	2	3	4
8.	Were you short of breath?	1	2	3	4
9.	Have you had pain?	1	2	3	4
10.	Did you need to rest?	1	2	3	4
11.	Have you had trouble sleeping?	1	2	3	4
12.	Have you felt weak?	1	2	3	4
13.	Have you lacked appetite?	1	2	3	4
14.	Have you felt nauseated?	1	2	3	4

Please go on to the next page

Appendix 11.2: EORTC QLQ-C30 part 2

During the past week:		Not at all	A little	Quite a bit	Very much
15.	Have you vomited?	1	2	3	4
16.	Have you been constipated?	1	2	3	4
17.	Have you had diarrhoea?	1	2	3	4
18.	Were you tired?	1	2	3	4
19.	Did pain interfere with your daily activities?	1	2	3	4
20.	Have you had difficulty in concentrating on things, like reading a newspaper or watching television?	1	2	3	4
21.	Did you feel tense?	1	2	3	4
22.	Did you worry?	1	2	3	4
23.	Did you feel irritable?	1	2	3	4
24.	Did you feel depressed?	1	2	3	4
25.	Have you had difficulty remembering things?	1	2	3	4
26.	Has your physical condition or medical treatment interfered with your family life?	1	2	3	4
27.	Has your physical condition or medical treatment interfered with your social activities?	1	2	3	4
28.	Has your physical condition or medical treatment caused you financial difficulties?	1	2	3	4

For the following questions please circle the number between 1 and 7 that best applies to you.

29. How would you rate your overall health during the past week?

1	2	3	4	5	6	7
Very poor						Excellent

30. How would you rate your overall quality of life during the past week?

1	2	3	4	5	6	7
Very poor						Excellent

Appendix 12: EORTC QLQ-LM21

Patients sometimes report that they have the following symptoms or problems. Please indicate the extent to which you have experienced these symptoms or problems during the past week. Please answer by circling the number that best applies to you.

**During the past week:**		**Not at all**	**A little**	**Quite a bit**	**Very much**
31.	Have you had trouble with eating?	1	2	3	4
32.	Have you felt full up too quickly after beginning to eat?	1	2	3	4
33.	Have you worried about losing weight?	1	2	3	4
34.	Have you had problems with your sense of taste?	1	2	3	4
35.	Have you had a dry mouth?	1	2	3	4
36.	Have you had a sore mouth or tongue?	1	2	3	4
37.	Have you been less active than you would like to be?	1	2	3	4
38.	Have you had tingling hands or feet?	1	2	3	4
39.	Have you had pain in your stomach area?	1	2	3	4
40.	Have you had discomfort in your stomach area?	1	2	3	4
41.	Have your skin or eyes been yellow (jaundiced)?	1	2	3	4
42.	Have you had pain in your back?	1	2	3	4
43.	Have you felt slowed down?	1	2	3	4
44.	Have you felt lacking in energy?	1	2	3	4
45.	Have you had trouble having social contact with friends?	1	2	3	4
46.	Have you had trouble talking about your feelings to your family or friends?	1	2	3	4
47.	Have you felt stressed?	1	2	3	4
48.	Have you felt less able to enjoy yourself?	1	2	3	4
49.	Have you worried about your health in the future?	1	2	3	4
50.	Were you worried about your family in the future?	1	2	3	4
		1	2	3	4
**The past four weeks:**					
51.	Has the disease or treatment affected your sex life (for the worse)?	1	2	3	4

Appendix 13: Body Image Questionnaire

Dear Sir / Madam,

This questionnaire will help us to evaluate the effect of your illness and/or treatment on your body image and cosmesis. We kindly request you to mark the statement that suits you best.
3

1. Are you less satisfied with your body since the operation?
- ☐ No, not at all
- ☐ A little bit
- ☐ Quite a bit
- ☐ Yes, extremely

2. Do you think the operation has damaged your body?
- ☐ No, not at all
- ☐ A little bit
- ☐ Quite a bit
- ☐ Yes, extremely

3. Do you feel less attractive as a result of your disease or treatment?
- ☐ No, not at all
- ☐ A little bit
- ☐ Quite a bit
- ☐ Yes, extremely

4. Do you feel less feminine/masculine as a result of your disease or treatment?
- ☐ No, not at all
- ☐ A little bit
- ☐ Quite a bit
- ☐ Yes, extremely

5. Is it difficult to look at yourself naked?
- ☐ No, not at all
- ☐ A little bit
- ☐ Quite a bit
- ☐ Yes, extremely

6. On a scale from 1 to 7, how satisfied are you with your (incisional) scar?

1 = very unsatisfied	2	3	4 = not satisfied / not unsatisfied	5	6	7 = very satisfied

7. On a scale from 1 to 7, how would you describe your (incisional) scar?

1 = very unsatisfied	2	3	4 = not satisfied / not unsatisfied	5	6	7 = very satisfied

8. Could you score your own incisional scar on a scale from 1 to 10? ☐

Appendix 14: Cost questionnaire

Appendix 14.1: Cost questionnaire part 1

General Practitioner

1a. **Did you consult with a general practitioner at any time during the *last three months* in relation to your liver operation?**

☐ NO, continue to question 2a.
☐ YES ______ time(s)

1b. **What is the distance to your general practitioner?**

☐ < 5 km away
☐ 5 – 10 km away
☐ 10 – 20 km away
☐ > 21 km away

1c. **What type of transport did you use?**

By foot / bicycle ______ time(s)
Car ______ time(s)
Public transport ______ time(s)
Taxi ______ time(s)

Emergency Department

2a. **Did you visit an emergency department at any time during the *last three months* in relation to your liver operation?**

☐ NO, continue to question 3a.
☐ YES ______ time(s)

2b. **What type of transport did you use?**

By foot / bicycle ______ time(s)
Car ______ time(s)
Public transport ______ time(s)
Taxi ______ time(s)

2c. **Which hospital did you visit?**

☐ University hospital: ____________________
☐ General hospital: ____________________

Medical specialist(s)

3a. **Did you consult a medical specialist at the outpatient clinic at any time during the *last three months* in relation to your liver operation?**

☐ NO, continue to question 5a.
☐ YES ______ time(s)

3b. **What type of transport did you use?**

By foot / bicycle ______ time(s)
Car ______ time(s)
Public transport ______ time(s)
Taxi ______ time(s)

3c. **Which hospital did you visit?**

☐ University hospital: ____________________
☐ General hospital: ____________________

3d. **What type of medical specialist did you consult? (Surgeon, internist, oncologist)**

1. ______ time(s)
2. ______ time(s)
3. ______ time(s)
4. ______ time(s)

Appendix 14.2: Cost questionnaire part 2

Hospital admission(s)

4a. **Were you admitted to a hospital at any time during the *last three months* in relation to your liver operation?**
☐ NO, continue to question 5.
☐ YES ______ time(s)

4b. **What type of transport did you use?**
By foot / bicycle ______ time(s)
Car ______ time(s)
Public transport ______ time(s)
Taxi ______ time(s)

4c. **Which hospital did you visit?**
☐ University hospital: ______________________________
☐ General hospital: ______________________________

4d. **How many days were you admitted in hospital?**
Total ______ days

Household / homecare support

5. **Did other people take over and perform your usual household tasks or provide home care in the past three months because of your health problems?**
☐ NO, continue to question 6.
☐ YES. ______ time(s)

Family members/friends: ______ hours per week
Home care: ______ hours per week Pounds per hour ______________
Other paid care ______ hours per week Pounds per hour ______________

Paramedic consultation

6. **Did you consult with a paramedical worker at any time during the *past three months*? (Examples *of paramedical workers area: physical therapist, speech therapist, Caesar therapist, ergotherapist, manual therapist, Mensendieck therapist)***
☐ NO, continue to question 7.
☐ YES ______ time(s)

Alternative healers

7. **Did you consult with an alternative healer at any time *during the three months*? (Examples *of alternative healers include homeopaths, acupuncturists, natural healers, iriscopists, holistic therapists, paragnostics, Reiki therapists)***
☐ NO, continue to question 8.
☐ YES ______ time(s)

Appendix 14.3: Cost questionnaire part 3

Medication

8a. **Have you taken medication of any kind over the *past three months*?**
(Not including medication prescribed during admission to hospital, or the contraceptive pill)

☐ NO, continue to question 9.
☐ YES ______ time(s)

8b. **What type of medication did you use?**

1.	Dose per day: ______________	Number of days:	______
2.	Dose per day: ______________	Number of days:	______
3.	Dose per day: ______________	Number of days:	______
4.	Dose per day: ______________	Number of days:	______
5.	Dose per day: ______________	Number of days:	______
6.	Dose per day: ______________	Number of days:	______
7.	Dose per day: ______________	Number of days:	______

Work

9a. **Do you currently have a paid profession?**
☐ NO, you have completed this questionnaire.
☐ YES.

9b. **How many hours does your contract specify?**
______ hours per week

9c. **Over how many days are these hours distributed?**
______ days

9d. **What is your occupation?**

____

9e. **What is your own net income from paid work ? (*This refers to the amount you actually receive. We are interested only in your income, i.e. exclusive of - if present - your partner's income)***
______ Pounds per week, or
______ Pounds per 4 weeks, or
______ Pounds per month, or
______ Pounds per year
☐ Don't know/don't wish to reveal

9f. **Did health problems oblige you to be off work at any time in the *past three months?***
☐ NO, you have completed this questionnaire.
☐ YES ______ working days
