# Supplementary material for: Health-related quality of life in patients undergoing laparoscopic versus open hemihepatectomy: a secondary analysis of the ORANGE II PLUS randomised controlled, phase 3, superiority trial
Source: Lancet Reg Health Eur. 2025 May 19;54:101311. doi: 10.1016/j.lanepe.2025.101311 (PMC12148447; doi:10.1016/j.lanepe.2025.101311)
Supplement: ORANGE II PLUS Collaborators list of Study Group [file mmc3.docx]

| First name | Surname |
| --- | --- |
| Ronald M. | Van Dam |
| Luca A. | Aldrighetti |
| Mohammed | Abu Hilal |
| Roberto I. | Troisi |
| Robert P. | Sutcliffe |
| Marc G. | Besselink |
| Somaiah | Aroori |
| Krishna V. | Menon |
| Bjørn | Edwin |
| Mathieu | D’Hondt |
| Valerio | Lucidi |
| Tom F. | Ulmer |
| Rafael | Diaz-Nieto |
| Zahir | Soonawalla |
| Steve | White |
| Gregory | Sergeant |
| Robert S. | Fichtinger |
| Bram | Olij |
| Francesca | Ratti |
| Christoph | Kuemmerli |
| Vincenzo | Scuderi |
| Frederik | Berrevoet |
| Aude | Vanlander |
| Ravi | Marudanayagam |
| Pieter J. | Tanis |
| Maxime J.L. | Dewulf |
| Zina B. | Eminton |
| Ulf P. | Neumann |
| Lloyd | Brandts |
| Siân A. | Pugh |
| Åsmund A. | Fretland |
| Merel L. | Kimman |
| John N. | Primrose |
| Remon | Korenblik |
| Michelle | Lintforth |
| Burak | Gorçek |
| Penelope | Rogers |
| Viviane | Van Laethem |
| Betsy | Van Loo |
| Kathleen | Segers |
| Celine | Demeyere |
| Ane | Zamalloa |
| Cornelis | Dejong |
| Davit | Aghayan |
| Katherine | Gordon-Quayle |
| Tracy | Ward |
| Jess | Boxal |
| Beth | Wedge |
