# Supplementary material for: Health-related quality of life in patients undergoing laparoscopic versus open hemihepatectomy: a secondary analysis of the ORANGE II PLUS randomised controlled, phase 3, superiority trial
Source: Lancet Reg Health Eur. 2025 May 19;54:101311. doi: 10.1016/j.lanepe.2025.101311 (PMC12148447; doi:10.1016/j.lanepe.2025.101311)
Supplement: ORANGE II PLUS HRQoL Supplementary Material with caption [file mmc4.docx]

**Supplementary Material**

**Table of Content**

[Supplementary Documents 2](#_Toc188618355)

[Supplementary document 1: Details of statistical analysis 2](#_Toc188618356)

[Supplementary document 2: CONSORT checklist abstract 3](#_Toc188618357)

[Supplementary document 3: CONSORT 2010 checklist with the CONSORT Harms 2022 elaboration of information to include when reporting a randomised trial (including 2013 PRO and 2017 NPT extensions) 4](#_Toc188618358)

[Supplementary document 4: Delta2 recommended reporting items for the sample size calculation of a randomised controlled trial with a superiority question 7](#_Toc188618359)

[Supplementary Tables 8](#_Toc188618360)

[Supplementary Table 1: univariate effect difference over the period of discharge to 3 months and 12 months after either laparoscopic or open hemihepatectomy in the mITT population 8](#_Toc188618361)

[Supplementary Table 2: Multivariable adjusted differences of global health scale per timepoint of patients undergoing laparoscopic or open hemihepatectomy in the mITT population 10](#_Toc188618362)

[Supplementary Table 3: Subgroup analysis. Multivariable adjusted differences of body image and cosmesis of patients undergoing laparoscopic or open hemihepatectomy in the mITT population, differences per sexes. 11](#_Toc188618363)

[Supplementary Table 4: subgroup analysis. Cumulative HRQoL differences over the period of discharge to 12 months after either laparoscopic or open hemihepatectomy in patients with malignant indications in the mITT population 12](#_Toc188618364)

[Supplementary Table 5: Subgroup analysis. Multivariable adjusted differences of global health scale per country of patients undergoing laparoscopic or open hemihepatectomy in the mITT population. 13](#_Toc188618365)

[Supplementary Table 6: Subgroup demographics. Recurrence and adjuvant systemic therapy at 12 months of patients with malignant disease undergoing laparoscopic or open hemihepatectomy in the mITT population. 14](#_Toc188618366)

[Supplementary Table 7: 90-day mortality causes 15](#_Toc188618367)

[Supplementary Table 8: Median HRQoL outcomes at baseline for patients undergoing either laparoscopic or open hemihepatectomy with multiple imputation in the mITT population 17](#_Toc188618368)

[Supplementary Table 9: Distribution of patients per centre. 18](#_Toc188618369)

[Supplementary Figures 19](#_Toc188618370)

[Supplementary Figure 1: EORTC-QLQ-C30 not selected symptom scales over the period of baseline to 12 months after after either laparoscopic or open hemihepatectomy in the mITT population 19](#_Toc188618371)

[Supplementary Figure 2: EORTC-QLQ-LMC21 not selected symptom scales over the period of baseline to 12 months after after either laparoscopic or open hemihepatectomy in the mITT population 20](#_Toc188618372)

[Supplementary List of collaborators 21](#_Toc188618373)

# **Supplementary Documents**

## Supplementary document 1: Details of statistical analysis

| Sensitivity analysis through multiple imputation |
| --- |
| The multiple imputation model is used as sensitivity analysis and not as primary analysis because a linear mixed model is applied, which already provides an unbiased estimate of the treatment effect that would have been observed if missing data were dependent on known and observed factors.^1^ Thus, it is recommended to base conclusions on the original dataset.^2,3^ The imputed values were based on age, sex, hemihepatectomy side, tumour type, centre, scores of the same patient on the same item at different time points, and scores of the same patient of other items at the same time point. Imputed values of questionnaires of patients after they had died or were lost-to-follow-up were excluded from the analyses, as well as all questionnaires of patients who only filled in the baseline questionnaire, due to the unreliability on which imputation of these data is based.^3^  To retain the same population for analysis as the unimputed analysis, all patients with 1 questionnaire (n=28) filled out were not included as well as imputed questionnaires of patients after death (n=28) or lost-to-follow-up (n=7). Hereafter, the linear mixed model was performed again on the imputed dataset. |
| Clinical relevance |
| In large cohorts, small deviations in patient-reported outcome measures can achieve statistical significance which might not be clinically meaningful. Therefore, we only considered differences as clinically relevant if they met the criteria specified per individual scale of the EORTC QLQ-C30 as defined by Cocks et al.^4^ For example a difference in GHS between treatment arms of -5≤ or ≥5 is deemed clinically relevant, but for physical functioning a difference of -5≤ or ≥2 is deemed clinically relevant. Meaning that a difference of 3 points would not be clinically relevant for GHS, but would be for physical functioning. To our knowledge, thresholds for clinical relevance do not exist for the symptoms in the EORTC-QLQ-LMC21 questionnaire, therefore, in coherence with the average of the references by Cocks et al, we defined a difference of 4 points between arms as a small clinically relevant difference and a difference of 9 points as a large clinically relevant difference. |
| Randomisation |
| the local investigator, c.q. study nurses or designated physicians, used online randomisation software (TENALEA, Version 3.0) to allocate patients, 1:1, to receive either laparoscopic or open hemihepatectomy. A minimization scheme was used to balance patient allocation between groups, with stratification by centre and by the side of the hemihepatectomy, right or left. In the case of an imbalance of 2 patients, the probability of being assigned to the underrepresented group was 90%. |
| Database and data handling |
| Pseudo-anonymised data were collected by surgeons, trial nurses and ward personnel on paper case record forms and manually digitised into data capturing software (OpenClinica, Community Version 3·14), stored in a secured Oracle database for a maximum period of 15 years. Each local investigator safeguarded the identifying key for their patients on a local institutional secured server. Data checks and data cleaning were performed with automated scripts in SPSS Statistics software (IBM, Windows Version 27·0 ·1 ·0). Analyses were performed using SPSS Statistics software (IBM, Windows Version 27·0 ·1 ·0) and visualised with R software (R project for Statistical Computing, Windows Version 4·1 ·0). |
| *Supplementary Document 1: Details of statistical analysis.* |

## Supplementary document 2: CONSORT checklist abstract

| Recommended reporting items | Description | Check |
| --- | --- | --- |
| Title | Identification of the study as randomised | X |
| Authors (for conference abstracts) | Contact details for the corresponding author | n.a. |
| Trial design | Description of the trial design (eg, parallel, cluster, non-inferiority) | X |
|  |  |  |
| Methods |  |  |
| Participants | Eligibility criteria for participants and the settings where the data were collected | X |
| Interventions | Interventions intended for each group | X |
| Objective | Specific objective or hypothesis | X |
| Outcome | Clearly defined primary outcome for this report | n.a. |
| Randomisation | How participants were allocated to interventions | X |
| Blinding (masking) | Whether or not participants, care givers, and those assessing the outcomes were blinded to group assignment | n.a. |
| Results |  |  |
| Numbers randomised | Number of participants randomised to each group | X |
| Recruitment | Trial status | X |
| Numbers analysed | Number of participants analysed in each group | X |
| Outcome | For the primary outcome, a result for each group and the estimated effect size and its precision | n.a. |
| Harms | Important adverse events or side-effects | n.a. |
| Conclusions | General interpretation of the results | X |
| Trial registration | Registration number and name of trial register | X |
| Funding | Source of funding | X |

## Supplementary document 3: CONSORT 2010 checklist with the CONSORT Harms 2022 elaboration of information to include when reporting a randomised trial (including 2013 PRO and 2017 NPT extensions)

| **Section/Topic** | **Item** | **Checklist item** | **Reported on page no.** |
| --- | --- | --- | --- |
| **Title and abstract** | | | |
|  | 1a | Identification as a randomized trial in the title | 1 |
|  | 1b | Structured summary of trial design, methods, results of outcomes of benefits and harms, and conclusions (for specific guidance see CONSORT for abstracts) | 3 |
|  | 1b PRO | The PRO should be identified in the abstract as a primary or secondary outcome | 3 |
|  | 1b NPT | Refer to CONSORT extension for abstracts for NPT trials | 7 |
| **Introduction** | | | |
| Background and objectives | 2a | Scientific background and explanation of rationale | 5 |
|  | 2a PRO | Including background and rationale for PRO assessment | 5 |
|  | 2b | Specific objectives or hypotheses for outcomes of benefits and harms | 5 |
|  | 2b PRO | The PRO hypothesis should be stated and relevant domains identified | 5, 7 |
| **Methods** | | | |
| Trial design | 3a | Description of trial design (such as parallel, factorial) including allocation ratio | 6-7 |
|  | 3a NPT | When applicable, how care providers were allocated to each trial group | n.a. |
|  | 3b | Important changes to methods after trial commencement (such as eligibility criteria), with reasons | 7 |
| Participants | 4a | Eligibility criteria for participants | 6 |
|  | 4a PRO | Not PRO-specific, unless the PROs were used in eligibility or stratification criteria | n.a. |
|  | 4a NPT | When applicable, eligibility criteria for centres and for care providers | 6 |
|  | 4b | Settings and locations where the data were collected | 6 |
| Interventions | 5 | The interventions for each group with sufficient details to allow replication, including how and when they were actually administered | 6 |
|  | 5 NPT | Precise details of both the experimental treatment and comparator | 6 |
|  | 5a NPT | Description of the different components of the intervention and, when applicable, description of the procedure for tailoring the interventions to individual participants. | 6 |
|  | 5b NPT | Details of whether and how the interventions were standardised. | 6 |
|  | 5c NPT | Details of whether and how adherence of care providers to the protocol was assessed or enhanced. | n.a. |
|  | 5d NPT | Details of whether and how adherence of participants to interventions was assessed or enhanced. | 9 |
| Outcomes | 6a | Completely defined prespecified primary and secondary outcomes, for both benefits and harms, including how and when they were assessed | 6-7 |
|  | 6a PRO | Evidence of PRO instrument validity and reliability should be provided or cited if available including the person completing the PRO and methods of data collection (paper, telephone, electronic, other) | 6-7 |
|  | 6b | Any changes to trial outcomes after the trial commenced, with reasons | 7 |
|  | 6c | Describe if and how non-prespecified outcomes of benefits and harms were identified, including any selection criteria, if applicable | 41-42 |
| Sample size | 7a | How sample size was determined | 7 |
|  | 7a PRO | Not required for PRO unless it is a primary study outcome | 7 |
|  | 7a NPT | When applicable, details of when and how the clustering by care providers was addressed | 6-7, 27 |
|  | 7b | When applicable, explanation of any interim analyses and stopping guidelines | 7 |
| Randomization | 8a | Method used to generate the random allocation sequence | 6-7, 27 |
| Sequence generation | 8b | Type of randomization; details of any restriction (such as blocking and block size) | 6, 27 |
| Allocation concealment mechanism | 9 | Mechanism used to implement the random allocation sequence (such as sequentially numbered containers), describing any steps taken to conceal the sequence until interventions were assigned | 6 ,27 |
| Implementation | 10 | Who generated the random allocation sequence, who enrolled participants, and who assigned participants to interventions | 6, 27 |
| Blinding | 11a | If done, who was blinded after assignment to interventions (for example, participants, care providers, those assessing outcomes of benefits and harms) and how | 6 |
|  | 11a NPT | If done, who was blinded after assignment to interventions (e.g., participants, care providers, those administering co-interventions, those assessing outcomes) and how | 6 |
|  | 11b | If relevant, description of the similarity of interventions | n.a. |
|  | 11b NPT | If blinded, methods of blinding and description of the similarity of interventions | 6 |
|  | 11c | If blinding was not possible, description of any attempts to limit bias | 6 |
| Statistical methods | 12a | Statistical methods used to compare groups for primary and secondary outcomes of both benefits and harms | 6-8 |
|  | 12a NPT | When applicable, details of whether and how the clustering by care providers or centres was addressed | 7-8 |
|  | 12a PRO | Statistical approaches for dealing with missing data are explicitly stated | 7-8 |
|  | 12b | Methods for additional analyses, such as subgroup analyses and adjusted analyses | 8 |
| **Results** | | | |
| Participant flow (a diagram is strongly recommended) | 13a | For each group, the numbers of participants who were randomly assigned, received intended treatment, and were analysed for outcomes of benefits and harms | 9 |
|  | 13a PRO | The number of PRO outcome data at baseline and at subsequent time points should be made transparent | 9, 24 |
|  | 13a NPT | The number of care providers or centres performing the interventions in each group and the number of patients treated by each care provider or in each centre | 44 |
|  | 13b | For each group, losses and exclusions after randomization, together with reasons | 9, 24 |
|  | 13c NPT | For each group, the delay between randomisation and the initiation of the intervention | n.a. |
|  | 13 NPT | Details of the experimental treatment and comparator as they were implemented | 6 |
| Recruitment | 14a | Dates defining the periods of recruitment and follow-up for outcomes of benefits and harms | 9 |
|  | 14b | Why the trial ended or was stopped | n.a. |
| Baseline data | 15 | A table showing baseline demographic and clinical characteristics for each group | 18, 19 |
|  | 15 PRO | Including baseline PRO data when collected | 19 |
|  | 15 NPT | When applicable, a description of care providers (case volume, qualification, expertise, etc.) and centres (volume) in each group | 44 |
| Numbers analysed | 16 | For each group, number of participants (denominator) included in each analysis of outcomes of benefits and harms and whether the analysis was by original assigned groups and if any exclusions were made | 9, 24, 41-42 |
|  | 16 PRO | Required for PRO results | 9, 24, 41-42 |
| Outcomes and estimation | 17a | For each primary and secondary outcome of benefits and harms, results for each group, and the estimated effect size and its precision (such as 95% confidence interval) | 9, 10, 21-23 |
|  | 17a2 | For outcomes omitted from the trial report (benefits and harms), provide rationale for not reporting and indicate where the data on omitted outcomes can be accessed | 6-7 |
|  | 17a PRO | For multidimensional PRO results from each domain and time point | 9, 10, 21-23, 25, 26, 45, 46 |
|  | 17b | Presentation of both absolute and relative effect sizes is recommended, for outcomes of benefits and harms | 41, 42 |
|  | 17c | Report zero events if no harms were observed | 41, 42 |
| Ancillary analyses | 18 | Results of any other analyses performed for outcomes of benefits and harms, including subgroup analyses and adjusted analyses, distinguishing prespecified from exploratory | 10 |
|  | 18 PRO | Including PRO results, where relevant | 9, 10 |
| Harms | 19 | All important harms or unintended effects in each group (for specific guidance see CONSORT for harms) | 41, 42 |
| **Discussion** | | | |
| Limitations | 20 | Trial limitations, addressing sources of potential bias related to the approach to collecting or reporting data on harms, imprecision, and, if relevant, multiplicity or selection of analyses | 12 |
|  | 20 NPT | In addition, take into account the choice of the comparator, lack of or partial blinding, and unequal expertise of care providers or centres in each group | 12 |
| Generalizability | 21 | Generalisability (external validity, applicability) of the trial findings | 11, 12 |
|  | 20/21 PRO | PRO-specific limitations and implications generalizability and clinical practice | 12 |
|  | 21 NPT | Generalisability (external validity, applicability) of the trial findings according to the intervention, comparators, patients, and care providers and centres involved in the trial | 11, 12 |
| Interpretation | 22 | Interpretation consistent with results, balancing benefits and harms, and considering other relevant evidence | 10, 11 |
|  | 22 PRO | PRO data should be interpreted in relation to clinical outcomes including survival data, when relevant | 10, 11 |
| **Other information** | | | |
| Registration | 23 | Registration number and name of trial registry | 6, 16 |
| Protocol | 24 | Where the full trial protocol and other relevant documents can be accessed, including additional data on harms | 16, 41, 42 |
| Funding | 25 | Sources of funding and other support (such as supply of drugs), role of funders | 16 |
| ***Supplementary Document 2:* CONSORT 2010 checklist with the CONSORT Harms 2022 elaboration of information to include when reporting a randomised trial (including 2013 PRO and 2017 NPT extensions)** | | | |

## Supplementary document 4: Delta2 recommended reporting items for the sample size calculation of a randomised controlled trial with a superiority question

| Recommended reporting items | Page where item is reported |
| --- | --- |
| Core Items |  |
| (1) Primary outcome (and any other outcome on which the calculation is based) | 6 |
| If a primary outcome is not used as the basis for the sample size calculation, state why | 6, 7 |
| (2) Statistical significance level and power | 7 |
| (3) Express the target difference according to outcome type | 7 |
| (a) Binary—state the target difference as an absolute or relative effect (or both), along with the intervention and control group proportions· If both an absolute and a relative difference are provided, clarify if either takes primacy in terms of the sample size calculation | n.a. |
| (b) Continuous—state the target mean difference on the natural scale, common standard deviation, and standardised effect size (mean difference divided by the standard deviation) | 7 |
| (c) Time-to-event—state the target difference as an absolute or relative difference (or both); provide the control group event proportion, planned length of follow-up, intervention and control group survival distributions, and accrual time (if assumptions regarding them are made)· If both an absolute and relative difference are provided for a particular time point, clarify if either takes primacy in terms of the sample size calculation | n.a. |
| (4) Allocation ratio | 6, 7 |
| If an unequal ratio is used, the reason for this should be stated | n.a. |
| (5) Sample size based on the assumptions as per above | 7 |
| (a) Reference the formula/sample size calculation approach, if standard binary, continuous, or survival outcome formulas are not used· For a time-to-event outcome, the number of events required should be stated | 7 |
| (b) If any adjustments (eg, allowance for loss to follow-up, multiple testing) that alter the required sample size are incorporated, they should also be specified, referenced, and justified along with the final sample size | 7, 8 |
| (c) For alternative designs, additional input should be stated and justified· For example, for a cluster randomised controlled trial (or an individually randomised controlled trial with clustering), state the average cluster size and intracluster correlation coefficient(s)· Variability in cluster size should be considered and, if necessary, the coefficient of variation should be incorporated into the sample size calculation· Justification for the values chosen should be given | n.a. |
| (d) Provide details of any assessment of the sensitivity of the sample size to the inputs used | 7, 8 |
| Additional items for grant application and trial protocol | n.a. |
| (6) Underlying basis used for specifying the target difference (an important or realistic difference) | 7 |
| (7) Explain the choice of target difference—specify and reference any formal method used or relevant previous research | 7 |
| Additional item for trial results paper | 7, 8 |
| (8) Reference the trial protocol | 7 |

# Supplementary Tables

## Supplementary Table 1: univariate effect difference over the period of discharge to 3 months and 12 months after either laparoscopic or open hemihepatectomy in the mITT population

| **HRQoL outcome** | **Univariate difference to 3 months**  **(mean [95% CI])** | **Univariate difference to 12 months**  **(mean [95% CI])** | **Clinical relevance range^$^** |
| --- | --- | --- | --- |
| **QLQ-C30 – global health status** |  |  |  |
| LH | 4·49 (1·00 to 8·00) | 2·62 (-0·40 to 5·63) | -5≤ or ≥5 |
| OH | Reference | Reference |  |
| **QLQ-C30 - functional scales** |  |  |  |
| **Physical functioning** |  |  |  |
| LH | 4·32 (1·09 to 7·55) | 3·76 (1·02 to 6·51) | -5≤ or ≥2 |
| OH | Reference | Reference |  |
| **Role functioning** |  |  |  |
| LH | 4·73 (0·16 to 9·30) | 4·38 (0·66 to 8·10) | -7≤ or ≥6 |
| OH | Reference | Reference |  |
| **Emotional functioning** |  |  |  |
| LH | 2·26 (-1·38 to 5·91) | 0·88 (-2·50 to 4·26) | -3≤ or ≥6 |
| OH | Reference | Reference |  |
| **Cognitive functioning** |  |  |  |
| LH | 3·64 (0·25 to 7·03) | 2·90 (-1·33 to 5·94) | -1≤ or ≥3 |
| OH | Reference | Reference |  |
| **Social functioning** |  |  |  |
| LH | 6·27 (1·51 to 11·03) | 3·90 (-0·25 to 8·06) | -6≤ or ≥3 |
| OH | Reference | Reference |  |
| **QLQ-C30 - symptom scales** |  |  |  |
| **Fatigue** |  |  |  |
| LH | -4·91 (-8·98 to -0·84) | -3·02 (-6·76 to 0·71) | -5≤ or ≥4 |
| OH | Reference | Reference |  |
| **Pain** |  |  |  |
| LH | -6·28 (-10·00 to -2·55) | -4·94 (-8·10 to -1·78) | -3≤ or ≥5 |
| OH | Reference | Reference |  |
| **Appetite loss** |  |  |  |
| LH | -6·53 (-11·05 to -2·01) | -3·99 (-7·61 to -0·37) | -2≤ or ≥7 |
| OH | Reference | Reference |  |
| **QLQ-LMC21 - symptom scales** |  |  |  |
| **Nutritional problems** |  |  |  |
| LH | -3·95 (-7·81 to -0·10) | -2·66 (-5·70 to 0·38) | Na |
| OH | Reference | Reference |  |
| Peripheral neuropathy |  |  |  |
| LH | 1·34 (-2·36 to 5·04) | 0·98 (-2·51 to 4·47) | Na |
| OH | Reference | Reference |  |
| **Body image & Cosmesis** |  |  |  |
| **Body Image^†^** |  |  |  |
| LH | -1·00 (-1·58 to -0·41) | -0·82 (-1·36 to -0·27) | Na |
| OH | Reference | Reference |  |
| **Cosmesis^¥^** |  |  |  |
| LH | 2·21 (1·35 to 3·07) | 2·11 (1·30 to 2·93) | Na |
| OH | Reference | Reference |  |

## Supplementary Table 2: Multivariable adjusted differences of global health scale per timepoint of patients undergoing laparoscopic or open hemihepatectomy in the mITT population

| **Global health scale - timepoint** | **Adjusted difference^£^**  **(mean [95% CI])** |
| --- | --- |
| **Discharge** |  |
| LH | 4·17 (-0·39 to 8·67) |
| OH | Reference |
| **10 days** |  |
| LH | 6·72 (2·86 to 10·59) |
| OH | Reference |
| **3 months** |  |
| LH | 2·53 (-1·60 to 6·66) |
| OH | Reference |
| **6 months** |  |
| LH | 1·17 (-3·10 to 5·43) |
| OH | Reference |
| **12 months** |  |
| LH | -0·45 (-5·17 to 4·28) |
| OH | Reference |
| ^£^ Results adjusted for sex, age, hemihepatectomy side, benign/malignant tumour type, treatment centre, and baseline differences. In all analyses, the open group is used as reference group. | |
| ***Supplementary Table 2:* Multivariable adjusted differences of global health scale per timepoint of patients undergoing laparoscopic or open hemihepatectomy in the mITT population.** | |

## Supplementary Table 3: Subgroup analysis. Multivariable adjusted differences of body image and cosmesis of patients undergoing laparoscopic or open hemihepatectomy in the mITT population, differences per sexes.

|  | **Adjusted difference^£^**  **(mean [95% CI])** | **Adjusted difference^£^**  **(mean [95% CI])** |
| --- | --- | --- |
| **Body image - female** |  |  |
| LH (n = 67) | -1·70 (-2·21 to -1·19) | -1·52 (-2·04 to -1·00) |
| OH (n = 70) | Reference | Reference |
| **Body image - male** | -0·06 (0·39 to 0·27) | -0·15 (-0·47 to 0·17) |
| LH (n = 99) |  |  |
| OH (n = 96) | Reference | Reference |
| **Cosmesis - female** |  |  |
| LH (n = 67) | 2·83 (2·03 to 3·64) | 2·87 (2·08 to 3·65) |
| OH (n = 70) | Reference | Reference |
| **Cosmesis - male** |  |  |
| LH (n = 96) | 1·29 (0·69 to 1·90) | 1·36 (0·78 to 1·94) |
| OH (n = 96) | Reference | Reference |
| ^£^ Results adjusted for sex, age, hemihepatectomy side, benign/malignant tumour type, treatment centre, and baseline differences. In all analyses, the open group is used as reference group. | | |
| ***Supplementary Table 3:* Subgroup analysis. Multivariable adjusted differences of body image and cosmesis of patients undergoing laparoscopic or open hemihepatectomy in the mITT population, differences per sexes.** | | |

## Supplementary Table 4: subgroup analysis. Cumulative HRQoL differences over the period of discharge to 12 months after either laparoscopic or open hemihepatectomy in patients with malignant indications in the mITT population

| **HRQoL outcome** | **Univariate difference**  **(mean [95% CI])** | **Adjusted difference^£^**  **(mean [95% CI])** |
| --- | --- | --- |
| **QLQ-C30 – global health status** |  |  |
| LH | 3·23 (-0·14 to 6·60) | 4·04 (0·98 to 7·09) |
| OH | Reference | Reference |
| **QLQ-C30 - functional scales** |  |  |
| **Physical functioning** |  |  |
| LH | 3·35 (0·34 to 6·36) | 3·82 (0·84 to 6·81) |
| OH | Reference | Reference |
| **Role functioning** |  |  |
| LH | 4·82 (0·71 to 8·94) | 5·00 (1·27 to 8·73) |
| OH | Reference | Reference |
| **Emotional functioning** |  |  |
| LH | 0·57 (-3·12 to 4·27) | 1·11 (-2·56 to 4·81) |
| OH | Reference | Reference |
| **Cognitive functioning** |  |  |
| LH | 3·07 (0·27 to 6·41) | 2·87 (0·42 to 4·93) |
| OH | Reference | Reference |
| **Social functioning** |  |  |
| LH | 3·64 (-1·03 to 8·30) | 4·37 (0·02 to 8·72) |
| OH | Reference | Reference |
| **QLQ-C30 - symptom scales** |  |  |
| **Fatigue** |  |  |
| LH | -3·10 (-7·15 to 0·95) | -3·0 (-6·79 to 0·79) |
| OH | Reference | Reference |
| **Pain** |  |  |
| LH | -4·28 (-7·61 to -0·94) | -4·74 (-8·04 to -1·44) |
| OH | Reference | Reference |
| **Appetite loss** |  |  |
| LH | -3·17 (-7·11 to 0·77) | -4·05 (-7·96 to -0·13) |
| OH | Reference | Reference |
| **QLQ-LMC21 - symptom scales** |  |  |
| **Nutritional problems** |  |  |
| LH | -2·15 (-5·48 to 1·18) | -2·76 (-6·06 to 0·55) |
| OH | Reference | Reference |
| **Peripheral neuropathy** |  |  |
| LH | 0·55 (-3·33 to 4·42) | 0·71 (-3·05 to 4·48) |
| OH | Reference | Reference |
| **Body image & Cosmesis** |  |  |
| **Body Image^†^** |  |  |
| LH | -0·85 (-1·46 to -0·24) | -0·93 (-1·52 to -0·34) |
| OH | Reference | Reference |
| **Cosmesis^¥^** |  |  |
| LH | 2·20 (1·32 to 3·08) | 2·38 (1·60 to 3·16) |
| OH | Reference | Reference |
| ^£^ Results adjusted for sex, age, hemihepatectomy side, benign/malignant tumour type, treatment centre, and baseline differences. In all analyses, the open group is used as reference group. | | |
| ***Supplementary Table 4:* Subgroup analysis. Cumulative outcome differences of health-related quality of life over the period of discharge to 12 months of patients with malignant disease undergoing laparoscopic or open hemihepatectomy in the mITT population.** | | |

## Supplementary Table 5: Subgroup analysis. Multivariable adjusted differences of global health scale per country of patients undergoing laparoscopic or open hemihepatectomy in the mITT population.

| **Global health scale – country^*^** | **Adjusted difference^£^**  **(mean [95% CI])** |
| --- | --- |
| **The Netherlands (n = 40)** |  |
| LH | 5·93 (-0·21 to 12·07) |
| OH | Reference |
| **Italy (n = 85)** |  |
| LH | 2·34 (0·38 to 4·31) |
| OH | Reference |
| **Belgium (n = 72)** |  |
| LH | 3·99 (0·17 to 7·81) |
| OH | Reference |
| **United Kingdom (n = 115)** |  |
| LH | 3·75 (-0·84 to 8·34) |
| OH | Reference |
| ^£^ Results adjusted for sex, age, hemihepatectomy side, benign/malignant tumour type, treatment centre, and baseline differences. In all analyses, the open group is used as reference group. ^*^ The number of patients in Norway and Germany were too small to perform separate analyses on. | |
| ***Supplementary Table 5:* Subgroup analysis. Multivariable adjusted differences of global health scale per country of patients undergoing laparoscopic or open hemihepatectomy in the mITT population.** | |

## Supplementary Table 6: Subgroup demographics. Recurrence and adjuvant systemic therapy at 12 months of patients with malignant disease undergoing laparoscopic or open hemihepatectomy in the mITT population.

| Characteristic | **LH**  **(n = 136)** | **OH**  **(n = 145)** |
| --- | --- | --- |
| Adjuvant systemic therapy (%) | 35 (26) | 31 (21) |
| Death (%) | 14 (10) | 14 (10) |
| Recurrence (%) | 31 (23) | 45 (31) |
| Treatment of recurrence (%) |  |  |
| Systemic treatment | 19 (14) | 19 (13) |
| Repeat resection | 5 (4) | 13 (9) |
| Systemic treatment and repeat resection | 1 (1) | 0 |
| Ablation | 3 (2) | 3 (2) |
| Radiotherapy | 0 | 1 (1) |
| No treatment | 3 (2) | 9 (6) |
| Data are n (%). LH, laparoscopic hemihepatectomy; OH, open hemihepatectomy. | | |
| *Supplementary Table 6:* Subgroup demographics. Recurrence and adjuvant systemic therapy at 12 months of patients with malignant disease undergoing laparoscopic or open hemihepatectomy in the mITT population. | | |

## Supplementary Table 7: 90-day mortality causes

|  | **LH**  **(n = 166)** | **OH**  **(n = 166)** | **RR (95% CI)** | **OR (95% CI)** |
| --- | --- | --- | --- | --- |
| Serious Adverse Events |  |  |  |  |
| Mortality | 5 (3) | 5 (3) | 1 (1-1) | 0 (0-0) |
| Multi-organ failure | 2 (1) | 3 (2) | 0·67 (0·11 to 3·95) | -0·01 (-0·03 to 0·02) |
| Disease progression | 1 (1) | 0 | 0 | 0·01 (-0·01 to 0·02) |
| Respiratory failure | 2 (1) | 0 | 0 | 0·01 (-0·00 to 0·03) |
| Liver failure | 0 | 1 (1) | - | -0·01 (-0·02 to 0·01) |
| Cardiac arrest | 0 | 1 (1) | - | -0·01 (-0·02 to 0·01) |
| Intensive Care Admittance | Not recorded | Not recorded | - |  |
| Admission >10 days | 16 (10) | 24 (15) | 0·67 (0·37 to 1·21) | -0·05 (-0·12 to 0·02) |
| Readmission <30 days | 13 (8) | 12 (7) | 1·08 (0·51 to 2·30) | 0·01 (-0·05 to 0·06) |
| Permanent or serious invalidity | 0 (0) | 0 (0) | 0 | - |
| Reoperation | 12 (7) | 10 (6) | 1·20 (0·53 to 2·70) | 0·01 (-0·04 to 0·07) |
| Intervention | 9 (5) | 9 (5) | 1 (1-1) | 0 (0-0) |
| Adverse Events of Special Interest |  |  |  |  |
| Pleural effusion | 10 (6) | 11 (7) | 0·91 (0·40 to 2·08) | -0·01 (-0·06 to 0·05) |
| Ascites | 6 (4) | 7 (4) | 0·86 (0·29 to 2·50) | -0·01 (-0·05 to 0·04) |
| Deep wound infection^*^ | 1 (1) | 3 (2) | 0·33 (0·11 to 1·03) | -0·01 (-0·04 to 0·01) |
| Superficial wound infection^*^ | 2 (1) | 4 (2) | 0·50 (0·09 to 2·70) | -0·01 (-0·04 to 0·02) |
| Intra-abdominal abscess | 6 (4) | 6 (4) | 1 (1-1) | 0 (0-0) |
| Bile leakage | 4 (2) | 7 (4) | 0·57 (0·17 to 1·92) | -0·02 (-0·06 to 0·02) |
| Sepsis | 4 (2) | 8 (5) | 0·50 (0·15 to 1·63) | -0·02 (-0·06 to -0·00) |
| Post-hepatectomy liver failure | 6 (4) | 5 (3) | 1·20 (0·37 to 3·86) | 0·01 (-0·03 to 0·04) |
| Pneumonia^*^ | 2 (1) | 3 (2) | 0·67 (0·11 to 3·95) | -0·01 (-0·03 to 0·02) |
| Intra-abdominal hemorrhage | 2 (1) | 1 (1) | 2·00 (0·19 to 21·86) | 0·01 (-0·01 to 0·03) |
| Intra-abdominal hematoma^*^ | 0 (0) | 2 (1) | - | -0·01 (-0·03 to 0·00) |
| Acute renal failure^$^ | 0 (0) | 0 (0) | - | - |
| Blood transfusion | 15 (9) | 21 (13) | 0·71 (0·38 to 1·33) | -0·04 (-0·10 to 0·03) |
| Surgical mortality | 0 (0) | 0 (0) | - | - |
| Treatment Emergent Adverse Events |  |  |  |  |
| Cardiac arrest | 0 | 1 (1) | - | -0·01 (-0·02 to 0·01) |
| Gastroparesis | 0 | 3 (2) | - | -0·02 (-0·04 to 0·00) |
| Post-operative ileus | 2 (1) | 1 (1) | 2·00 (0·19 to 21·86) | 0·01 (-0·01 to 0·03) |
| Pulmonary embolus | 2 (1) | 0 | 0 | 0·01 (-0·00 to 0·03) |
| Data are n (%). LH, laparoscopic hemihepatectomy; OH, open hemihepatectomy.^*^ Minor Adverse events (Clavien-Dindo grade 1 or 2) are very likely to be underreported. ^$^ Acute renal failure was not reported as a separate complications, it was however reported in the accumulation of multiorgan failure. | | | | |
| *Supplementary Table 7:* 90-day mortality causes of patients undergoing laparoscopic or open hemihepatectomy in the mITT population. | | | | |

## Supplementary Table 8: Median HRQoL outcomes at baseline for patients undergoing either laparoscopic or open hemihepatectomy with multiple imputation in the mITT population

| **HRQoL outcome** | **LH**  **(n = 166)** | **OH**  (n = 166) |
| --- | --- | --- |
| **QLQ-C30 – global health status** | 75 (67 to 83) | 75 (67 to 83) |
| **QLQ-C30 - functional scales** |  |  |
| Physical functioning | 93 (87 to 100) | 100 (80 to 100) |
| Role functioning | 100 (67 to 100) | 100 (83 to 100) |
| Emotional functioning | 83 (67 to 92) | 83 (67 to 100) |
| Cognitive functioning | 100·0 (83 to 100) | 100 (83 to 100) |
| Social functioning | 100·0 (67 to 100) | 100 (67 to 100) |
| **QLQ-C30 - symptom scales** |  |  |
| Fatigue^*^ | 11 (0 to 33) | 11 (0 to 33) |
| Nausea and vomiting | 0 | 0 |
| Pain^*^ | 0 (0 to 17) | 0 (0 to 17) |
| Dyspnoea | 0 (0 to 33) | 0 (0 to 0·0) |
| Insomnia | 0 (0 to 33) | 0 (0 to 33·3) |
| Appetite Loss | 0 (0 to 33) | 0 |
| Constipation | 0 | 0 |
| Diarrhoea | 0 | 0 |
| Financial difficulties | 0 | 0 |
| **QLQ-LMC21 - symptom scales** |  |  |
| Nutritional problems | 100 (83 to 100) | 100 (83 to 100) |
| Fatigue^*^ | 11 (0 to 33) | 11 (0 to 33) |
| Pain^*^ | 0 (0 to 22) | 0 (0 to 22) |
| Emotional problems | 25 (8 to 42) | 25 (17 to 50) |
| Weight loss | 0 (0 to 33) | 0 |
| Taste | 0 | 0 |
| Dry mouth | 0 (0 to 33) | 0 (0 to 33) |
| Sore mouth | 0 | 0 |
| Peripheral neuropathy | 0 (0 to 33) | 0 (0 to 33) |
| Jaundice | 0 | 0 |
| Contact with friends | 0 | 0 |
| Talking about feelings | 0 (0 to 33) | 0 (0 to 33) |
| Sex life | 0 (0 to 33) | 0 (0 to 33) |
| Data are median (IQR). LH, laparoscopic hemihepatectomy; OH, open hemihepatectomy. **^*^** The domains fatigue and pain occur in both the EORTC-QLQ-C30 and the EORTC QLQ-LMC21. | | |
| ***Supplementary Table 8:* HRQoL outcomes at baseline for patients undergoing undergoing laparoscopic or open hemihepatectomy in the mITT population. with multiple imputation.** | | |

## Supplementary Table 9: Distribution of patients per centre.

| **Centre** | **LH**  **(n = 166)** | **OH**  (n = 166) |
| --- | --- | --- |
| Aachen | 4 (2) | 3 (2) |
| Aintree | 4 (2) | 2 (1) |
| Amsterdam | 12 (7) | 12 (7) |
| Birmingham | 20 (12) | 17 (10) |
| Brussels | 5 (3) | 5 (3) |
| Ghent | 24 (15) | 26 (16) |
| Hasselt | 1 (1) | 0 |
| London | 5 (3) | 7 (4) |
| Kortrijk | 6 (4) | 5 (3) |
| Milan | 44 (27) | 41 (25) |
| Maastricht | 8 (5) | 8 (5) |
| Newcastle | 1 (1) | 1 (1) |
| Oslo | 6 (4) | 7 (4) |
| Oxford | 1 (1) | 1 (1) |
| Plymouth | 8 (5) | 9 (5) |
| Southampton | 19 (11) | 20 (12) |
| Data are n (%), median (IQR) or mean ± SD. LH, laparoscopic hemihepatectomy; OH, open hemihepatectomy. | | |
| ***Supplementary Table 9:* Distribution of patients per centre.** | | |

# Supplementary Figures

## Supplementary Figure 1: EORTC-QLQ-C30 not selected symptom scales over the period of baseline to 12 months after after either laparoscopic or open hemihepatectomy in the mITT population


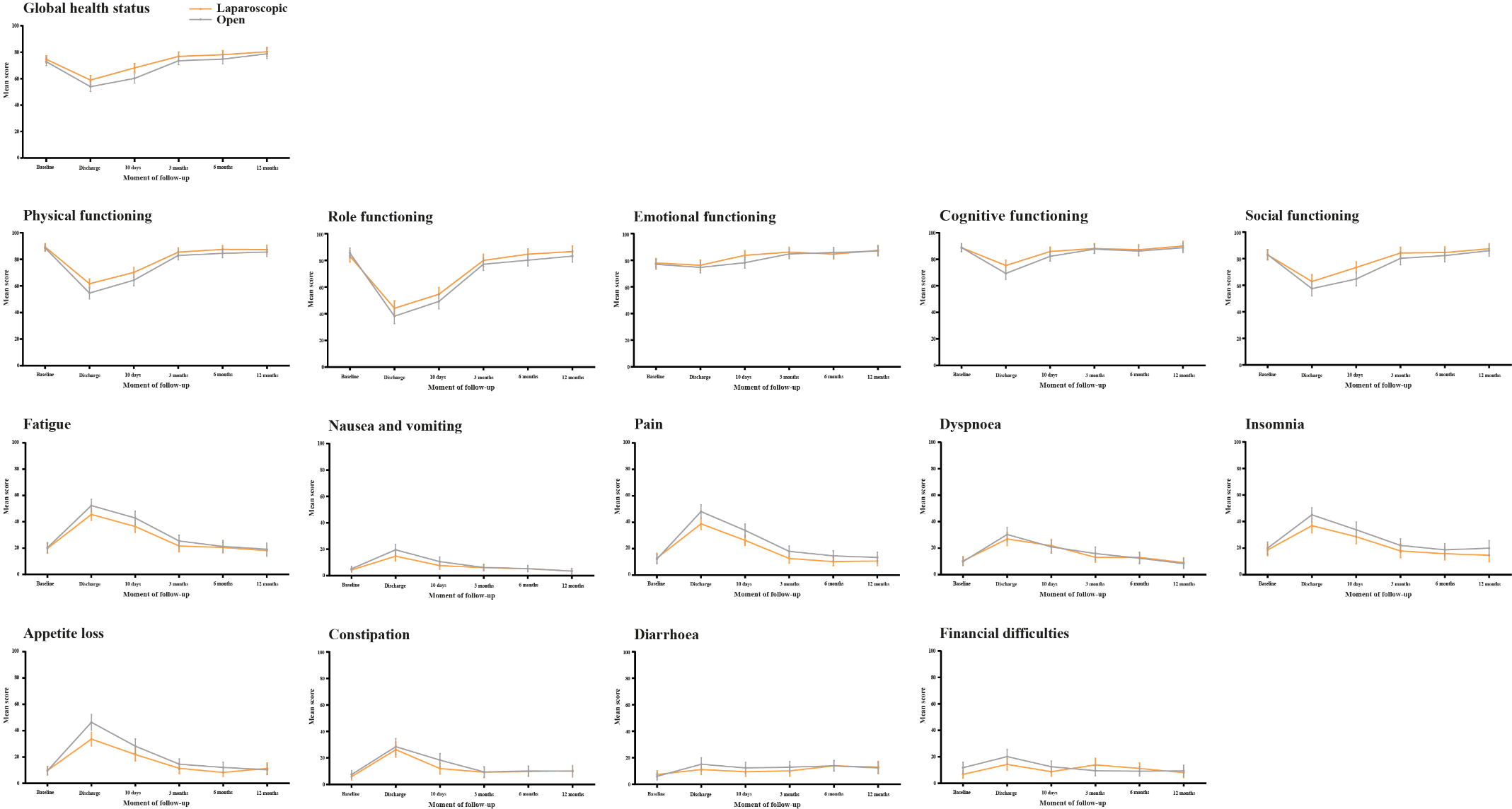


## Supplementary Figure 2: EORTC-QLQ-LMC21 not selected symptom scales over the period of baseline to 12 months after after either laparoscopic or open hemihepatectomy in the mITT population


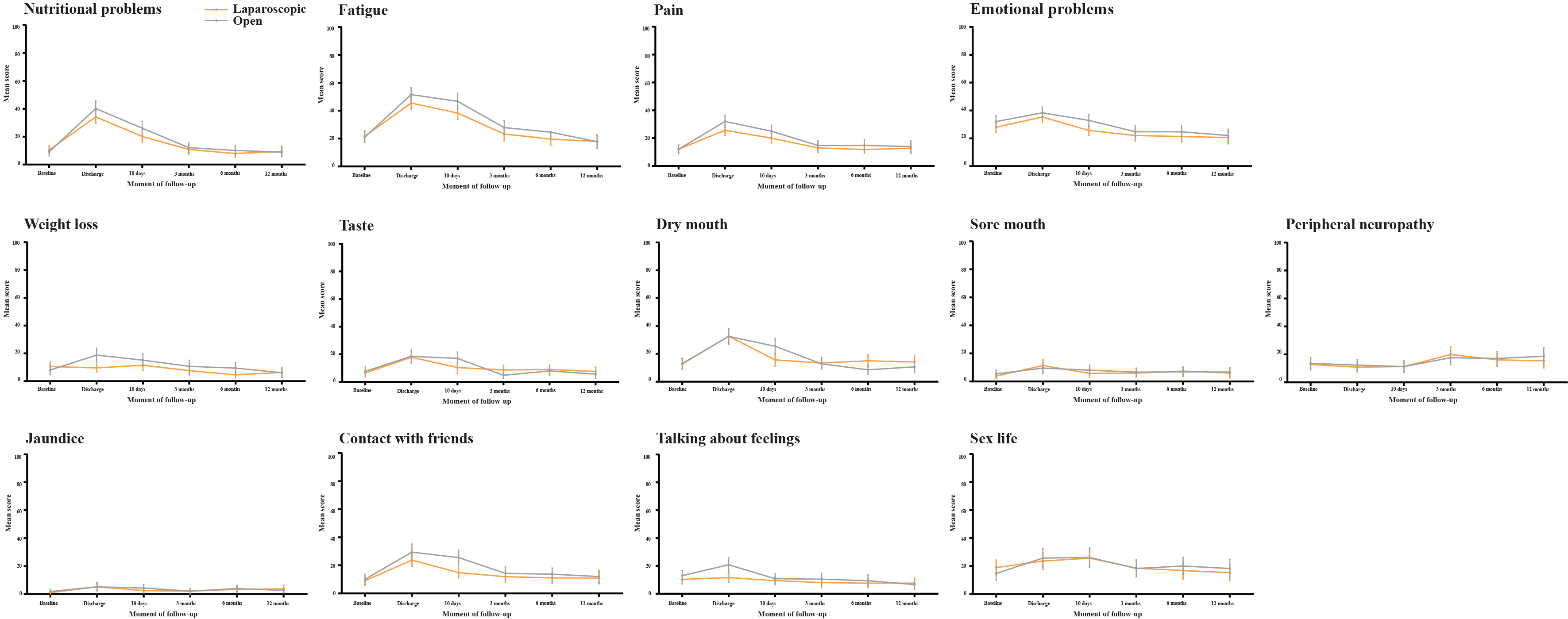


# **Supplementary List of collaborators**

ORANGE II PLUS collaborative

| **ORANGE II PLUS collaborative for acknowledgment** | | |
| --- | --- | --- |
| **Centre** | **Name** | **Role** |
| Aachen University Hospital  Aachen, Germany | Ulf Neumann | Principal investigator |
|  | Florian Ulmer | Medical staff involved in patient care |
| Aintree University Hospital NHS Foundation Trust  Aintree, United Kingdom | Rafael Díaz-Nieto | Principal investigator |
|  | Michelle Lintforth | Research nurse |
| Amsterdam University Medical Centres  Amsterdam, The Netherlands | Marc Besselink | Principal investigator |
|  | Pieter Tanis | Medical staff involved in patient care |
|  | Burak Gorçek | PhD candidate |
| University Hospitals Birmingham NHS Foundation Trust  Birmingham, United Kingdom | Robert Sutcliffe | Principal investigator |
|  | Ravi Marudanayagam | Medical staff involved in patient care |
|  | Penelope Rogers | Research nurse |
| Erasmus Hospital, Brussels, Belgium | Valerio Lucidi | Principal investigator |
|  | Viviane van Laethem | Research nurse |
| Ghent University Hospital  Ghent, Belgium | Roberto Troisi | Principal investigator |
|  | Frederik Berrevoet | Medical staff involved in patient care |
|  | Vincenzo Scuderi | Medical staff involved in patient care |
|  | Aude Vanlander | Medical staff involved in patient care |
|  | Betsy van Loo | Research nurse, trial coordinator |
|  | Kathleen Segers | Research nurse |
| Jessa Hospital  Hasselt, Belgium | Gregory Sergeant | Principal investigator |
| Groeninge General Hospital Kortrijk, Belgium | Mathieu D’Hondt | Principal investigator |
|  | Celine Demeyere | Research nurse |
| King’s College Hospital NHS Foundation Trust  London, United Kingdom | Krishna Menon | Principal investigator |
|  | Ane Zamalloa | Research nurse |
| Maastricht University Medical Centre+  Maastricht, The Netherlands | Ronald van Dam | Principal investigator, trial leader and main investigator |
|  | Cornelis Dejong | Medical staff involved in patient care |
|  | Maxime Dewulf | Medical staff involved in patient care |
|  | Lloyd Brandts | Trial statistician |
|  | Remon Korenblik | PhD candidate |
|  | Robert Fichtinger | PhD candidate, trial coordinator |
|  | Bram Olij | PhD candidate |
|  | Merel Kimman | Health economics and Quality of life expert |
| San Raffaele Hospital Milan, Italy | Luca Aldrighetti | Principal investigator |
|  | Francesca Ratti | Medical staff involved in patient care |
| Newcastle upon Tyne Hospitals NHS Foundation Trust  Newcastle, United Kingdom | Steve White | Principal investigator |
| Oslo University Hospital  Oslo, Norway | Björn Edwin | Principal investigator |
|  | Åsmund Fretland | Medical staff involved in patient care |
|  | Davit Aghayan | PhD candidate |
| Oxford University Hospitals NHS Foundation Trust  Oxford, United Kingdom | Zahir Soonawalla | Principal investigator |
|  | Katherine Gordon-Quayle | Research nurse |
| University Hospitals Plymouth NHS Foundation Trust  Plymouth, United Kingdom | Somaiah Aroori | Principal investigator |
|  | Tracy Ward | Research nurse |
| University Hospital Southampton NHS Foundation Trust  Southampton, United Kingdom | John Primrose | Principal investigator |
|  | Mohammed Abu Hilal | Principal investigator |
|  | Christopher Kümmerli | PhD candidate |
|  | Jess Boxal | Research nurse |
|  | Beth Wedge | Research nurse |
| Southampton Clinical Trials Unit  Southampton, United Kingdom | Zina Eminton | Trial coordinator |

1 Cnaan, A., Laird, N. M. & Slasor, P. Using the general linear mixed model to analyse unbalanced repeated measures and longitudinal data. *Stat Med* **16**, 2349-2380, doi:10.1002/(sici)1097-0258(19971030)16:20<2349::aid-sim667>3.0.co;2-e (1997).

2 Coens, C. *et al.* International standards for the analysis of quality-of-life and patient-reported outcome endpoints in cancer randomised controlled trials: recommendations of the SISAQOL Consortium. *Lancet Oncol* **21**, e83-e96, doi:10.1016/S1470-2045(19)30790-9 (2020).

3 Twisk, J., de Boer, M., de Vente, W. & Heymans, M. Multiple imputation of missing values was not necessary before performing a longitudinal mixed-model analysis. *J Clin Epidemiol* **66**, 1022-1028, doi:10.1016/j.jclinepi.2013.03.017 (2013).

4 Cocks, K. *et al.* Evidence-based guidelines for interpreting change scores for the European Organisation for the Research and Treatment of Cancer Quality of Life Questionnaire Core 30. *Eur J Cancer* **48**, 1713-1721, doi:10.1016/j.ejca.2012.02.059 (2012).
